# Cell-Specific Roles of Angiopoietin-2 in CKD

**DOI:** 10.1681/ASN.0000001089

**Published:** 2026-04-14

**Authors:** An-Jie Luo, Tyng-Shiuan Gau, Ming-Tsun Tsai, Szu-Yuan Li, Shu-Yi Huang, Chia-Lang Hsu, Wei-Chou Lin, Kuo-How Huang, Kai-Chien Yang, Wen-Chih Chiang, Shu-Wha Lin, Fan-Chi Chang, Shuei-Liong Lin

**Affiliations:** 1 Graduate Institute of Physiology, College of Medicine, National Taiwan University, Taipei, Taiwan; 2 Renal Division, Department of Internal Medicine, National Taiwan University Hospital, Taipei, Taiwan; 3 Renal Division, Department of Internal Medicine, Taipei Veterans General Hospital, Taipei, Taiwan; 4 School of Medicine, National Yang Ming Chiao Tung University, Taipei, Taiwan; 5 Department of Medical Research, National Taiwan University Hospital, Taipei, Taiwan; 6 Department of Pathology, National Taiwan University Hospital, Taipei, Taiwan; 7 Department of Urology, National Taiwan University Hospital, Taipei, Taiwan; 8 Graduate Institute of Pharmacology, College of Medicine, National Taiwan University, Taipei, Taiwan; 9 Department of Clinical Laboratory Sciences and Medical Biotechnology, College of Medicine, National Taiwan University, Taipei, Taiwan; 10 Research Center for Developmental Biology and Regenerative Medicine, National Taiwan University, Taipei, Taiwan

**Keywords:** CKD, endothelium, macrophages, metabolism, kidney fibrosis

## Abstract

**Key Points:**

- Global deletion of angiopoietin-2 protected against the progression of CKD.
- Endothelial cell–derived angiopoietin-2 drove early inflammation by promoting endothelial activation.
- Tubular epithelial cell–derived angiopoietin-2 contributed to functional decline and kidney fibrosis in advanced CKD.

**Background:**

CKD is driven by inflammation, vascular dysfunction, and fibrosis, with emerging evidence implicating angiopoietin-2 as a key mediator. While angiopoietin-2 is produced by endothelial cells and upregulated in injured tubular epithelial cells (TECs), the distinct contributions of endothelial cell–derived and TEC-derived angiopoietin-2 remain unclear. This study defines the cell type–specific roles of angiopoietin-2 in CKD pathogenesis.

**Methods:**

We examined kidney transcriptomes and outcomes from the Taipei Renal Transcriptomics and Outcomes Investigation cohort, with fibrosis assessed by histology and RNA sequencing. Global, endothelial cell–specific, and TEC-specific angiopoietin-2 knockout mice were subjected to experimental CKD. Kidney injury, inflammation, vascular changes, and fibrosis were evaluated using histologic, molecular, and imaging analyses.

**Results:**

In patients with CKD, elevated kidney *ANGPT2* mRNA was associated with lower estimated glomerular filtration rate, greater kidney fibrosis, and adverse kidney outcomes. Global deletion of angiopoietin-2 in mice preserved kidney function and reduced inflammation, vascular rarefaction, and fibrosis during CKD progression induced by an adenine diet. Transcriptomic profiling revealed suppression of proinflammatory and profibrotic pathways and enhancement of peroxisomal lipid metabolism. Endothelial cell–specific deletion of angiopoietin-2 attenuated early inflammatory signaling and endothelial activation but failed to prevent late-stage vascular rarefaction and fibrosis. By contrast, TEC-specific deletion preserved kidney function and reduced fibrotic and vascular injury in late-stage CKD, without impacting early inflammation and endothelial activation. Mechanistically, angiopoietin-2 promoted macrophage recruitment and matrix deposition through cell-specific pathways, without directly altering metabolism of TECs.

**Conclusions:**

This study identifies angiopoietin-2’s contribution to CKD pathogenesis through distinct roles of endothelial and tubular epithelial sources. Endothelial deletion mainly reduced early inflammation, while tubular epithelial deletion limited progressive injury and advanced fibrosis.

**Podcast:**

This article contains a podcast at https://dts.podtrac.com/redirect.mp3/www.asn-online.org/media/podcast/JASN/2026_06_18_ASN0000001089.mp3

## Visual Abstract

**Figure:**
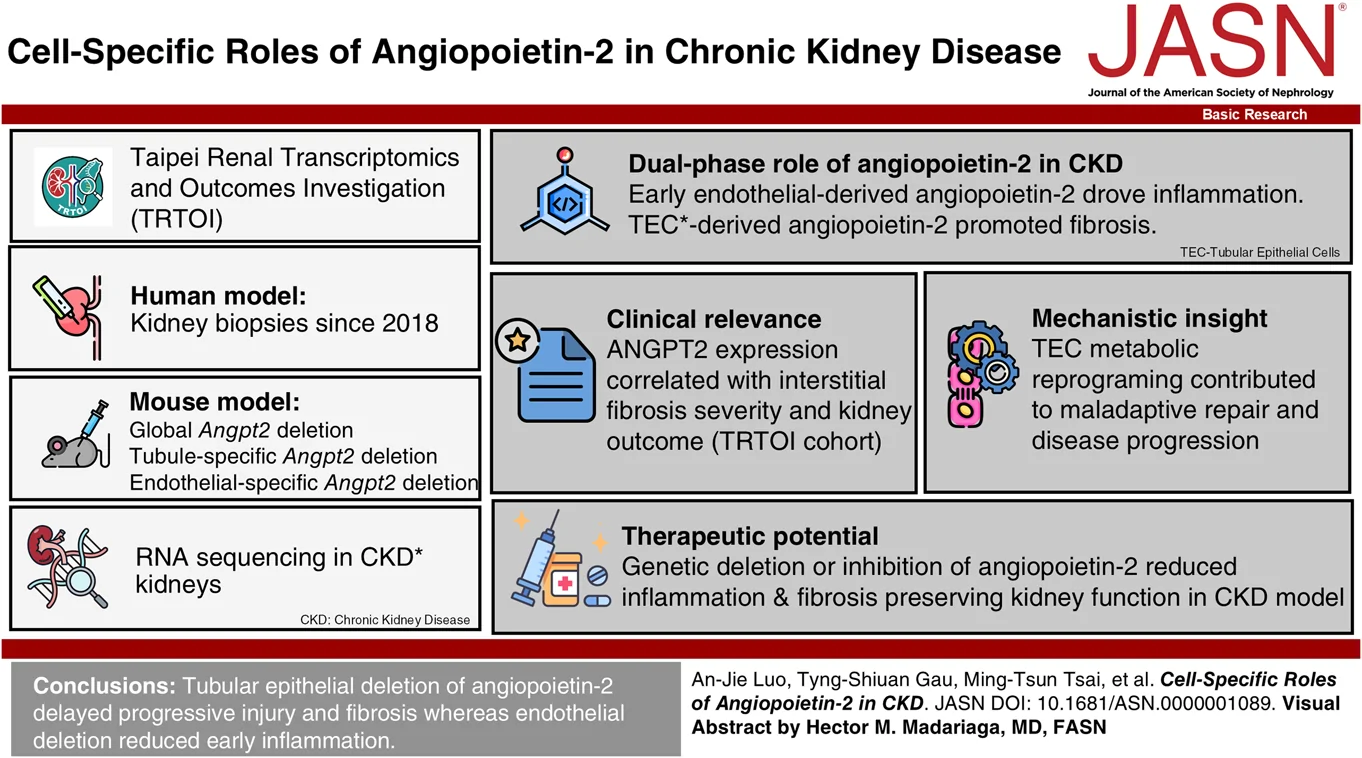

## Introduction

Kidney diseases are a global health concern, affecting more than 850 million individuals worldwide.^1,2^ Despite diverse inciting etiologies, CKD is characterized by the progressive loss of kidney function driven by common pathologic mechanisms, including inflammation, vascular dysfunction, and fibrosis.^3–5^ Among the key signaling pathways implicated in vascular homeostasis, the angiopoietins/Tie-2 axis—primarily regulated by angiopoietin-1 and angiopoietin-2—plays a central role in maintaining endothelial integrity and quiescence.^6,7^ Although angiopoietin-2 has traditionally been regarded as a competitive antagonist of angiopoietin-1, emerging evidence suggests that it acts as a context-dependent mediator of vascular destabilization and inflammation.^6,8–11^ Beyond its association with cardiovascular complications in dialysis patients,^12–14^ elevated angiopoietin-2 levels have also been linked to endothelial dysfunction, vascular instability, and inflammatory responses in various kidney pathologies.^15–20^ Nevertheless, the mechanistic role of angiopoietin-2 in the pathogenesis of CKD remains poorly understood.

Angiopoietin-2 is stored in Weibel–Palade bodies and rapidly released from endothelial cells on activation, thereby amplifying autocrine endothelial destabilization.^21,22^ Our previous studies showed that angiopoietin-2 expression is minimal in glomerular endothelial cells and in the brush border of tubular epithelial cells (TECs) in normal kidneys but is markedly upregulated in both injured endothelial cells and TECs in fibrotic kidneys induced by subtotal nephrectomy or unilateral ureteral obstruction (UUO), suggesting a broader role beyond the endothelium in kidney fibrosis.^15,17^ In murine models of UUO and unilateral ischemia-reperfusion injury, angiopoietin-2 inhibition *via* transgenic overexpression of human angiopoietin-1 attenuated chemokine expression in endothelial cells, reduced macrophage infiltration during early injury, and ameliorated fibrosis at later stages.^18^ Furthermore, recent evidence indicates that Tie-2 activation using a human angiopoietin-2–binding and Tie-2–activating antibody significantly protects UUO-induced CKD mice from peritubular capillary loss and tubulointerstitial fibrosis.^23^ These findings collectively support a pathogenic role for angiopoietin-2 in driving endothelial activation and the ensuing inflammatory and fibrotic responses in CKD. However, the distinct cellular contributions of endothelial cell–derived versus TEC-derived angiopoietin-2 to CKD progression and kidney dysfunction remain to be fully elucidated.

In this study, we aimed to elucidate the cell type–specific roles of angiopoietin-2 in CKD progression using global, endothelial cell–specific, and TEC-specific knockout models. We hypothesized that endothelial cell–derived and TEC-derived angiopoietin-2 differentially contribute to early endothelial activation and inflammatory responses, as well as to progressive kidney dysfunction, vascular rarefaction, and fibrogenesis. We further proposed that defining the cell type–specific actions of angiopoietin-2 would clarify its mechanistic role in CKD progression and help inform the rationale for future therapeutic targeting.

## Methods

Detailed methods used in this study are provided in Supplemental Material.

### Taipei Renal Transcriptomics and Outcomes Investigation

Taipei Renal Transcriptomics and Outcomes Investigation (TRTOI) is an ongoing prospective observation study conducted at Taipei Veterans General Hospital, Taiwan, as previously described.^24,25^ Consecutive adult patients (age ≥20 years) who underwent kidney biopsies or nephrectomies have been enrolled since October 2018. Demographic data, medical history, laboratory results, and clinical outcomes were extracted from electronic medical records. Kidney function was assessed using the Modification of Diet in Renal Disease equation to estimate eGFR. All kidney biopsies were reviewed and reported by the same nephropathologist. The severity of interstitial fibrosis, expressed as percentage, was evaluated using Masson trichrome staining. The primary outcomes included KRT, a sustained ≥50% decline in baseline eGFR, and death due to cardiovascular or kidney diseases. All participants were followed until the occurrence of primary outcomes, loss to follow-up, or the end of this study (February 2, 2025), whichever came first. Patients lost to follow-up were excluded from the outcome evaluation. Data analysis and outcome evaluation were conducted between September 2024 and February 2025. Transcriptomic analysis of human kidney samples was conducted as previously reported.^25^

### Animals

*Angpt2*^*loxP/loxP*^ mice were generated in our laboratory (Supplemental Figure 1). *Tg(UBC-CreERT2)* mice (JAX stock no. 007001) were obtained from The Jackson Laboratory.^26^ *Tg(UBC-CreERT2)*;*Angpt2*^*loxP/loxP*^ mice were bred to facilitate global angiopoietin-2 deletion upon tamoxifen (T5648, Sigma-Aldrich, St. Louis, MO) administration and were hereafter referred to as Angpt2^ΔUBC^. Littermates lacking the Cre transgene served as control mice. Tamoxifen was administered *via* oral gavage at a dose of 0.4 mg/g body weight to 8-week-old mice, including both the *Tg(UBC-CreERT2)*; *Angpt2*^*loxP/loxP*^ and control groups. To minimize potential interference from tamoxifen in the induction of kidney disease, a 2-week washout period was implemented after tamoxifen administration.

*Tg(Pax8-rtTA*; *LC1)* double transgenic mice were generously provided by R. Koesters (Heidelberg, Germany).^27^ *Tg(Pax8-rtTA*; *LC1)*; *Angpt2*^*loxP/loxP*^ mice were bred to enable TEC-specific angiopoietin-2 deletion on doxycycline administration and were hereafter referred to as Angpt2^ΔTEC^. Littermates lacking the Cre transgene served as control mice. Doxycycline (Sigma-Aldrich) was administered in drinking water at a concentration of 0.2 mg/ml, supplemented with 2% sucrose, starting at 8 weeks of age. In the kidney disease model, doxycycline administration began in 8-week-old mice, including both *Tg(Pax8-rtTA*; *LC1)*; *Angpt2*^*loxP/loxP*^ and control groups, 1 week before kidney disease induction and continued until the end of the experiment.

*Tg(Cdh5-CreERT2)* mice were generously provided by R.H. Adams (Münster, Germany).^28^ *Tg(Cdh5-CreERT2)*; *Angpt2*^*loxP/loxP*^ mice were bred to achieve endothelial cell–specific angiopoietin-2 deletion on tamoxifen administration and were hereafter referred to as Angpt2^ΔEC^. Littermates lacking the Cre transgene served as control mice. Tamoxifen was administered *via* oral gavage at a dose of 0.4 mg/g body weight to 8-week-old mice, including both the *Tg(Cdh5-CreERT2)*; *Angpt2*^*loxP/loxP*^ and control groups. To minimize potential interference from tamoxifen in the induction of kidney disease, a 2-week washout period was implemented after tamoxifen administration.

### Mouse CKD Models

In the first model, CKD was induced by feeding adult mice LabDiet 5001 (TestDiet, St. Louis, MO) containing 0.25% adenine (Sigma–Aldrich) for 5 days, followed by regular LabDiet 5001 for the remaining 2 days each week, a regimen modified from our previous study.^29^ Mice were euthanized 1–4 weeks after initiating the adenine diet. Alternatively, an early inflammation model of adenine-induced CKD was established by administering adenine (50 mg/kg body weight) *via* oral gavage for 7 consecutive days. Adenine was dissolved in 5% sodium carboxymethyl cellulose (Sigma–Aldrich).

In the second model, CKD was induced through UUO surgery.^18^ Under anesthesia with ketamine/xylazine (100/10 mg per kg body weight), the left ureter was ligated using a 4-0 nylon surgical suture at the level of the lower pole of the kidney, with the mice in a prone position. Sham surgery, involving a left flank incision, ureter exposure, and repositioning without ligation, served as the control. Mice were euthanized 4 days after surgery.

### L1-10 Administration

L1-10 was administered daily *via* subcutaneous injection at a dose of 5 mg/kg body weight (Amgen, Inc., Thousand Oaks, CA) to *C57BL/6* mice starting from the day adenine gavage began, to block angiopoietin-2 binding to the Tie-2 receptor.^18^ The control mice received subcutaneous injections of sFc protein (Bethyl Laboratories Inc., Montgomery, TX).

### Fluorescence-Activated Cell Sorting Analysis

Kidney samples were collected from Angpt2^ΔUBC^ mice and littermate controls after 3 weeks of dietary intervention. Mice were divided into three groups: (*1*) control mice on a regular diet, (*2*) control mice on an adenine diet, and (*3*) Angpt2^ΔUBC^ mice on an adenine diet. Kidney cells were dissociated using the Multi-Tissue Dissociation Kit 2 following standard protocols. Single-cell suspensions were stained with 4′,6-diamidino-2-phenylindole (to exclude dead cells), anti-Ly6C-APC (128031, BioLegend), anti-Ly6G-Buv395 (565964, BD Biosciences), anti-NK1.1-Buv395 (564144, BD Biosciences), and anti-CD11b-PE (101208, BioLegend). Data were acquired using a BD LSRFortessa flow cytometer (BD Biosciences), and analysis was performed using FlowJo V10 software (BD Biosciences). Gating involved selecting live cells (forward scatter [FSC]/side scatter), identifying single cells (FSC-Area/FSC-Height), excluding dead cells (4′,6-diamidino-2-phenylindole^+^), Ly6G^+^ and NK1.1^+^ populations, and identifying CD11b^high^ cells, which were further classified into Ly6C^+^ and Ly6C^−^ subpopulations.^30^ Cell counts and percentages were compared among groups, with statistical analyses performed to assess differences.

### Statistics

In the clinical study, the associations between kidney *ANGPT2* mRNA levels and eGFR or interstitial fibrosis were assessed using the Pearson correlation test. Associations with clinical outcomes were evaluated using unadjusted Cox proportional hazards regression. All statistical analyses were performed using STATA SE version 16.0 (Stata Corp., College Station, TX), with a two-sided significance level of 0.05.

For *in vivo* and *in vitro* experiments, distributional assumptions were evaluated using the Shapiro–Wilk test (primary), supported by Anderson–Darling, D'Agostino–Pearson, and Kolmogorov–Smirnov tests, together with *Q*–*Q* plots. For datasets with sample size <8, group comparisons were performed using the Mann–Whitney *U* test or the Kruskal–Wallis test. For other datasets, when raw values were skewed, data were log-transformed and analyzed using an unpaired *t *test with Welch correction. A Mann–Whitney sensitivity analysis was performed to confirm the robustness of the conclusions. Reporting of statistical assumptions and methods adheres to the Animal Research: Reporting of *In Vivo* Experiments 2.0 guidelines. All analyses for experimental data were conducted using GraphPad Prism version 10.4.1 (GraphPad Software, San Diego, CA).

### Study Approval

All animal experiments were conducted in accordance with protocols approved by the Institutional Animal Care and Use Committee of the National Taiwan University College of Medicine (20201053, 20201140, 20201162, 20220428, 20230126). The TRTOI cohort study was approved by the Institutional Review Board of Taipei Veterans General Hospital (2018-06-008B). All patients provided written informed consent. The clinical study protocol followed the principles outlined in the Declaration of Helsinki.

## Results

### Kidney *ANGPT2* mRNA Levels Were Higher in CKD and Associated with Adverse Kidney Outcomes

In the TRTOI cohort, an ongoing prospective observational study at Taipei Veterans General Hospital (Figure 1A),^25^ kidney *ANGPT2* mRNA levels were higher in patients with CKD and were negatively correlated with eGFR (Figure 1B, *N*=127). By contrast, angiopoietin-1 (*ANGPT1*) mRNA levels were not correlated with either eGFR or kidney fibrosis (Figure 1, C and E, *N*=127), whereas kidney *ANGPT2* mRNA levels were positively correlated with the degree of kidney fibrosis (Figure 1D, *N*=127). In Cox proportional hazards analysis, higher *ANGPT2* expression was associated with a greater risk of the composite kidney outcome (hazard ratio [HR], 1.79; 95% confidence interval [CI], 1.15 to 2.79 for *ANGPT2*; HR, 1.01; 95% CI, 0.91 to 1.13 for *ANGPT1*), initiation of KRT (HR, 1.96; 95% CI, 1.24 to 3.11 for *ANGPT2*; HR, 1.01; 95% CI, 0.91 to 1.13 for *ANGPT1*), and death (HR, 1.68; 95% CI, 1.08 to 2.59 for *ANGPT2*; HR, 0.99; 95% CI, 0.87 to 1.11 for *ANGPT1*; Figure 1F, *n*=101). These results indicate that increased kidney *ANGPT2* mRNA levels were associated with lower kidney function, greater fibrosis, and a higher risk of adverse clinical outcomes in CKD.

**Figure 1:**
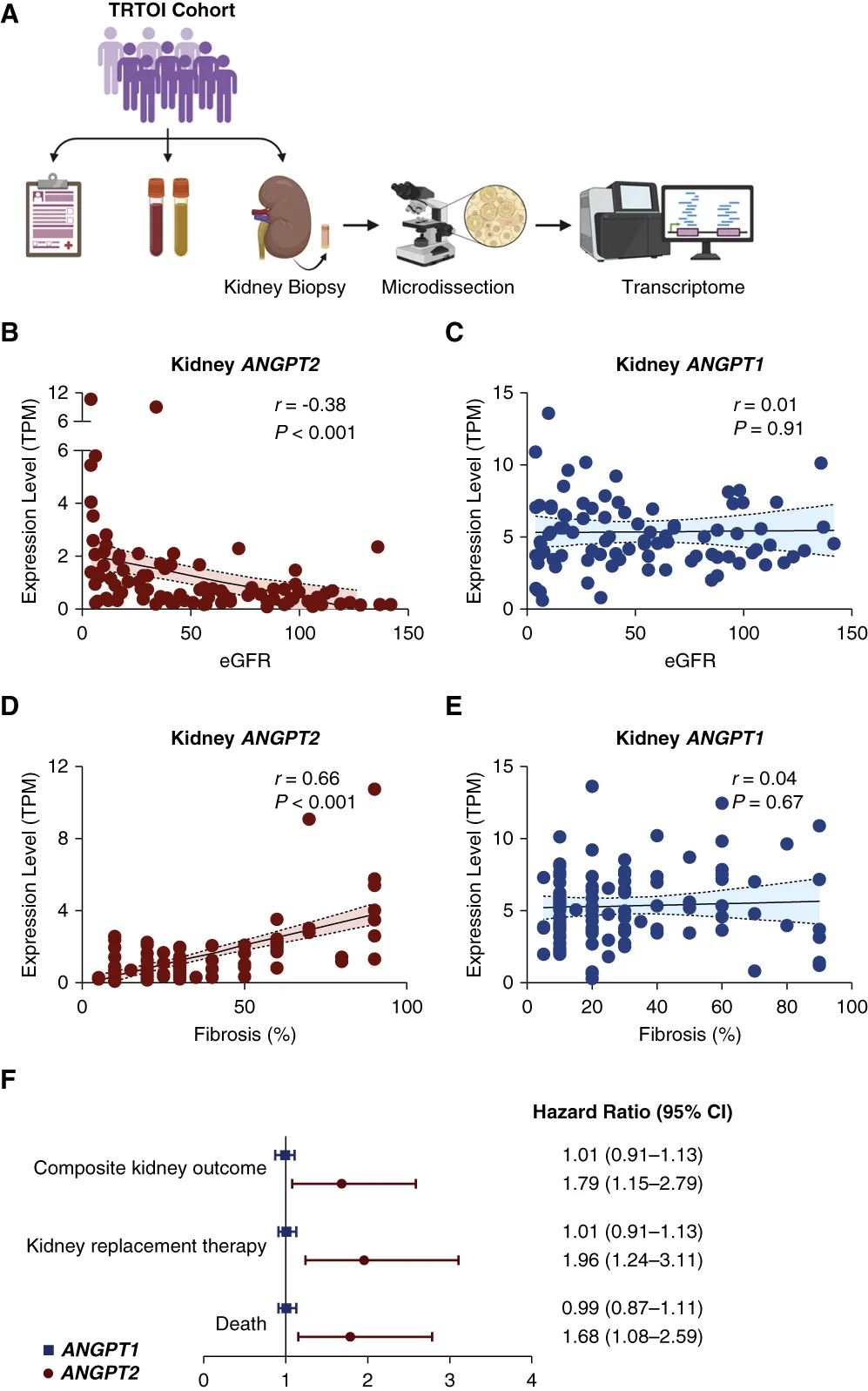
**Higher angiopoietin-2 levels in the kidneys of patients with CKD.** (A) Overview of the TRTOI cohort and study workflow. Biopsied kidney tissues were microdissected and subjected to RNA-Seq. Clinical data and outcomes were prospectively recorded. (B–E) Scatter plots illustrating correlations between kidney *ANGPT2* and *ANGPT1* mRNA levels and eGFR in (B) and (C), respectively, as well as their correlations with interstitial fibrosis in (D) and (E), respectively. Pearson correlation was used to assess statistical significance. *N*=127. (F) Forest plot showing the associations between kidney *ANGPT1* and *ANGPT2* mRNA levels and clinical outcomes, including composite kidney outcome, KRT, and death. Cox proportional hazard models were used. *n*=101. Composite kidney outcome was defined as initiation of KRT or a sustained ≥50% decline from baseline eGFR. *ANGPT1,* angiopoietin-1; CI, confidence interval; RNA-Seq, RNA sequencing; TPM, transcripts per million; TRTOI, Taipei Renal Transcriptomic and Outcomes Investigation.

### Global Deletion of Angiopoietin-2 Ameliorated Kidney Damage in Advanced Adenine-Induced CKD

Because deletion of angiopoietin-2 in all epiblast cells by embryonic day 6.5 resulted in a higher risk of intrauterine fetal death (Supplemental Table 1), we used Angpt2^ΔUBC^ mice to achieve global deletion of angiopoietin-2 before the induction of CKD with an adenine-containing diet (Figure 2, A and B). No significant differences were observed between littermate controls lacking Cre recombinase (Control) and Angpt2^ΔUBC^ mice 2 weeks after tamoxifen administration (Supplemental Table 2). Compared with Control mice, Angpt2^ΔUBC^ mice exhibited improved kidney function, as reflected by lower plasma levels of BUN and creatinine starting from the third week after CKD induction (Figure 2C). Histologic analysis revealed reduced tubulointerstitial injury (Figure 2, D and E), vascular rarefaction (Figure 2, F and G, and Supplemental Figure 2), macrophage infiltration (Figure 2, H–J), and interstitial fibrosis (Figure 2, K–M) in Angpt2^ΔUBC^ mice. These findings indicate that upregulation of angiopoietin-2 contributed to kidney dysfunction and structural injury in adenine-induced CKD.

**Figure 2:**
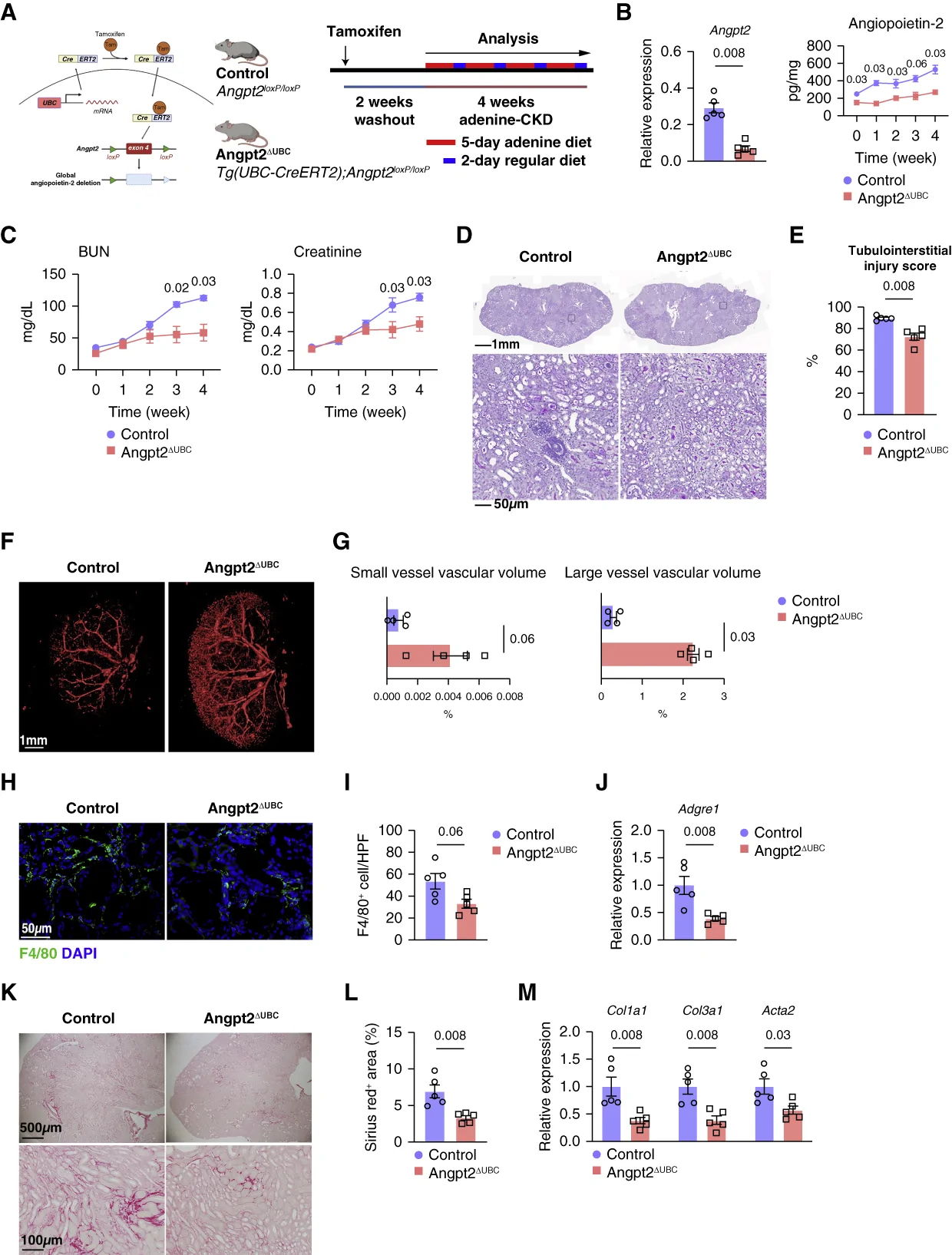
**Global angiopoietin-2 deletion mitigates functional impairment, tubulointerstitial injury, vascular rarefaction, and fibrosis in adenine-induced CKD.** (A) Experimental schematic. Tamoxifen-induced deletion of *Angpt2* exon 4 in *Tg(UBC-CreERT2)*;*Angpt2*^*loxP/loxP*^ mice (Angpt2^ΔUBC^) with littermates lacking Cre serving as controls (Control). The timeline illustrates tamoxifen administration and induction of adenine-induced CKD. The weekly regimen comprised a 5-day adenine diet followed by a 2-day regular diet to establish CKD. Kidneys were analyzed 4 weeks after CKD induction. (B) Bar graph and XY line graph showing the relative expression levels of *Angpt2* mRNA (left, normalized to *Gapdh*) and angiopoietin-2 protein (right, normalized to kidney weight) in kidney tissues. (C) XY line graphs depicting plasma levels of BUN and creatinine at baseline (week 0) and 1–4 weeks after CKD induction. (D) Representative images of PAS-stained kidney sections. Enlarged views of the boxed areas are shown below. Scale bars: 1 mm (original magnification 1×) and 50 *μ*m (original magnification 200×). (E) Bar graph showing tubulointerstitial injury scores quantified from PAS-stained kidney sections at 200× magnification. (F) Representative *ex vivo μ*CT images of Microfil-perfused kidneys with 3D reconstructions. Scale bar: 1 mm. (G) Bar graphs showing *μ*CT-based quantification of vascular volume. Small vessels were defined as those with a diameter <40 *μ*m, while large vessels had a diameter ≥40 *μ*m. (H) Representative images of F4/80^+^ macrophages with DAPI nuclear counterstain in kidney sections. Scale bar: 50 *μ*m (original magnification 400×). (I) Bar graph depicting the number of F4/80^+^ macrophages per high-power field at 400× magnification. (J) Bar graph showing the relative expression levels of *Adgre1*, normalized to *Gapdh*, in kidney tissues. *Adgre1* encodes adhesion G protein–coupled receptor E1 (ADGRE1, also known as F4/80). (K) Representative images of picrosirius red–stained fibrillar collagen in kidney sections. Scale bars: 500 *μ*m (original magnification 40×) and 100 *μ*m (original magnification 200×). (L) Bar graph showing morphometric quantification of picrosirius red–stained areas in kidney sections at 200× magnification. (M) Bar graphs presenting the relative expression levels of *Col1a1*, *Col3a1*, and *Acta2*, normalized to *Gapdh*, in kidney tissues. *Col1a1*, *Col3a1*, and *Acta2* encode type I collagen *α*1 chain, type III collagen *α*1 chain, and *α*-smooth muscle actin, respectively. Data are expressed as means±SEM, with each dot in the bar graphs representing an individual mouse (B, C, E, G, I, J, L, and M). Statistical significance was determined using two-tailed Mann–Whitney *U* test. *n*=4–5 mice per group in (B–M). 3D, 3-dimensional; *Adgre1*, adhesion G protein–coupled receptor E1 also known as F4/80; *Angpt2*, angiopoietin-2; DAPI, 4′,6-diamidino-2-phenylindole; GAPDH, glyceraldehyde-3-phosphate dehydrogenase; HPF, high-power field; PAS, periodic acid–Schiff; *μ*CT, micro-computed tomography.

### Global Deletion of Angiopoietin-2 Mitigated Metabolic Derangement in Advanced Adenine-Induced CKD

To uncover the molecular mechanisms underlying the protective effects of angiopoietin-2 deletion, we performed RNA sequencing (RNA-Seq) on kidney tissues from Control and Angpt2^ΔUBC^ mice 3 weeks after adenine-induced CKD. Principal component analysis revealed distinct transcriptomic profiles in Angpt2^ΔUBC^ kidneys compared with Control kidneys (Figure 3A). Ingenuity pathway analysis showed significant upregulation of metabolic pathways in Angpt2^ΔUBC^ kidneys, particularly peroxisomal lipid metabolism, fatty acid *β*-oxidation, triglyceride metabolism, and peroxisome proliferator–activated receptor signaling (Figure 3B). Gene Ontology biologic process analysis indicated enrichment of categories related to energy substrate metabolism, amino acid and organic acid metabolism, mitochondrial energy metabolism, cofactor and redox balance, and transport (Figure 3C), suggesting higher bioenergetic activity in Angpt2^ΔUBC^ kidneys. Notably, peroxisomal, rather than mitochondrial, *β*-oxidation was preferentially upregulated (Figure 3D). Key peroxisomal molecules, including *Abcd3* (encoding 70-kDa peroxisomal membrane protein), *Acox1* encoding peroxisomal acyl-coenzyme A oxidase 1, and *Cat* (encoding catalase), were significantly increased at both the transcript and protein levels (Figure 3, E–G, and Supplemental Figure 3). Immunofluorescence confirmed increased expression of peroxisomal acyl-coenzyme A oxidase 1, catalase, and 70-kDa peroxisomal membrane protein in TECs, as demonstrated by colocalization with the basement membrane marker nidogen (Figure 3H). By contrast, both *in vivo* and *in vitro* studies showed that TECs lacked Tie-2 expression, and treatment with exogenous angiopoietin-2 did not alter metabolic gene expression in primary TECs (Supplemental Figure 4). These results indicate that angiopoietin-2 deletion promoted metabolic reprogramming in TECs, characterized by enhanced peroxisomal lipid catabolism and a shift toward a protective bioenergetic state, through an indirect mechanism that remains to be fully defined.

**Figure 3:**
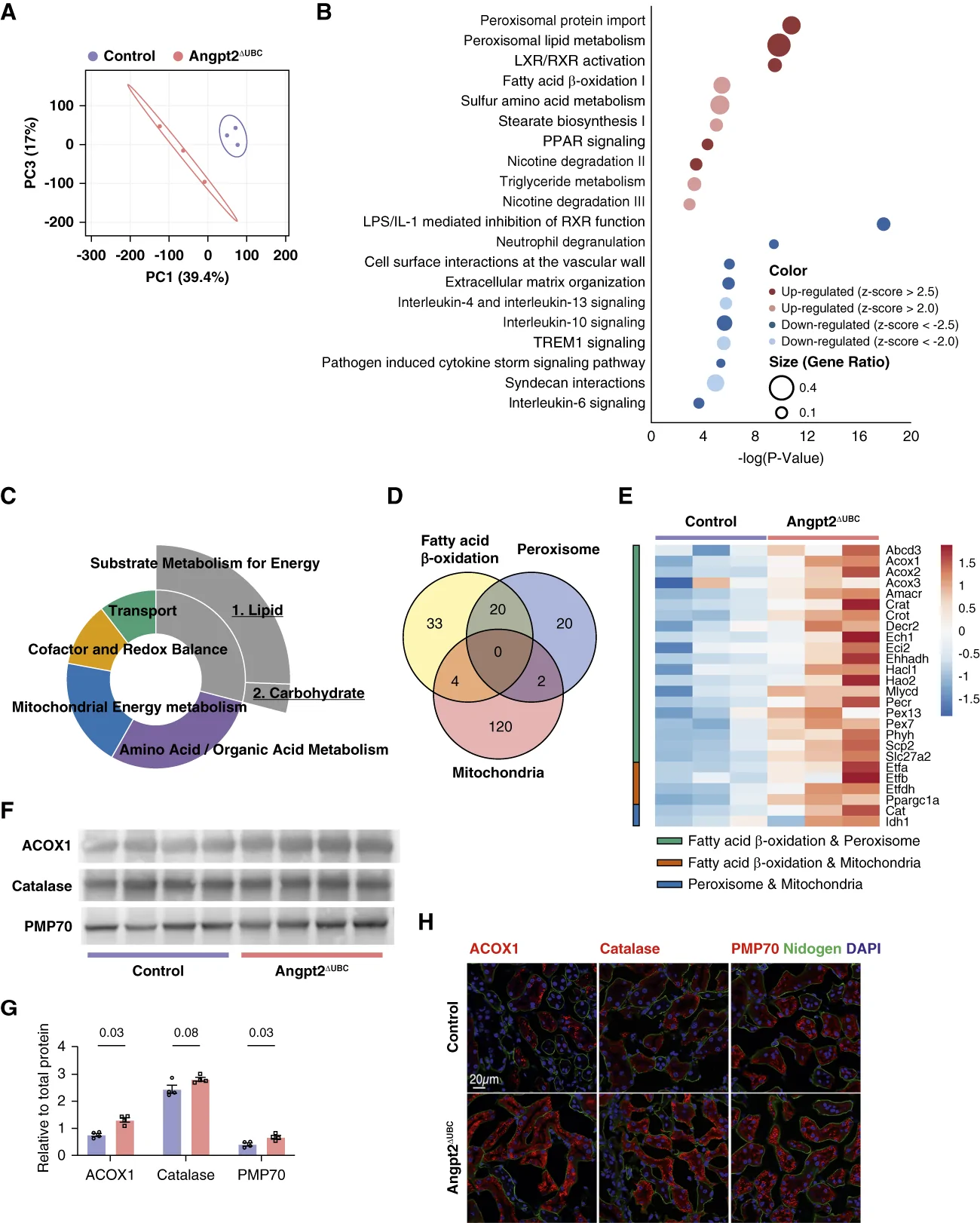
**Global angiopoietin-2 deletion mitigates metabolic derangement in advanced adenine-induced CKD.** (A) Principal component analysis of kidney transcriptomes from Control and Angpt2^△UBC^ mice 3 weeks after adenine-induced CKD. *n*=3 mice per group. (B) Ingenuity pathway analysis of RNA-Seq data showing significantly upregulated and downregulated pathways in Angpt2^△UBC^ mice compared with Control mice (thresholds: *z*-score >2 or < −2; −log_10_[*P* value] > 2). (C) Functional categorization of 86 significantly upregulated GOBP terms, based on metabolic signatures. Pathways were grouped into substrate metabolism for energy (25 pathways), amino acid/organic acid metabolism (25 pathways), mitochondrial energy metabolism (17 pathways), cofactor and redox balance (ten pathways), and transport (nine pathways). The substrate metabolism for energy category was further subclassified into lipid and carbohydrate metabolism. (D) Venn diagram displaying shared genes among fatty acid *β*-oxidation, mitochondrial, and peroxisomal pathways. (E) Heatmap of differentially expressed genes from shared gene sets identified in (D), comparing Control and Angpt2^△UBC^ mice. Red: upregulated; blue: downregulated. (F) Western blot analysis of ACOX1, catalase, and PMP70 in kidney tissue lysates from Control and Angpt2^ΔUBC^ mice 3 weeks after adenine-induced CKD. (G) Bar graph showing the relative expression of ACOX1, catalase, and PMP70, normalized to total protein. (H) Representative immunofluorescence images of ACOX1, catalase, and PMP70, costained with Nidogen (a basement membrane marker) in kidney sections. Nuclei were counterstained with DAPI. Scale bar: 20 *μ*m (original magnification 400×). Data are expressed as means±SEM, with each dot representing an individual mouse. Statistical significance was determined using two-tailed Mann–Whitney *U* test. *n*=4 mice per group in G. ACOX1, peroxisomal acyl-coenzyme A oxidase 1; GOBP, Gene Ontology biologic process; LPS, lipopolysaccharide; LXR, liver X receptor; PMP70, 70-kDa peroxisomal membrane protein; PPAR, peroxisome proliferator–activated receptor; RXR, retinoid X receptor; TREM, triggering receptor expressed on myeloid cells.

### Global Deletion of Angiopoietin-2 Attenuated Inflammatory Signaling and Reduced Macrophage Infiltration in Progressive Adenine-Induced CKD

Inflammatory pathways, including neutrophil degranulation and triggering receptor expressed on myeloid cells signaling, and fibrosis-related pathways, such as extracellular matrix organization and cell surface interactions at the vascular wall, were downregulated in Angpt2^ΔUBC^ kidneys compared with Control kidneys (Figure 3B). Gene Ontology biologic process analysis indicated that inflammation- and adhesion-related categories were significantly downregulated in Angpt2^ΔUBC^ kidneys (Figure 4A). Consistently, expression of key proinflammatory chemokines, cytokines, and adhesion molecules—including *Ccl2*, *Cx3cl1*, *Ccl17*, *Csf1*, *Ccr2*, *Cx3cr1*, *Icam1*, and *Vcam1*—was significantly reduced in Angpt2^ΔUBC^ kidneys (Figure 4, B and D, and Supplemental Figure 5). Flow cytometry analysis revealed elevated CD11b^high^ macrophage populations in Control kidneys following adenine-induced CKD, which were significantly attenuated in Angpt2^ΔUBC^ kidneys (Figure 4, E and F, and Supplemental Figure 6). Moreover, CD11b^high^ macrophages sorted from Angpt2^ΔUBC^ kidneys exhibited reduced expression of proinflammatory (*Il1b*) and profibrotic (*Ccl17*, *Ccl22*) markers, suggesting suppression of both proinflammatory and profibrotic macrophage subsets (Figure 4G). Stratification by Ly6C expression revealed that both CD11b^high^Ly6C^+^ and CD11b^high^Ly6C^−^ populations were significantly reduced in Angpt2^ΔUBC^ kidneys (Figure 4, H and I). These findings indicate that angiopoietin-2 deficiency limited macrophage recruitment and activation, thereby attenuating inflammation and fibrosis in CKD.

**Figure 4:**
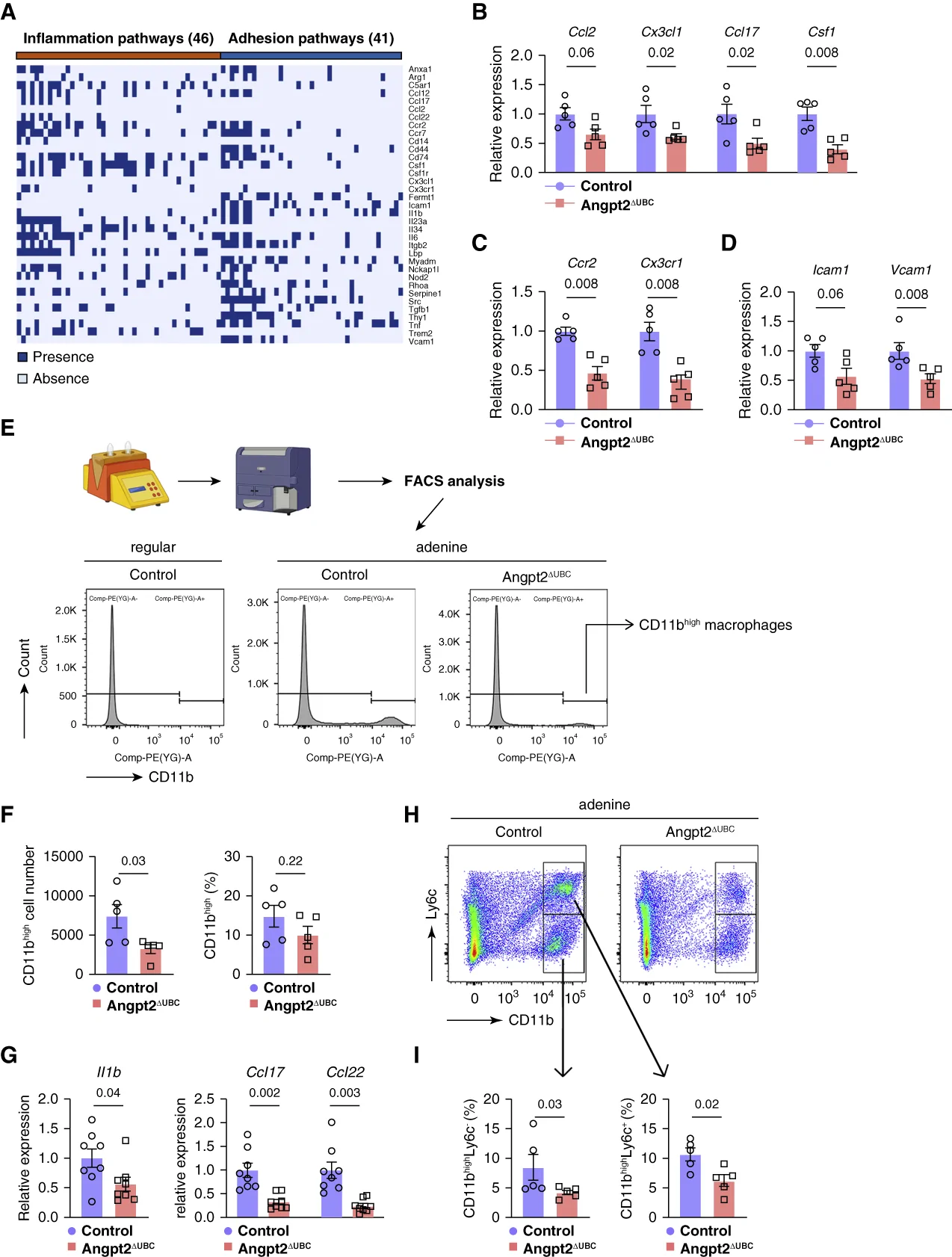
**Global angiopoietin-2 deletion suppresses inflammatory signaling and reduces infiltration of macrophages in advanced adenine-induced CKD.** (A) Group heatmap of significantly downregulated GOBP terms (normalized enrichment score <−2, *q* value <0.05), highlighting the top 35 associated genes. (B–D) Bar graphs showing the relative expression of proinflammatory and adhesion-related genes, normalized to *Gapdh*, in kidney tissues. Genes include *Ccl2*, *Cx3cl1*, *Ccl17*, *Csf1*, *Ccr2*, *Cx3cr1*, *Icam1*, and *Vcam1*, respectively. (E and F) Schematic outlining the FACS strategy for identifying macrophage populations in kidney tissues from Control and Angpt2^ΔUBC^ mice 3 weeks after adenine-induced CKD. Control mice on a regular chow served as non-CKD reference. Kidney macrophages were characterized by CD11b^high^ in (E). Bar graphs showing the quantification of CD11b^high^ kidney macrophages presented as absolute cell numbers (left) and percentages (right) in (F). (G) Bar graphs showing the relative expression of proinflammatory (*Il1b*) and profibrotic (*Ccl17*, *Ccl22*) cytokine/chemokine genes, normalized to *Gapdh*, in isolated CD11b^high^ kidney macrophages. *Il1b*, *Ccl17*, and *Ccl22* encode IL-1*β*, CCL17, and CCL22, respectively. (H and I) Schematic outlining the stratification of CD11b^high^ kidney macrophages by Ly6C expression in (H). Bar graphs showing the relative proportions of CD11b^high^Ly6C^−^ (left) and CD11b^high^Ly6C^+^ (right) macrophages in (I). Data are expressed as means±SEM, with each dot representing an individual mouse in (B–D, F, G, and I). Statistical significance was determined using two-tailed Mann–Whitney *U* test or unpaired *t* test with Welch correction. *n*=5 mice per group in (B–I), and *n*=8 mice per group in (G). CCL2, C-C motif chemokine ligand 2; CCR2, C-C motif chemokine receptor 2; CSF1, CCL17, colony stimulating factor 1; CX3CL1, C-X3-C motif chemokine ligand 1; CX3CR1, C-X3-C motif chemokine receptor 1; FACS, fluorescence-activated cell sorting; ICAM1, intercellular adhesion molecule 1; VCAM1, vascular cell adhesion molecule 1.

### Angiopoietin-2 Enhanced Proinflammatory and Profibrotic Effects of Endothelial Cells

CD31^+^ endothelial cells, the primary kidney cell type expressing the Tie-2 receptor, were isolated from Control and Angpt2^ΔUBC^ kidneys 3 weeks after adenine-induced CKD. Consistent with whole-kidney findings, endothelial cells from Angpt2^ΔUBC^ kidneys showed significantly reduced expression of *Angpt2* compared with Control cells (Supplemental Figure 7A). Expression of proinflammatory (*Vcam1*) and profibrotic (*Tgfb1*) genes was also reduced (Supplemental Figure 7A). Western blot analysis confirmed lower expression of *C*–*C* motif chemokine ligand 2 and VCAM1 proteins in endothelial cells from Angpt2^ΔUBC^ kidneys (Supplemental Figure 7, B and C). These findings suggest that angiopoietin-2 promoted endothelial activation and enhanced proinflammatory and profibrotic signaling in endothelial cells during adenine-induced CKD.

### TEC-Specific Deletion of Angiopoietin-2 Reduced CKD Severity

Given increased angiopoietin-2 expression in injured and proliferating TECs in murine CKD models (Supplemental Figure 8),^17,31^ we generated Angpt2^ΔTEC^ mice to achieve TEC-specific deletion of angiopoietin-2 before adenine-induced CKD (Figure 5, A and B). No significant differences were observed between littermate controls lacking Cre recombinase (Control) and Angpt2^ΔTEC^ mice 2 weeks after doxycycline administration (Supplemental Table 3). Compared with control mice, Angpt2^ΔTEC^ mice displayed reduced kidney *Angpt2* expression (Figure 5B); improved kidney function (Figure 5C); reduced tubulointerstitial injury (Supplemental Figure 9), vascular rarefaction (Figure 5, D and E), interstitial inflammation (Figure 5, F–H), and fibrosis (Figure 5, I–K); and decreased expression of endothelial adhesion molecules (Figure 5, L–N). These findings indicate that TEC-specific angiopoietin-2 deletion protected against functional and structural deterioration in progressive adenine-induced CKD.

**Figure 5:**
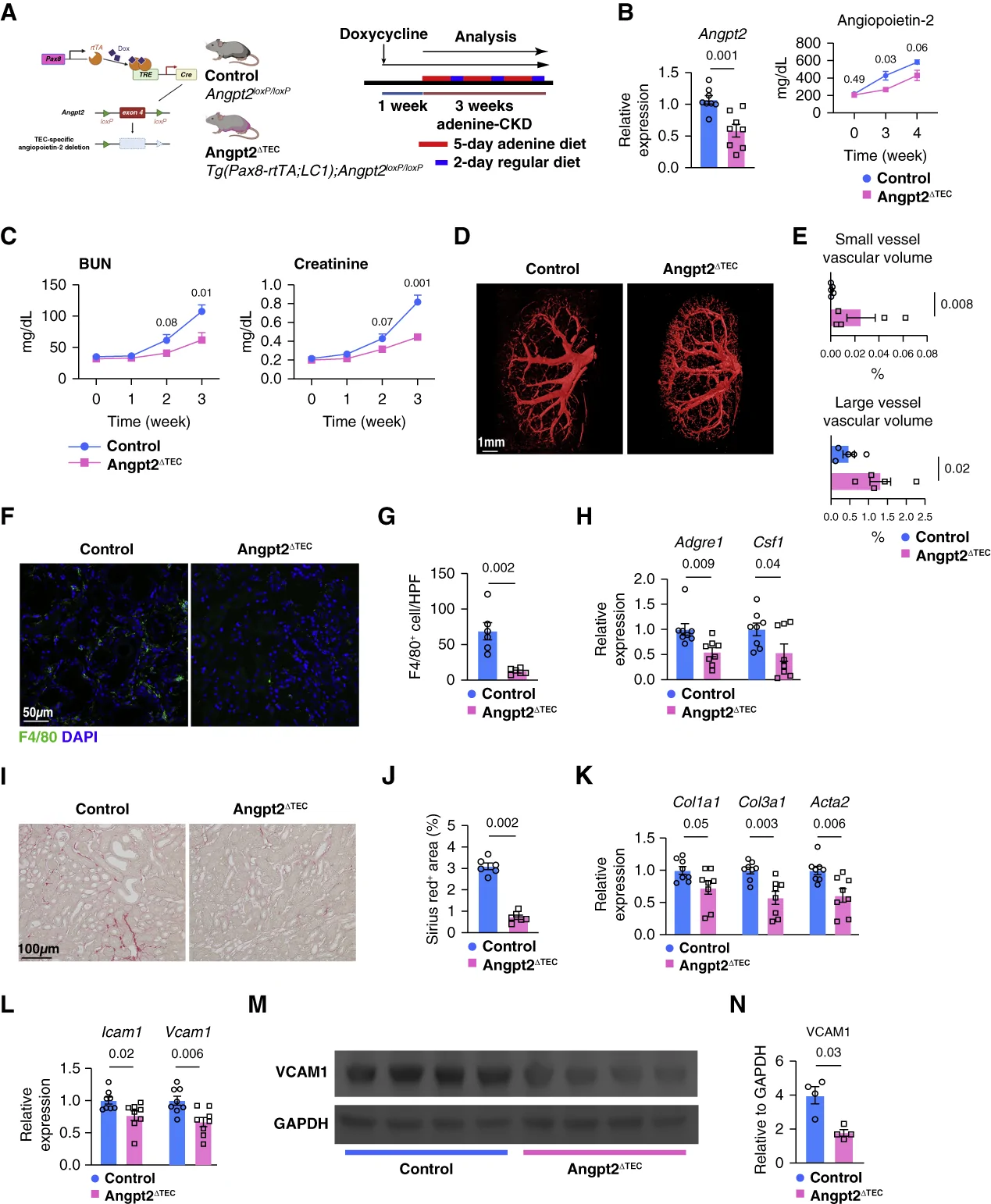
**TEC-specific angiopoietin-2 deletion mitigates functional impairment, vascular rarefaction, macrophage infiltration, and fibrosis in advanced adenine-induced CKD.** (A) Experimental schematic. Doxycycline-induced TEC-specific angiopoietin-2 deletion in *Tg*(*Pax8-rtTA*;*LC1*);*Angpt2*^*loxP/loxP*^ mice (Angpt2^ΔTEC^), with littermates lacking Cre serving as controls (Control). The timeline illustrates doxycycline administration and adenine-induced CKD. The weekly regimen comprised a 5-day adenine diet followed by a 2-day regular diet to establish CKD. Kidneys were analyzed 3 weeks after CKD induction. (B) Bar graph and XY line graph show relative expression levels of *Angpt2* mRNA (left, normalized to *Gapdh*) and angiopoietin-2 protein (right, normalized to kidney weight) in kidney tissues. (C) XY line graphs depicting plasma levels of BUN and creatinine at baseline (week 0) and 1–3 weeks after CKD induction. (D) Representative *ex vivo μ*CT images of Microfil-perfused kidneys with 3D reconstructions. Scale bar: 1 mm. (E) Bar graphs showing *μ*CT-based quantification of vascular volume. Small vessels were defined as those with a diameter <40 *μ*m, while large vessels had a diameter ≥40 *μ*m. (F) Representative immunofluorescence images of F4/80^+^ macrophages in kidney sections. Nuclei were counterstained with DAPI. Scale bar: 50 *μ*m (original magnification 400×). (G) Bar graph depicting the number of F4/80^+^ macrophages per HPF at 400× magnification. (H) Bar graphs showing the relative expression levels of *Adgre1* and *Csf1*, normalized to *Gapdh*, in kidney tissues. (I) Representative images of picrosirius red–stained fibrillar collagen in kidney sections. Scale bar: 100 *μ*m (original magnification 200×). (J) Bar graph showing morphometric quantification of picrosirius red–stained areas in kidney sections at 200× magnification. (K) Bar graphs presenting the relative expression levels of *Col1a1*, *Col3a1*, and *Acta2*, normalized to *Gapdh*, in kidney tissues. (L) Bar graphs presenting the relative expression levels of *Icam1* and *Vcam1*, normalized to *Gapdh*. (M and N) Western blot analysis and bar graph showing the relative expression of VCAM1 and GAPDH in kidney tissue lysates from Control and Angpt2^ΔTEC^ mice 3 weeks after adenine-induced CKD. Data are expressed as means±SEM, with each dot in the bar graphs representing an individual mouse in (B, E, G, H, J–L, and N). Statistical significance was determined using two-tailed Mann–Whitney *U* test or unpaired *t* test with Welch correction. *n*=4–8 mice per group in (B–N). TEC, tubular epithelial cells.

### Endothelial Cell–Specific Deletion of Angiopoietin-2 Did Not Impact Progressive Adenine-Induced CKD

Given the expression of angiopoietin-2 in endothelial cells,^17,21,22,31^ we used Angpt2^ΔEC^ mice to assess the role of endothelial cell–derived angiopoietin-2 in progressive adenine-induced CKD (Figure 6A). No significant differences were observed between littermate controls lacking Cre recombinase (Control) and Angpt2^ΔEC^ mice 2 weeks after tamoxifen administration (Supplemental Table 4). Unlike global or TEC-specific deletion, endothelial cell–specific deletion of angiopoietin-2 did not markedly reduce kidney *Angpt2* mRNA or angiopoietin-2 protein expression 3 weeks after CKD induction, although a significant reduction was observed before and 1 week after CKD induction (Figure 6B). These findings suggest that endothelial cells are not the predominant source of angiopoietin-2 at this advanced stage of disease. Moreover, endothelial cell–specific deletion did not significantly improve kidney function or histologic features (Figure 6, C and D, and Supplemental Figure 10).

**Figure 6:**
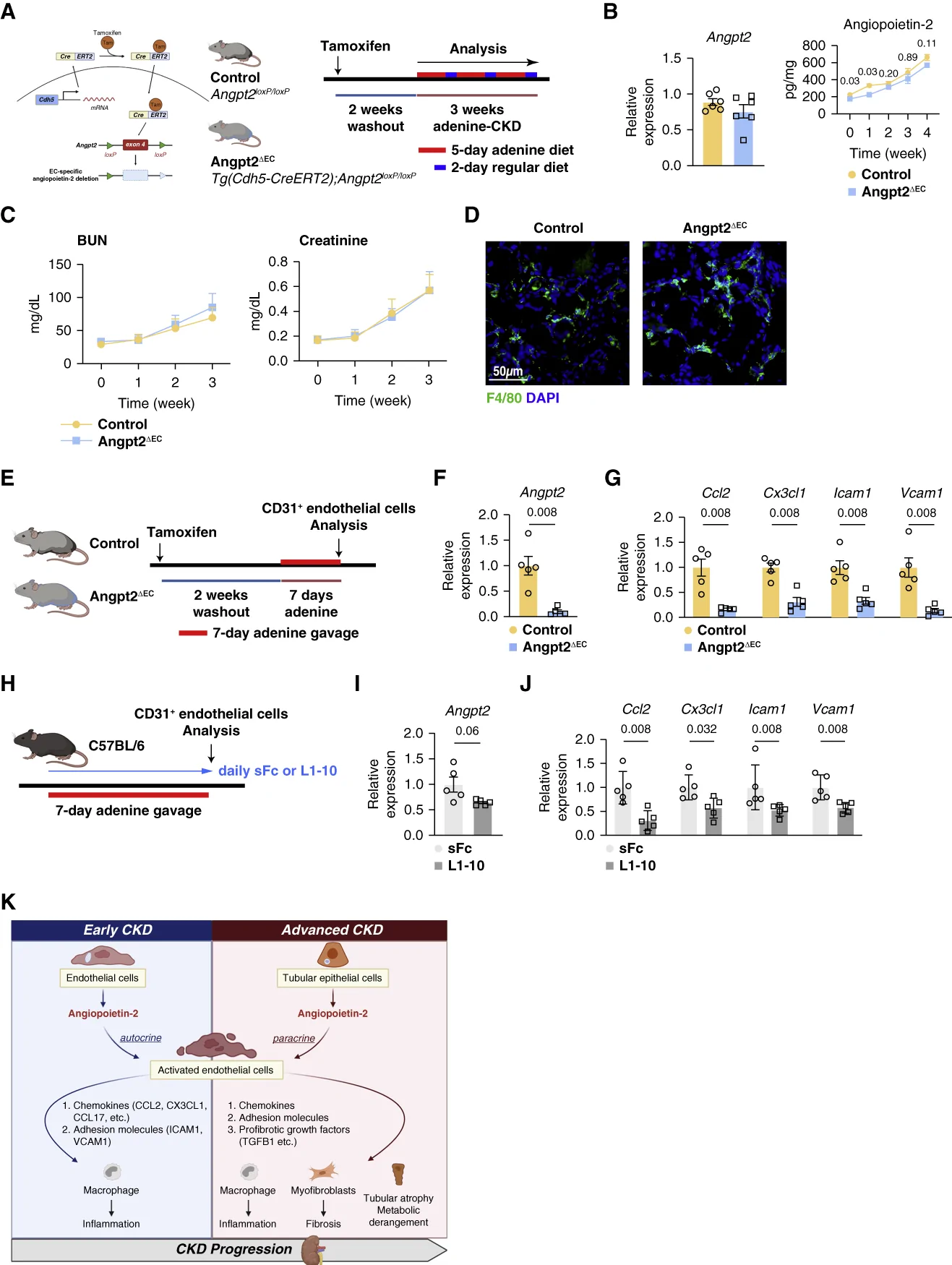
**Endothelial cell**–**specific deletion or systemic inhibition of angiopoietin-2 by L1-10 suppresses proinflammatory and proadhesive endothelial responses in early adenine-induced CKD.** (A) Experimental schematic. Tamoxifen-induced endothelial cell–specific angiopoietin-2 deletion (Angpt2^ΔEC^), with littermates lacking Cre served as controls (Control). The timeline shows tamoxifen administration and induction of adenine-induced CKD. The weekly regimen comprised a 5-day adenine diet followed by a 2-day regular diet to establish CKD. Kidneys were analyzed 3 weeks after CKD induction. (B) Bar graph and XY line graph showing the relative expression levels of *Angpt2* mRNA (left, normalized to *Gapdh*) and angiopoietin-2 protein (right, normalized to kidney weight) in kidney tissues. (C) XY line graphs showing plasma levels of BUN and creatinine at baseline (week 0) and 1–3 weeks after CKD induction. (D) Representative images of F4/80^+^ macrophages in kidney sections. Nuclei were counterstained with DAPI. Scale bar: 50 *μ*m (original magnification 400×). (E) Protocol schematic for tamoxifen administration and the induction of early adenine-induced CKD. Adenine (50 mg/kg body weight) was administered *via* oral gavage for 7 consecutive days. (F and G) Bar graphs showing the relative expression levels of *Angpt2* in (F), *Ccl2*, *Cx3cl1*, *Icam1*, and *Vcam1* in (G), normalized to *Gapdh*, in CD31^+^ endothelial cells isolated from kidneys of Control and Angpt2^ΔEC^ mice. (H) Protocol schematic for sFc fragment or angiopoietin-2 inhibitor L1-10 in early CKD induced by oral gavage of adenine (50 mg/kg body weight) for 7 consecutive days. (I and J) Bar graphs showing the relative expression levels of *Angpt2* in (I), *Ccl2*, *Cx3cl1*, *Icam1*, and *Vcam1* in (J), normalized to *Gapdh*, in CD31^+^ endothelial cells isolated from the kidneys of mice treated with sFc or L1-10. Data are expressed as means±SEM, with each dot in the bar graphs representing an individual mouse in (B, F, G, I, and J). Statistical significance was determined using two-tailed Mann–Whitney *U* test. *n*=4–5 mice per group in (B–J). (K) Proposed mechanisms of angiopoietin-2 in CKD pathogenesis. In the early stage, endothelial cell–derived angiopoietin-2 exerts autocrine effects to upregulate chemokines and adhesion molecules, thereby enhancing macrophage recruitment and vascular inflammation. In the advanced stage, vascular rarefaction ensues, and TEC-derived angiopoietin-2 further amplifies proinflammatory and profibrotic signaling, contributing to metabolic derangement and CKD progression. sFC, soluble Fc.

### Endothelial Cell–Derived Angiopoietin-2 Drove Early Inflammation in CKD

To investigate the role of endothelial cell–expressed angiopoietin-2 in early inflammation, Angpt2^ΔEC^ mice were subjected to adenine gavage for 7 consecutive days (Figure 6E). CD31^+^ endothelial cells isolated from Angpt2^ΔEC^ kidneys showed reduced expression of *Angpt2*, *Ccl2*, *Cx3cl1*, *Icam1*, and *Vcam1* genes (Figure 6, F and G), as well as their corresponding proteins (Supplemental Figure 11, A and B). Similar anti-inflammatory effects were observed after treatment with L1-10, an antiangiopoietin-2 peptide (Figure 6, H–J, and Supplemental Figure 11, C and D).

We previously demonstrated that macrophage accumulation significantly increased in the murine kidney by day 4 after UUO surgery.^15^ Here, we demonstrated that both Angpt2^ΔUBC^ and Angpt2^ΔEC^ mice exhibited reduced macrophage accumulation and lower expression of proinflammatory markers in the kidneys on day 4 postsurgery (Supplemental Figures 12 and 13), whereas Angpt2^ΔTEC^ did not affect early inflammation (Supplemental Figure 14). CD31^+^ endothelial cells isolated from both Angpt2^ΔUBC^ and Angpt2^ΔEC^ kidneys also showed reduced expression of *Angpt2* and *Icam1* (Supplemental Figure 15). These findings highlight the role of endothelial cell–derived, rather than TEC-derived, angiopoietin-2 in initiating the early inflammatory response in CKD. As capillary rarefaction progressed and the disease advanced, TEC-derived angiopoietin-2 sustained a cycle of inflammation, fibrosis, and metabolic dysregulation (Figure 6K).

## Discussion

This study defines the cell-specific and stage-specific roles of angiopoietin-2 in CKD progression. With transcriptomics of human biopsies and murine genetic models, we demonstrate that endothelial cell–derived angiopoietin-2 initiates early inflammatory responses during the onset of CKD, while TEC-derived angiopoietin-2 contributes to vascular rarefaction, fibrosis, and functional decline during the progression of disease. Our findings support a dual-phase model of CKD pathogenesis, wherein angiopoietin-2 orchestrates distinct pathologic processes through temporally and spatially regulated cellular processes, underscoring the potential of angiopoietin-2 as a therapeutic target to limit CKD progression.

Clinically, we extend previous studies of increased angiopoietin-2 expression in CKD by demonstrating, for the first time, that kidney *ANGPT2* mRNA levels are associated with interstitial fibrosis severity and adverse kidney outcomes in the independent prospective TRTOI cohort.^17,18,24^ Previous studies primarily focused on circulating plasma angiopoietin-2 levels, whereas our findings provide direct evidence at the tissue-transcript level linking kidney *ANGPT2* to disease progression. Notably, interstitial fibrosis exhibits the strongest inverse relationship with kidney function,^32^ and these results validate angiopoietin-2 as a tissue-level biomarker of progressive kidney injury and a putative driver of fibrosis in CKD.

Experimentally, global deletion of angiopoietin-2 in adult mice protected against adenine-induced CKD, reducing inflammatory cell infiltration, vascular rarefaction, and kidney fibrosis and improving kidney function. These protective effects were consistent with our previous findings in UUO- and unilateral ischemia-reperfusion injury–induced CKD models, in which pharmacologic inhibition of angiopoietin-2 using L1-10 produced similar protective effects.^18^ A recent study also reported that Tie-2 activation using a human angiopoietin-2–binding and Tie-2–activating antibody significantly protected UUO-induced CKD mice from peritubular capillary loss, tubular injury, and interstitial fibrosis.^23^ Notably, angiopoietin-2 deletion in healthy adult mice did not compromise kidney structure or function, suggesting that angiopoietin-2 is dispensable for baseline kidney homeostasis but selectively activated during disease, supporting its potential as a safe therapeutic target.

In the early phase of CKD, endothelial cell–derived angiopoietin-2 promoted endothelial activation and amplified local inflammatory signaling through autocrine upregulation of chemokines and adhesion molecules, thereby facilitating macrophage infiltration.^21,22^ Although endothelial cell–specific deletion of angiopoietin-2 attenuated these inflammatory responses, it did not prevent long-term structural and functional deterioration, suggesting that additional cellular sources contribute to sustained kidney injury. During the later stage of CKD, endothelial apoptosis drives vascular rarefaction and tissue hypoxia.^18,32–34^ Mechanistically, we previously demonstrated that endothelial cells undergo apoptosis in response to Wnt ligands and angiopoietin-2, particularly in the absence of angiopoietin-1.^18^ This vascular loss may reduce the contribution of endothelial cell–derived angiopoietin-2 to disease progression during advanced CKD.

In the advanced stage, TEC-derived angiopoietin-2 emerged as a pivotal mediator of fibrosis and organ dysfunction. Angiopoietin-2 expression shifted from endothelial cells to TECs, accompanied by suppressed angiopoietin-1 and altered metabolic gene signatures. Although TEC-specific deletion of angiopoietin-2 did not influence early macrophage infiltration, it conferred significant protection against functional impairment and pathology in advanced CKD, recapitulating the benefits observed with global deletion. These findings highlight a phenotypic switch of TECs from expressing angiopoietin-1 to producing angiopoietin-2,^17^ contributing to maladaptive repair and fibrogenesis. This advances our understanding of how injured TECs drive kidney fibrosis.^4,35^ In addition, during kidney fibrosis, the expression of key metabolic enzymes and regulators—particularly those involved in fatty acid oxidation in TECs—is significantly reduced compared with controls.^36–38^ Our findings suggest that angiopoietin-2 suppresses fatty acid oxidation in TECs through an indirect, yet-to-be-defined mechanism, promoting a proinflammatory and bioenergetically unfavorable microenvironment. The metabolic rewiring in Angpt2^ΔUBC^ kidneys likely contributed to the mitigation of CKD progression and associated metabolic disturbances.

Several limitations should be acknowledged. First, because previous studies have shown that endothelial cell–specific Tie-2 deletion leads to vascular dysfunction,^39–41^ we did not use this model in CKD mice. Consequently, we cannot exclude Tie-2–independent mechanisms contributing to CKD-associated signaling. Second, although our transcriptomic and *in vitro* data suggest that angiopoietin-2 may indirectly influence metabolic reprogramming in TECs, the precise downstream mediators remain to be identified. Third, the upstream signals driving the phenotypic switch of TECs toward angiopoietin-2 production remain incompletely defined. Finally, our inducible genetic models were designed for mechanistic dissection rather than therapeutic timing. The data delineate early endothelial and late tubular phases of angiopoietin-2 contribution, suggesting distinct therapeutic windows. Because angiopoietin-2 is acutely released from endothelial Weibel–Palade bodies and later induced transcriptionally, preinjury recombination does not preclude early angiopoietin-2 activity, consistent with the transient effect observed in Angpt2^ΔEC^. Further translational studies are required to evaluate the therapeutic potential of angiopoietin-2 inhibition in human CKD.

In conclusion, angiopoietin-2 is a dynamic regulator of CKD progression, with distinct pathogenic roles in early inflammation and late fibrosis depending on its cellular source. The differential effects of cell-specific angiopoietin-2 deletion highlight a dual-phase model of CKD pathogenesis: endothelial cell–derived angiopoietin-2 drives early inflammatory responses, whereas TEC-derived angiopoietin-2 sustains fibrosis and functional deterioration. Targeting angiopoietin-2 may provide a multifaceted therapeutic strategy to simultaneously disrupt inflammatory and fibrotic signaling pathways, offering a promising avenue for CKD intervention.

## Supplementary Material

## Data Availability

Original data generated for the study are available in a public access repository. Data Type: Raw Data/Source Data. The raw sequencing data of mouse kidney RNA have been deposited in the Sequence Read Archive of the National Center for Biotechnology Information under accession number PRJNA1281200, and the processed data are available in Gene Expression Omnibus (GEO) under accession number GSE300746. Human kidney RNA-Seq data of TRTOI generated in this study have been deposited in GEO with accession number GSE271143. The data supporting the conclusions in this study, and the experimental materials, are available from the corresponding author upon reasonable request. https://www.ncbi.nlm.nih.gov/geo/query/acc.cgi?acc=GSE300746; https://www.ncbi.nlm.nih.gov/geo/query/acc.cgi?acc=GSE271143.

## Declarative Statements

This study includes clinical experimentation and was approved by the Institutional Review Board. All patients provided written informed consent in accordance with the Declaration of Helsinki. All animal experiments were conducted in accordance with the NIH Guide for the Care and Use of Laboratory Animals or an equivalent standard. All animal protocols were approved by the Institutional Animal Care and Use Committee.

## Supplemental Material

Supplemental Methods.

Supplemental Figure 1. Targeting vector for generating *Angpt2*^*loxP/loxP*^ mice.

Supplemental Figure 2. Global angiopoietin-2 deletion preserves vascular integrity in adenine-induced CKD.

Supplemental Figure 3. Ponceau S total protein staining and lane-specific loading factors.

Supplemental Figure 4. Angiopoietin-2 does not activate downstream signaling or alter metabolic gene expression in primary TECs.

Supplemental Figure 5. Global angiopoietin-2 deletion downregulates adhesion molecules in advanced adenine-CKD.

Supplemental Figure 6. Gating strategy for kidney macrophage analysis by flow cytometry.

Supplemental Figure 7. Global deletion of angiopoietin-2 reduces proinflammatory and profibrotic factors in endothelial cells in advanced adenine-induced CKD.

Supplemental Figure 8. *Angpt2* expression in healthy and fibrotic mouse kidneys.

Supplemental Figure 9. TEC-specific angiopoietin-2 deletion reduces tubulointerstitial injury in advanced adenine-induced CKD.

Supplemental Figure 10. Endothelial cell–specific angiopoietin-2 deletion does not attenuate tubulointerstitial injury, macrophage infiltration, or kidney fibrosis in advanced adenine-induced CKD.

Supplemental Figure 11. Endothelial cell–specific angiopoietin-2 deletion reduces proinflammatory and proadhesive molecule expression in endothelial cells during early adenine-induced CKD.

Supplemental Figure 12. Global angiopoietin-2 deletion attenuates inflammatory phenotype in early UUO-induced CKD.

Supplemental Figure 13. Endothelial cell–specific angiopoietin-2 deletion attenuates inflammatory phenotype in early UUO-induced CKD.

Supplemental Figure 14. TEC-specific angiopoietin-2 deletion does not affect inflammatory phenotype in early UUO-induced CKD.

Supplemental Figure 15. Global and endothelial cell–specific angiopoietin-2 deletion attenuates proinflammatory and profibrotic phenotypes in endothelial cells during early UUO-induced CKD.

Supplemental Figure 16. Pathology report of a male *Angpt2*^*–/–*^ mouse.

Supplemental Figure 17. Pathology report of a female *Angpt2*^*–/–*^ mouse.

Supplemental Figure 18. Pathology report of a male *Angpt2*^*loxP/–*^ mouse.

Supplemental Figure 19. Pathology report of a male *Angpt2*^*loxP/loxP*^ mouse.

Supplemental Table 1. Breeding summary of offspring at 7 days of age from *Angpt2*^*loxP/–*^ parents.

Supplemental Table 2. Characteristics of control and Angpt2^ΔUBC^ mice 2 weeks after tamoxifen administration.

Supplemental Table 3. Characteristics of control and Angpt2^ΔTEC^ mice 2 weeks after doxycycline administration.

Supplemental Table 4. Characteristics of control and Angpt2^ΔEC^ mice 2 weeks after tamoxifen administration.

Supplemental Table 5. Primer sequence for the construction of targeting vector.

Supplemental Table 6. Primer sequence for quantitative PCR.

Supplemental Table 7. Primer sequence for genotyping PCR.
