# Supplementary material for: Cell-Specific Roles of Angiopoietin-2 in CKD
Source: J Am Soc Nephrol. 2026 Apr 14;37(10):2117–31. doi: 10.1681/ASN.0000001089 (PMC13641515; doi:10.1681/ASN.0000001089)
Supplement: Supplementary file 2 [file jasn-37-2117-s002.pdf]

Luo et al. Supplemental Material

Supplemental Methods

References

Supplemental Figures

Supplemental Figure 1. Targeting vector for generating *Angpt2*$^{loxP/loxP}$ mice.

Supplemental Figure 2. Global angiopoietin-2 deletion preserves vascular integrity in adenine-induced chronic kidney disease.

Supplemental Figure 3. Ponceau S total protein staining and lane-specific loading factors.

Supplemental Figure 4. Angiopoietin-2 does not activate downstream signaling or alter metabolic gene expression in primary tubular epithelial cells.

Supplemental Figure 5. Global angiopoietin-2 deletion downregulates adhesion molecules in advanced adenine-CKD.

Supplemental Figure 6. Gating strategy for kidney macrophage analysis by flow cytometry.

Supplemental Figure 7. Global deletion of angiopoietin-2 reduces pro-inflammatory and pro-fibrotic factors in endothelial cells in advanced adenine-induced CKD.

Supplemental Figure 8. *Angpt2* expression in healthy and fibrotic mouse kidneys.

Supplemental Figure 9. Tubular epithelial cell-specific angiopoietin-2 deletion reduces tubulointerstitial injury in advanced adenine-induced CKD.

Supplemental Figure 10. Endothelial cell-specific angiopoietin-2 deletion does not attenuate tubulointerstitial injury, macrophage infiltration, or kidney fibrosis in advanced adenine-induced CKD.

Supplemental Figure 11. Endothelial cell-specific angiopoietin-2 deletion reduces pro-inflammatory and pro-adhesive molecule expression in endothelial cells during early adenine-induced CKD.

Supplemental Figure 12. Global angiopoietin-2 deletion attenuates inflammatory phenotype in early UUO-induced CKD.

Supplemental Figure 13. Endothelial cell-specific angiopoietin-2 deletion attenuates inflammatory phenotype in early UUO-induced CKD.

Supplemental Figure 14. Tubular epithelial cell-specific angiopoietin-2 deletion does not affect inflammatory phenotype in early UUO-induced CKD.

Supplemental Figure 15. Global and endothelial cell-specific angiopoietin-2 deletion attenuates pro-inflammatory and pro-fibrotic phenotypes in endothelial cells during early UUO-induced CKD.

Supplemental Figure 16. Pathology report of a male $Angpt2^{-/-}$ mouse.

Supplemental Figure 17. Pathology report of a female $Angpt2^{-/-}$ mouse.

Supplemental Figure 18. Pathology report of a male $Angpt2^{loxP/-}$ mouse.

Supplemental Figure 19. Pathology report of a male $Angpt2^{loxP/loxP}$ mouse.

Supplemental Tables

Supplemental Table 1. Breeding summary of offspring at 7 days of age from $Angpt2^{loxP/-}$ parents.

Supplemental Table 2. Characteristics of Control and $Angpt2^{\Delta UBC}$ mice two weeks after tamoxifen administration.

Supplemental Table 3. Characteristics of Control and $Angpt2^{\Delta TEC}$ mice two weeks after doxycycline administration.

Supplemental Table 4. Characteristics of Control and $Angpt2^{\Delta EC}$ mice two weeks after tamoxifen administration.

Supplemental Table 5. Primer sequence for the construction of targeting vector.

Supplemental Table 6. Primer sequence for quantitative PCR.

Supplemental Table 7. Primer sequence for genotyping PCR.

Full Methods

Details of the Taipei Renal Transcriptomics and Outcomes Investigation (TRTOI)

TRTOI is a prospective observation study conducted at Taipei Veterans General Hospital, Taiwan.[1] Consecutive adult patients (age $\geq$ 20 years) who underwent kidney biopsy or nephrectomy have been enrolled since October 2018. Patients with pregnancy, prior kidney transplantation, who refused to provide consent, or who participated in other clinical studies were excluded. Blood and urine samples were collected on the day of biopsy. Standard processing of kidney specimens included light microscopy, immunofluorescence, and electron microscopy. A total of 127 patients were evaluated for the association between kidney transcriptomics, kidney function, and the severity of kidney fibrosis, with baseline characteristics previously reported by *Tsai et al*.[2] Among these, 101 patients had sufficient follow-up data for outcome analysis.

Transcriptomic analysis of human kidney samples was conducted as previously reported.[2] A fraction of renal biopsy tissues was preserved in RNAlater (Invitrogen, Carlsbad, CA, USA) and microdissected to isolate the tubulointerstitial compartment. The mRNA expression profile was obtained using the NovaSeq 6000 system (Illumina, San Diego, CA, USA), generating paired-end reads (150 base pairs in length). The processed reads were aligned to the human reference genome (GRCh37/hg19) using STAR (v2.6.1). Gene expression levels were quantified using RSEM software (v1.3.1), with transcript abundance measured in transcripts per million (TPM) for each sample.

Animals

To generate *Angpt2*$^{loxP/loxP}$ mice, a targeting vector was constructed containing exon 4 of the mouse *Angpt2* gene with a neomycin resistance cassette (Neo cassette), flanked by loxP and FRT sites, respectively (Supplemental Figure 1). The homologous arms were amplified by polymerase chain reaction (PCR) using primers (Supplemental Table 5), and recombineering techniques were employed to insert the loxP sites and the

Neo cassette. The construct was validated through restriction enzyme digestion using BamHI and NotI + EcoRI. The targeting vector was linearized by restriction enzyme digestion using NotI and SpeI, and then electroporated into embryonic stem (ES) cells to facilitate homologous recombination at the target locus. Successfully targeted ES cell clones were identified through PCR and Southern blot analysis. Validated ES cells were microinjected into *C57BL/6* blastocysts, which were then transferred into pseudopregnant recipient mice to produce chimeric offspring. Initial breeding of *Angpt2$^{loxP/loxP}$* mice was conducted at the Transgenic Mouse Core Laboratory of National Taiwan University. The resulting mice were subsequently crossed with FLP recombinase-expressing mice to excise the Neo cassette, leaving loxP sites flanking exon 4. These mice were hereafter referred to as *Angpt2$^{loxP/loxP}$* mice.

Breeding *Angpt2$^{loxP/loxP}$* male mice with *Tg(Sox2-Cre)* female mice (strain #: 008454, The Jackson Laboratory, Bar Harbor, ME), which mediated Cre excision of the floxed allele at the E6.5 epiblast stage,[3] produced offspring carrying the *Cre* transgene and a heterozygous deletion of *Angpt2* in all subsequent cell lineages. To remove the *Cre* transgene, these offspring were bred with wild-type *C57BL/6* mice, resulting in *Angpt2$^{loxP/–}$* mice. We then interbred *Angpt2$^{loxP/–}$* mice to generate *Angpt2$^{–/–}$* mice with a homozygous deletion of *Angpt2*. Notably, interbreeding *Angpt2$^{loxP/–}$* mice produced fewer *Angpt2$^{–/–}$* offspring than expected based on Mendelian inheritance (Supplemental Table 1). This deviation suggests a potential increase in intrauterine or perinatal fetal death among *Angpt2$^{–/–}$* embryos, particularly in males, highlighting a crucial role for angiopoietin-2 in embryonic survival and development. In surviving *Angpt2$^{–/–}$* mice, no visible abnormalities were observed at 10 to 11 weeks of age. However, histological examination revealed severe and diffuse ectasia of the lymphatic sinuses in the lymph nodes of both male and female *Angpt2$^{–/–}$* mice, as well as severe and diffuse interstitial edema in the testes (Supplemental Figure 16, 17), findings similar to a previous report.[4]

No abnormalities in appearance or histological examination were observed in either *Angpt2*$^{loxP/–}$ or *Angpt2*$^{loxP/loxP}$ (considered wild-type) mice (Supplemental Figure 18, 19).

Tg(UBC-CreERT2);Angpt2$^{loxP/loxP}$ mice were bred to induce global angiopoietin-2 deletion (Angpt2$^{\Delta UBC}$, Figure 2A). No discernible difference was noted between control and Angpt2$^{\Delta UBC}$ mice two weeks after tamoxifen administration (Supplemental Table 2).

Tg(Pax8-rtTA;LC1);Angpt2$^{loxP/loxP}$ mice were bred to enable tubular epithelial cell (TEC)-specific angiopoietin-2 deletion (Angpt2$^{\Delta TEC}$, Figure 5A). No discernible difference was noted between control and Angpt2$^{\Delta TEC}$ mice two weeks after doxycycline administration (Supplemental Table 3).

Tg(Cdh5-CreERT2);Angpt2$^{loxP/loxP}$ mice were bred to achieve endothelial cell-specific angiopoietin-2 deletion (Angpt2$^{\Delta EC}$, Figure 6A). No discernible difference was noted between control and Angpt2$^{\Delta EC}$ mice two weeks after tamoxifen administration (Supplemental Table 4).

Histology and quantification

Mouse tissues were processed for histological analysis as previously described.[5] Formalin-fixed paraffin-embedded kidneys were sectioned at 5 μm for Periodic Acid-Schiff (PAS) and picrosirius red staining (24901-250, Polysciences, Warrington, PA, USA). Tubulointerstitial injury was assessed on PAS-stained sections using a semiquantitative scoring method. Images of the corticomedullary junction were acquired from 10 random fields per mouse at 200× magnification. Each image was divided into 256 grids to evaluate inflammatory cell infiltration, tubular atrophy or dilation, and cast formation. The percentage of damaged grids was calculated to quantify the degree of injury. Interstitial fibrosis was quantified by measuring picrosirius red^{+} collagen area in the cortex at 200× magnification using ImageJ.

For immunofluorescence staining, tissues embedded in OCT compound (4583,

Sakura Finetek, Torrance, CA, USA) were sectioned at 5 μm. Primary antibodies included F4/80 (1:100, 14-4801-82, eBioscience, San Diego, CA), peroxisomal acyl-coenzyme A oxidase 1 (ACOX1, 1:250, ab184032, Abcam, Cambridge, UK), catalase (1:200, ab52477, Abcam), 70-kDa peroxisomal membrane protein (PMP70, 1:200, ab3321, Abcam), nidogen (1:200, sc-33706, Santa Cruz Biotechnology, Dallas, TX, USA), Tie-2 (1:200, C-20, Santa Cruz Biotechnology), and αSMA-Cy3 (1:200, clone 1A4, Sigma-Aldrich, St. Louis, MO, USA). Fluorophore-conjugated secondary antibody labeling (1:400, Jackson ImmunoResearch, West Grove, PA, USA) , nuclear staining with DAPI (1:1000, Invitrogen), and mounting with VectaShield (H-1000-10, Vector Laboratories, Newark, CA, USA) were conducted as previously described.[6] Imaging was performed using a Zeiss AxioImager M1 microscope equipped with a 40× objective lens and a 10× eyepiece at the Imaging Core Facility, First Core Laboratory, College of Medicine, National Taiwan University. F4/80^{+} cells were defined as cells in which Alexa Fluor 488 fluorescence occupied greater than 75% of the cell area surrounding the DAPI^{+} nuclei. Quantification was expressed as the number of F4/80^{+} cells per high-power field (HPF).

Ex vivo micro-computed tomography (μCT): scanning, reconstruction, and analysis

After euthanizing the mice, cardiac perfusion was performed using 1× PBS containing heparin (10 IU/mL), followed by fixation with 4% paraformaldehyde. Microfil (MV-122, Flow Tech, Carver, MA, USA) was then perfused at a constant rate of 150 mL/hour using a perfusion pump. Renal pedicles were ligated, kidneys were harvested, incubated at 4°C overnight, and subsequently fixed with 10% formalin. Fixed specimens were submitted to the Industrial Technology Research Institute (Hsinchu, Taiwan) for analysis as previously described.[6] The Angpt2$^{\Delta UBC}$ cohort was scanned using a SkyScan 1272 μCT scanner (Bruker micro-CT Systems, Kontich,

Belgium) at 10.0 μm resolution with 180° rotation, 70 kVp voltage, 142 μA current, 10 W output, and a 0.25 mm Al filter. The Angpt2$^{\Delta TEC}$ cohort was scanned using a SkyScan 2211 μCT scanner at 5.0 μm resolution with 180° rotation, 80 kVp voltage, 80 μA current, 1.6 W output, and a 1 mm Al filter.

Image reconstruction with ring-artifact and beam-hardening correction was performed using GPUNRecon software (Bruker micro-CT Systems). Reconstructed datasets were re-aligned and isolated using Dataviewer 1.5.6.2, and three-dimensional visualization was performed with CTVox 3.3.0 (Bruker micro-CT Systems). Regions of interest (ROI; 2-mm thickness, 201 slices) were selected from the lower pole of both kidneys to measure vascularity. Small vessels were defined as those with an inner diameter <40 μm, while large vessels were defined as ≥40 μm. Vascular volume was expressed as the ratio of vascular volume to kidney volume (%). Vascular surface area was normalized to kidney volume, calculated as vascular surface/kidney volume. Vascular density was determined as the structural linear density, representing the linear density of vascular structures.

RNA-Seq of mouse kidneys

Total RNA was extracted from mouse kidneys using TRIzol (15596018, Thermo Fisher Scientific, Grand Island, NY, USA), with one kidney from each mouse forming one sample. The concentration and integrity of total RNA were assessed using a Qubit 3.0 Fluorometer with hsRNA reagent (Thermo Fisher Scientific) and a Qsep100 capillary gel electrophoresis system with RNA R1 cassette (BiOptic, New Taipei City, Taiwan). For cDNA library generation, 1000 ng of total RNA per sample was used with the Illumina Stranded mRNA Prep, Ligation Kit (20040534, Illumina) and IDT for Illumina UD Indexes (20040553, lllumina), following the manufacturer's instructions. The concentration and fragment size of the final libraries were assessed using a Qubit 3.0 Fluorometer with hsDNA reagent and a Qsep100 capillary gel electrophoresis

system with S3 Kilobase cassette. The prepared libraries were sequenced by the Taiwan Genomic Industry Alliance (TGIA, Taipei, Taiwan), using the NovaSeq 6000 platform (Illumina).

Analysis of mouse RNA-Seq data

Raw sequencing reads in FASTQ format underwent quality control (QC) using FastQC (https://www.bioinformatics.babraham.ac.uk/projects/fastqc/). The high-quality reads were then aligned to the mouse reference genome GRCm38 using STAR aligner (v2.7.8a) with the --quantMode parameter, generating gene-level counts based on the GENCODE vM25 gene annotation.[7] Cross-sample normalization was performed using the trimmed mean of M-values (TMM) method, and TPM values were calculated for downstream analysis. To identify enriched biological pathways, gene set enrichment analysis (GSEA) was performed using the R package clusterProfiler (v4.2.2),[8] focusing on Gene Ontology (GO) Biological Process sets obtained from MSigDB (v7.4, C5) (https://www.gsea-msigdb.org/gsea/msigdb/genesets.jsp).[9] PCA was used to visualize global expression patterns and sample clustering, with the normalized expression matrix as the input. The first three principal components (PC1, PC2, and PC3) accounted for the majority of dataset variance. To further explore the biological pathways, IPA (QIAGEN, Hilden, Germany) was used to identify enriched canonical pathways, upstream regulators, and biological functions. Differentially expressed gene (DEG) heatmaps were generated using ClustVis (https://biit.cs.ut.ee/clustvis/).[10] All analyses were conducted in R (v4.1.3).

Plasma collection from mice and biochemical analysis

Whole blood was collected from mice via retrobulbar venous plexus and superior vena cava puncture using heparin-rinsed syringes. The samples were centrifuged at 5400 rpm for 10 minutes at 4°C to obtain plasma. Biochemical analyses of blood urea nitrogen (BUN), creatinine, glucose, and triglyceride levels were performed using the

Roche cobas® c111 automatic biochemical analyzer (Roche Diagnostics, Indianapolis, IN) in the Laboratory Animal Center, National Taiwan University College of Medicine.

Isolation of endothelial cells from kidneys

After euthanizing the mice, kidneys were collected and dissociated into single-cell suspensions using the Multi Tissue Dissociation Kit 2 (130-110-203, Miltenyi Biotec, San José, CA) in combination with C Tubes (130-093-237) on the gentleMACS Octo Dissociator with Heaters (130-096-427), following the manufacturer's protocol. The suspension was filtered through a 40 μm cell strainer, centrifuged, and then enriched for endothelial cells using CD31 magnetic MicroBeads (130-097-418) and an MS column (130-042-201) with autoMACS® Running Buffer – MACS® Separation Buffer (130-091-221) on an OctoMACS® Separator (130-042-109). For fluorescence-activated cell sorting (FACS), cells were stained with DAPI to exclude dead cells, anti-platelet-derived growth factor receptor β-APC (anti-PDGFRβ-APC, 17-1402, eBioscience) to exclude pericytes, anti-E-Cadherin-APC (147312, BioLegend, San Diego, CA) to exclude epithelial cells, and anti-CD31-PE (102408, BioLegend) to label endothelial cells. Endothelial cells were further isolated via FACSAria cell sorting (BD Biosciences, San José, CA) by selecting $CD31^{+}DAPI^{-}PDGFR\beta^{-}E\text{-}Cadherin^{-}$ cells. The isolated endothelial cells were used for quantitative PCR and western blot analysis.

Isolation of macrophages from kidneys

Kidneys were dissociated into single-cell suspensions using the Multi Tissue Dissociation Kit 2, following previously described protocols. The resulting cells were processed for FACS by staining with DAPI (to exclude dead cells), anti-Ly6G-APC (127613, BioLegend), anti-NK1.1-APC (108710, BioLegend), anti-vascular endothelial growth factor receptor 2-APC (anti-VEGFR2-APC, 136406, BioLegend), and anti-CD11b-PE (101208, BioLegend). Macrophages were sorted as $CD11b^{high}DAPI^{-}Ly6G^{-}NK1.1^{-}VEGFR2^{-}$ cells using the FACSAria cell sorter (BD

Biosciences). The isolated macrophages were then used for quantitative PCR analysis.

In vitro experiments of primary tubular epithelial cells

Six-well culture plates were pre-coated with Attachment Factor (S006100, Thermo Fisher Scientific) for 30 minutes, and primary tubular epithelial cells (PTECs) were seeded at a density of 1×10^5 cells per well. When cells reached approximately 90% confluence, they were serum-deprived in medium containing 0.1% fetal bovine serum for 24 hours to synchronize cellular responses. Subsequently, cells were treated with recombinant mouse SCF (negative control; 455-MC, R&D Systems, Minneapolis, MN, USA), recombinant angiopoietin-2 (100 or 200 ng/mL; 7186-AN, R&D Systems), or tumor necrosis factor-α (TNF-α, 0.08 ng/mL; 410-MT, R&D Systems). After 24 hours of stimulation, total RNA and protein lysates were collected for downstream quantitative PCR and Western blot analyses.

Protein extraction and western blot analysis

Proteins were extracted from kidneys or cells using radioimmunoprecipitation assay (RIPA) buffer (20-188, Millipore, Burlington, MA, USA) supplemented with cOmplete™ Protease Inhibitor Cocktail (04693132001, Roche, Mannheim, Germany) and PhosSTOP (04906837001, Roche). Lysates were agitated at 4°C for 30 minutes. Whole-kidney lysates 100 μg protein, endothelial cell lysates 50 μg protein, and PTEC lysates 30 μg protein were normalized. Samples were denatured in Laemmli Sample Buffer (1610747, Bio-Rad, Hercules, CA, USA) containing 2-mercaptoethanol (M74154, Sigma-Aldrich), separated by SDS-polyacrylamide gel electrophoresis, and transferred to polyvinylidene fluoride membrane (Millipore). The following primary antibodies were used to detect proteins: vascular cell adhesion molecule 1 (VCAM1, 1:2000, ab134047, Abcam), C-X3-C motif chemokine ligand 1 (CX3CL1, 1:1000, 14-7986-81, Invitrogen), C-C motif chemokine ligand 2 (CCL2, 1:2000, ab25124, Abcam), intercellular adhesion molecule 1 (ICAM1, 1:2000, AF796, R&D Systems), Phospho-

Tyrosine (P-Tyr-102, 1:1000, 9416T, Cell Signaling Technology, Danvers, MA), Tie-2 (1:2000, AF762, R&D Systems), Integrin α5 (1:1000, ab1928, Abcam), Integrin β1 (1:2000, AF762, R&D Systems), β-actin (1:1000, 4967, Cell Signaling Technology), glyceraldehyde-3-phosphate dehydrogenase (GAPDH, 1:2500, ab9485, Abcam), ACOX1 (1:1000, ab184032, Abcam), catalase (1:1000, ab52477, Abcam), and PMP70 (1:500, ab3421, Abcam). Total protein normalization for ACOX1, catalase, and PMP70 was additional performed using Ponceau S staining. The five most intense bands per lane were quantified, and their mean intensity was used as the normalization factor for protein loading (Supplemental Figure 3).

Enzyme-linked immunosorbent assay

Mouse kidney proteins were extracted using RIPA lysis buffer with cOmplete™ Protease Inhibitor Cocktail for enzyme-linked immunosorbent assay (ELISA). The total protein concentration in the supernatant after centrifugation was quantified using a total protein assay. Angiopoietin-2 was measured in duplicate using a commercial mouse angiopoietin-2 ELISA kit (MANG20, R&D Systems). The protein concentration in the kidneys was corrected by the total protein concentration and expressed as pg/mg.

Quantitative polymerase chain reaction

Total RNA was extracted from mouse kidneys using TRIzol (Thermo Fisher Scientific) and from cells using the RNeasy Plus Mini Kit (74136, QIAGEN). RNA concentration and purity were assessed using the A260/280 ratio. The extracted RNA was reverse-transcribed into complementary DNA (cDNA) using the iScript cDNA Synthesis Kit (1708891, Bio-Rad). Quantitative PCR was performed with iQ™ SYBR® Green Supermix (1708882, Bio-Rad) on the CFX96 real-time PCR system (Bio-Rad). The specific primer sequences used are listed in Supplemental Table 6.

Genotyping by polymerase chain reaction

A small section of the tail from transgenic mice was collected, and genomic DNA

was extracted using tail lysis buffer and proteinase K (V3021, Promega, Madison, WI, USA).[6] PCR was performed for genotyping using a thermal cycler (T100, Bio-Rad). The specific primers utilized for genotyping are provided in Supplemental Table 7.

Statistics (The ARRIVE guidelines 2.0 Essential 10)

Sample size determination followed the ARRIVE 2.0 sample size items (2a, 2b). Sample sizes for each experiment were based on pilot data from our laboratory and previously published studies using similar kidney injury models. These sources provided estimates of the expected effect size and variability for the primary outcomes, allowing selection of the minimum number of animals required to detect biologically meaningful differences while adhering to the principles of reduction in animal research. For each experiment, the selected sample size provided approximately 80% statistical power at an alpha level of 0.05 based on a two-sample, two-tailed design (calculated using PrismCloud). Final sample sizes were consistent with those commonly used in comparable mechanistic or therapeutic studies. Exact n values for each analysis are reported in the corresponding figure legends.

For experiments involving animals and in vitro models, all analyses were pre-specified at the level of the experimental unit (mouse or cell). Distributional assumptions were assessed using the Shapiro-Wilk test (primary), complemented by Anderson-Darling, D'Agostino-Pearson, and Kolmogorov-Smirnov tests and by Q-Q plots, following the ARRIVE guidelines 2.0 for transparent reporting of statistical assumptions. For datasets with sample size <8, group comparisons were performed using the two-tailed Mann-Whiney U test with Hodges-Lehmann effect size and 95% confidence interval (CI), or by Kruskal-Wallis test with Dunn's multiple comparisons. This approach mitigates the limited power and potential bias of normality tests in small-sample settings. For other datasets, normality was evaluated as above. When raw values showed evidence of non-normality, log transformation was applied, and data were

analyzed using an unpaired t-test with Welch's correction (two-tailed). A Mann-Whitney sensitivity analysis was additionally performed to confirm the robustness of interferences across statistical methods. All experimental statistical analyses were conducted using GraphPad Prism version 10.4.1 (GraphPad Software, San Diego, CA, USA).

References

1. Tsai MT, Ou SM, Lee KH, Lin CC, Li SY. Circulating Activin A, Kidney Fibrosis, and Adverse Events. *Clin J Am Soc Nephrol*. Feb 1 2024;19(2):169-177. doi:10.2215/CJN.0000000000000365

2. Tsai YC, Hsieh TH, Liao YR, et al. METTL3-Mediated N 6 -Methyladenosine mRNA Modification and cGAS-STING Pathway Activity in Kidney Fibrosis. *J Am Soc Nephrol*. Oct 1 2024;35(10):1312-1329. doi:10.1681/ASN.0000000000000428

3. Chen CY, Tsai MS, Lin CY, et al. Rescue of the genetically engineered Cul4b mutant mouse as a potential model for human X-linked mental retardation. *Hum Mol Genet*. Oct 1 2012;21(19):4270-4285. doi:10.1093/hmg/dds261

4. Gale NW, Thurston G, Hackett SF, et al. Angiopoietin-2 is required for postnatal angiogenesis and lymphatic patterning, and only the latter role is rescued by Angiopoietin-1. *Dev Cell*. Sep 2002;3(3):411-423. doi:10.1016/s1534-5807(02)00217-4

5. Chang FC, Chiang WC, Tsai MH, et al. Angiopoietin-2-induced arterial stiffness in CKD. *J Am Soc Nephrol*. Jun 2014;25(6):1198-1209. doi:10.1681/ASN.2013050542

6. Chang FC, Liu CH, Luo AJ, et al. Angiopoietin-2 inhibition attenuates kidney fibrosis by hindering chemokine C-C motif ligand 2 expression and apoptosis of endothelial cells. *Kidney Int*. Oct 2022;102(4):780-797. doi:10.1016/j.kint.2022.06.026

7. Dobin A, Davis CA, Schlesinger F, et al. STAR: ultrafast universal RNA-seq aligner. *Bioinformatics*. Jan 1 2013;29(1):15-21. doi:10.1093/bioinformatics/bts635

8. Yu G, Wang LG, Han Y, He QY. clusterProfiler: an R package for comparing biological themes among gene clusters. *OMICS*. May 2012;16(5):284-287. doi:10.1089/omi.2011.0118

9. Subramanian A, Tamayo P, Mootha VK, et al. Gene set enrichment analysis: a knowledge-based approach for interpreting genome-wide expression profiles. *Proc Natl Acad Sci U S A*. Oct 25 2005;102(43):15545-15550. doi:10.1073/pnas.0506580102

10. Metsalu T, Vilo J. ClustVis: a web tool for visualizing clustering of multivariate data using Principal Component Analysis and heatmap. *Nucleic Acids Res*. Jul 1 2015;43(W1):W566-570. doi:10.1093/nar/gkv468

Supplemental Figures

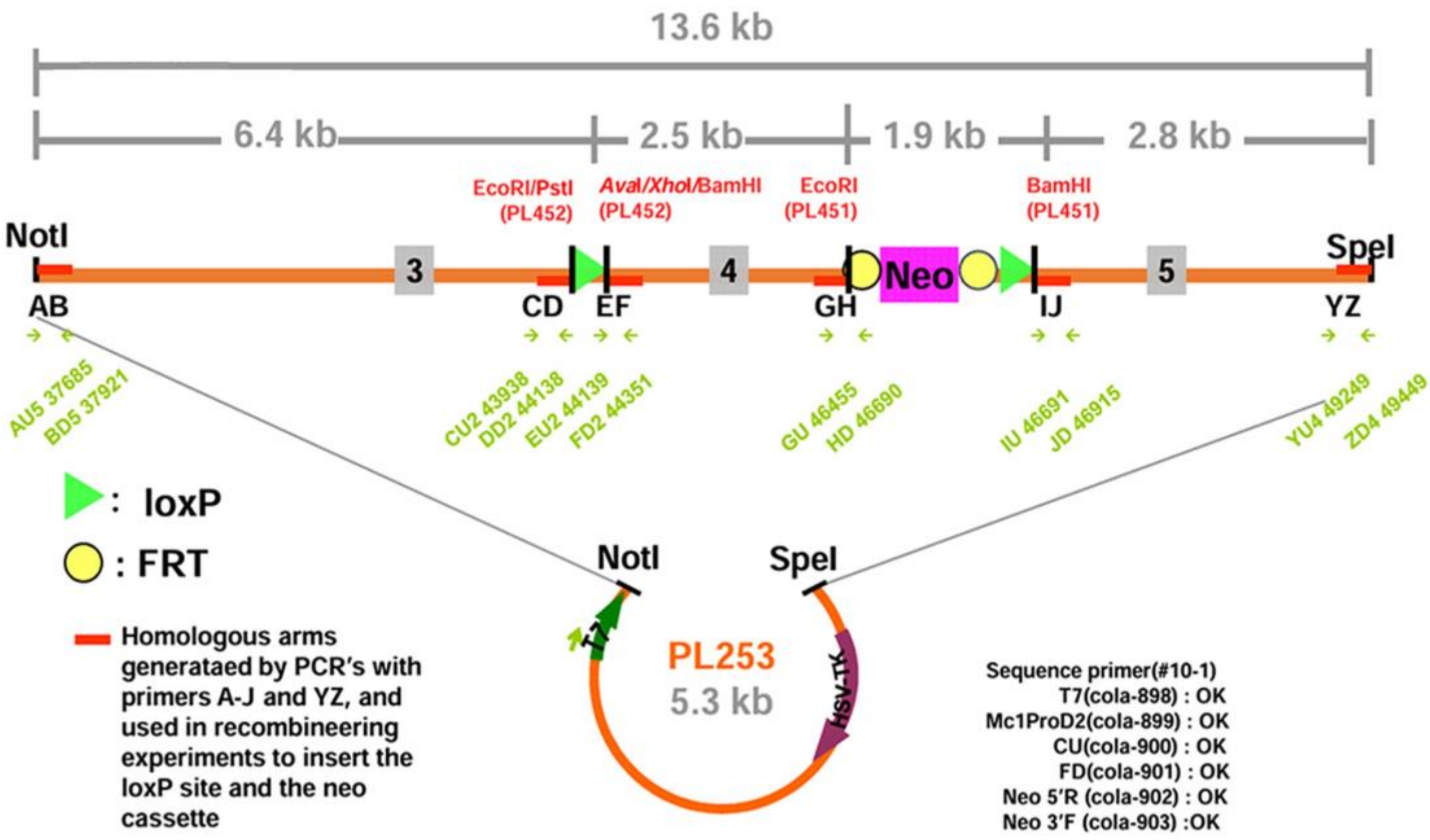

Supplemental Figure 1. Targeting vector for generating *Angpt2*$^{loxP/loxP}$ mice. The targeting vector was constructed containing exon 4 of the mouse *Angpt2* gene, with a neomycin resistance cassette (Neo cassette) flanked by loxP and FRT sites, respectively.

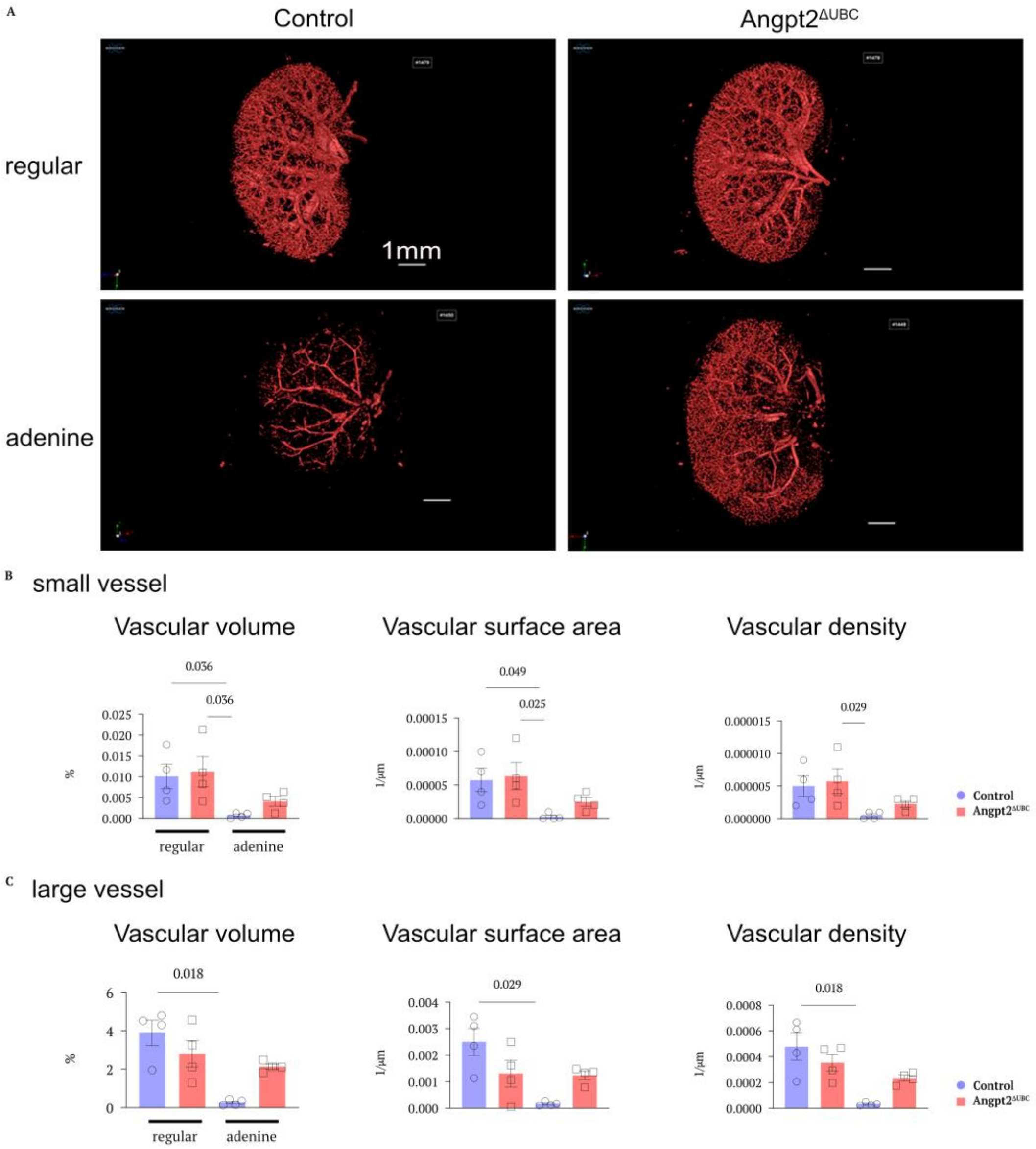

Supplemental Figure 2. Global angiopoietin-2 deletion preserves vascular integrity in adenine-induced chronic kidney disease. (**A**) Representative *ex vivo* µCT images showing 3-dimensional reconstructions of Microfil-perfused kidneys from Control and Angpt2$^{\Delta UBC}$ mice 4 weeks after adenine-induced chronic kidney disease (CKD). Angpt2$^{\Delta UBC}$ refers to mice with tamoxifen-induced global deletion of angiopoietin-2, while Control mice lack Cre expression. Scale bars: 1 mm. (**B**, **C**) Bar graphs quantifying vascular volume, vascular surface area, and vascular density based on µCT analysis. Vessels are classified as small (< 40 µm in diameter) in **B** or large (≥

40 μm in diameter) in **C**. Data are expressed as means ± SEM, with each dot representing an individual mouse. Statistical significance was determined using Kruskal-Wallis test with Dunn's multiple comparisons. $n = 4$ mice per group.

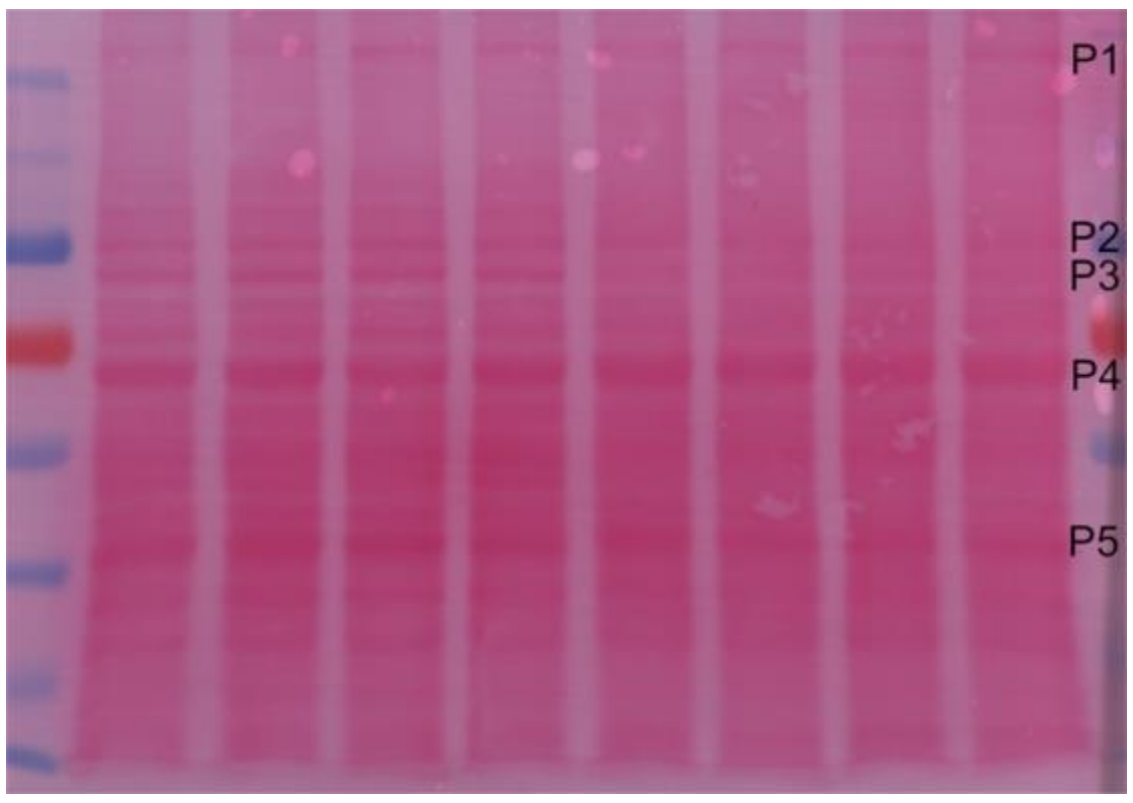

Supplemental Figure 3. Ponceau S total protein staining and lane-specific loading factors. Representative membrane stained with Ponceau S. For each lane, the five most intense bands (P1–P5) were quantified, and their mean intensity was used as the loading (normalization) factor for subsequent Western blot quantification (ACOX1, catalase, PMP70).

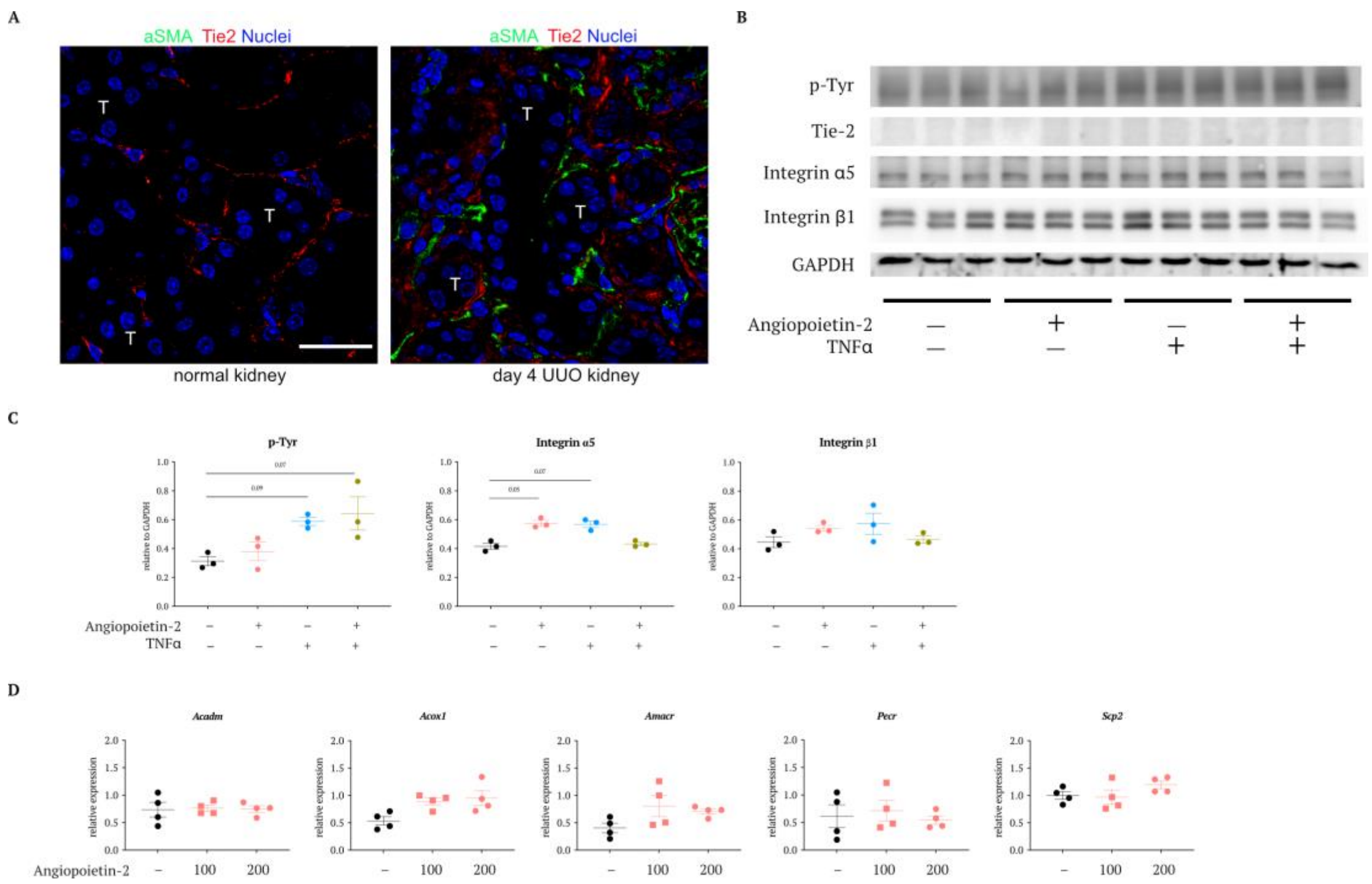

Supplemental Figure 4. Angiopoietin-2 does not induce downstream signaling or alter metabolic gene expression in primary tubular epithelial cells. (**A**) Representative images of Tie-2-expressing cells in kidney sections. Nuclei were counterstained with 4′,6-diamidino-2-phenylindole (DAPI). Scale bar: 50 µm (original magnification 400×). (**B**) Representative Western blot analysis of phosphotyrosine (pTyr), Tie-2, integrin α5, integrin β1, and GAPDH in primary tubular epithelial cells (PTECs) treated with angiopoietin-2 (200 ng/mL) and TNFα (0.08 ng/mL). (**C**) Bar graphs showing the relative protein expression levels of pTyr, integrin α5, and integrin β1, normalized to GAPDH. (**D**) Bar graphs showing the relative expression of genes involved in peroxisomal β-oxidation, normalized to *Gapdh*, in PTECs treated with or without angiopoietin-2 (100, 200 ng/mL). Data are expressed as means ± SEM, with each dot representing an independent experiment. Statistical significance was determined using Kruskal-Wallis test with Dunn's multiple comparisons. $n = 3$ in **C** and $n = 4$ in **D** per condition.

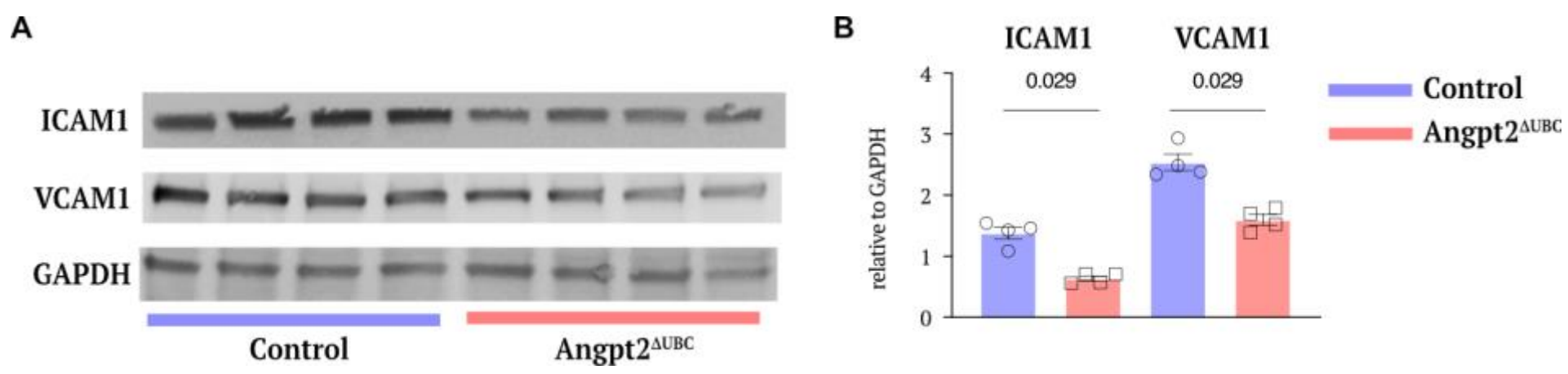

Supplemental Figure 5. Global angiopoietin-2 deletion downregulates adhesion molecules in advanced adenine-CKD. (**A**) Representative Western blot analysis showing ICAM1, VCAM1, and GAPDH expression in kidney tissues from Control and Angpt2$^{\Delta UBC}$ mice 3 weeks after adenine-induced CKD. (**B**) Bar graphs showing the relative ICAM1 and VCAM1 protein expression, normalized to GAPDH. Data are expressed as means ± SEM, with each dot representing an individual mouse. Statistical significance was determined using two-tailed Mann-Whitney U test. *n* = 4 mice per group.

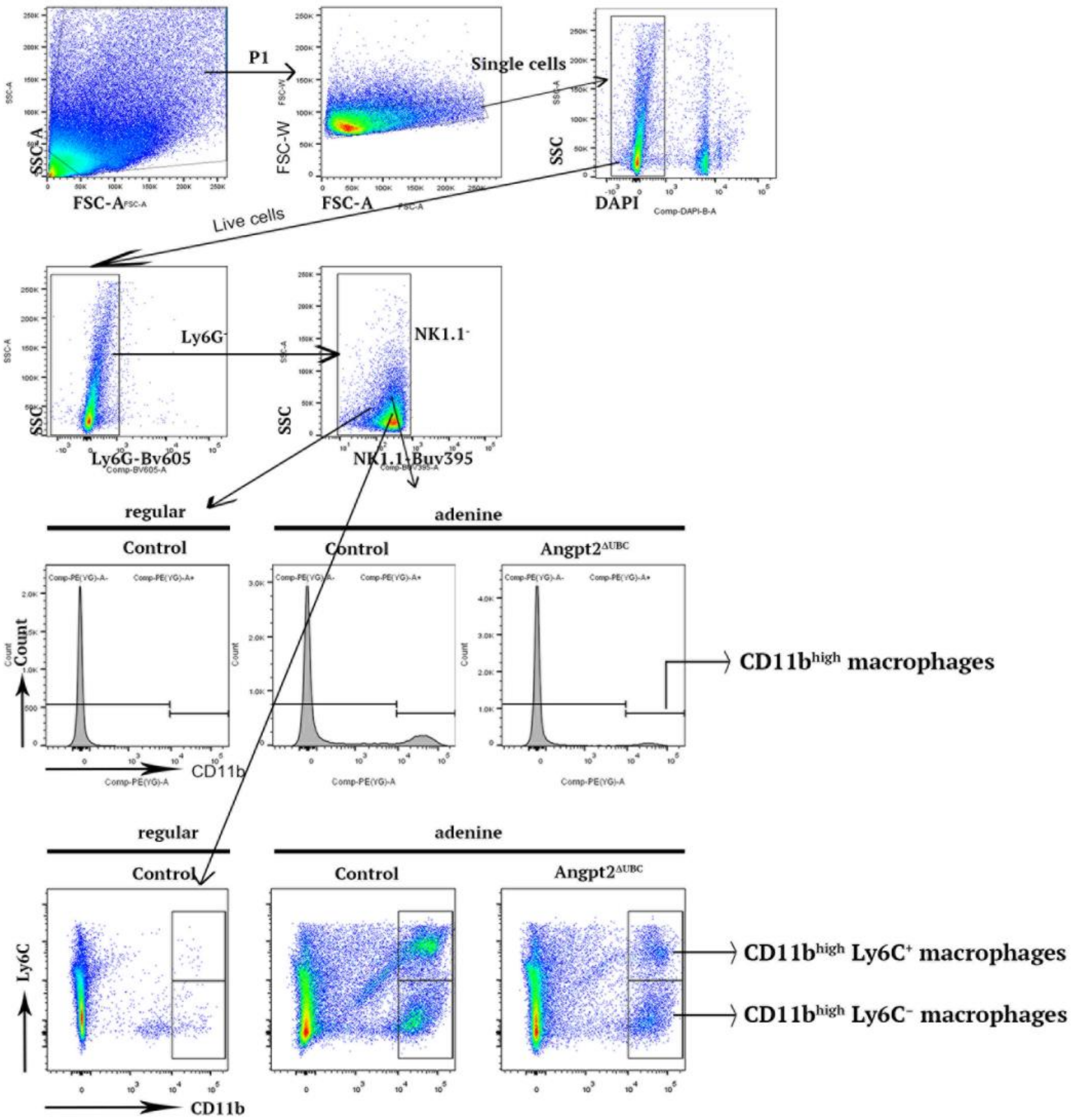

Supplemental Figure 6. Gating strategy for kidney macrophage analysis by flow cytometry. Representative plots showing FACS analysis of macrophage populations in kidney tissues from Control and Angpt2$^{\Delta UBC}$ mice 3 weeks after adenine-induced CKD. Control mice on a regular chow diet served as the normal control. Single cells were identified using FSC-A and FSC-W, and DAPI staining was used to exclude dead cells. Kidney macrophage subpopulations were characterized based on CD11b^{high} (Ly6G^{-}NK1.1^{-}) and Ly6C expression.

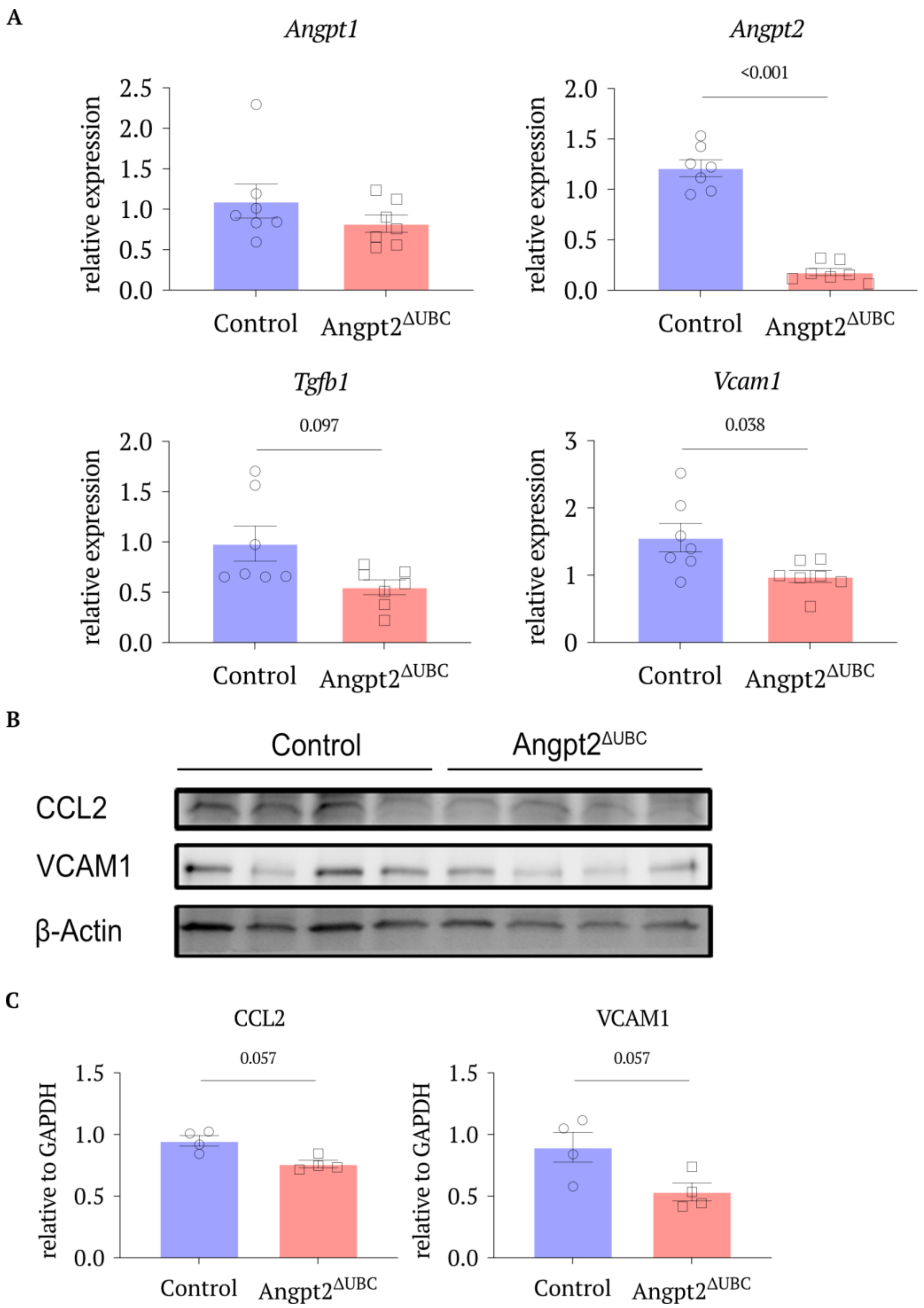

Supplemental Figure 7. Global deletion of angiopoietin-2 reduces pro-inflammatory and pro-fibrotic factors in endothelial cells in advanced adenine-induced CKD. (**A**) Bar graphs showing the relative expression of genes encoding angiopoietins, transforming growth factor-β1, and adhesion molecules, normalized to *Gapdh*, in $CD31^{+}$ endothelial cells isolated from the kidneys of Control and

Angpt2$^{\Delta UBC}$ mice 3 weeks after adenine-induced CKD. (**B**, **C**) Western blot analysis of CCL2, VCAM1, and β-actin in CD31^{+} endothelial cells isolated from the kidneys of Control and Angpt2$^{\Delta UBC}$ mice 3 weeks after adenine-induced CKD in **B**. Corresponding bar graphs showing relative protein expression levels of CCL2 and VCAM1, normalized to β-actin in **C**. Data are expressed as means ± SEM, with each dot representing an individual mouse. Statistical significance was determined using two-tailed Mann-Whitney U test.. *n* = 7 mice per group in **A**, and *n* = 4 mice per group in **B**, **C**.

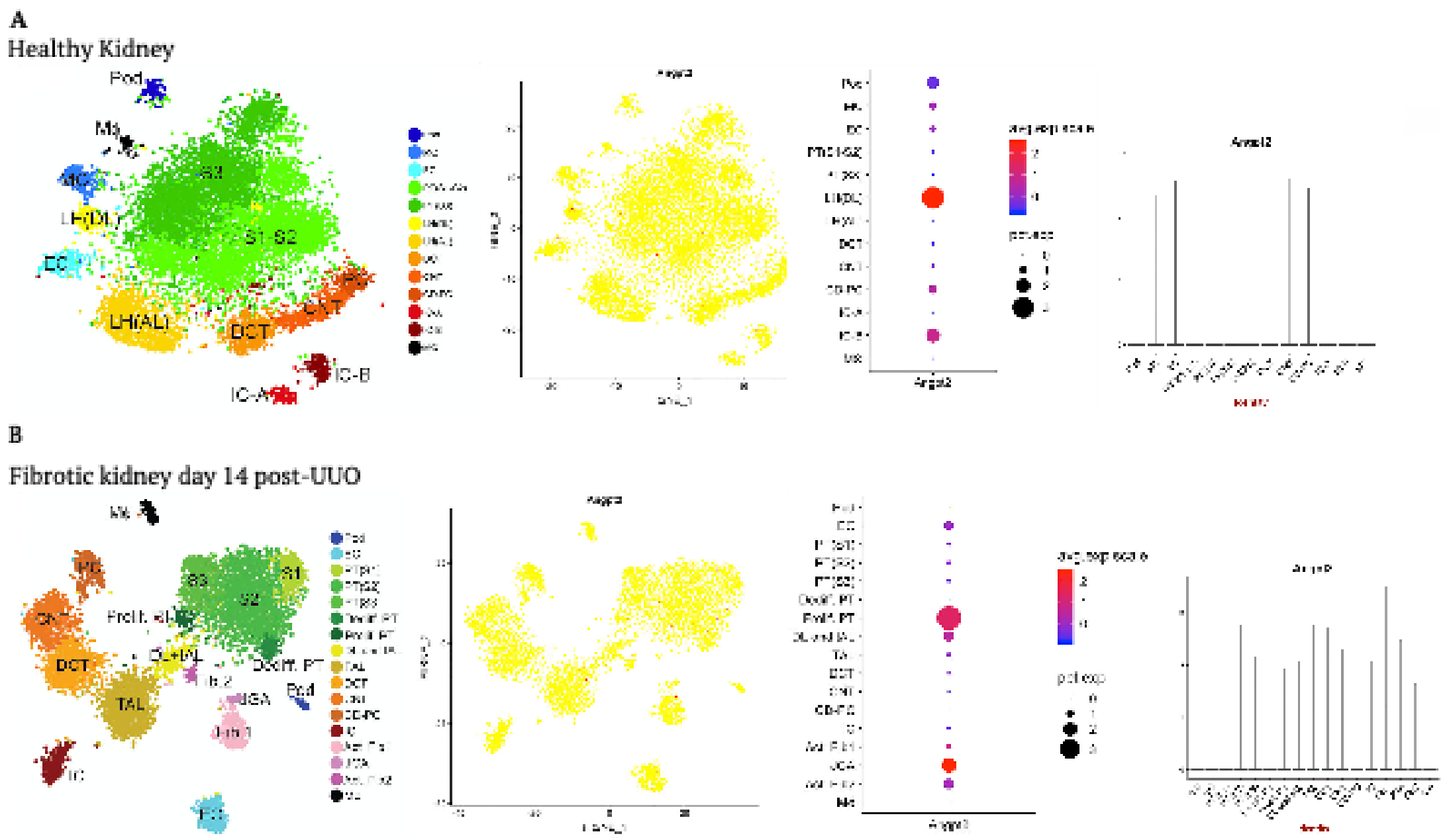

Supplemental Figure 8. ***Angpt2*** **expression in healthy and fibrotic mouse kidneys.** *Angpt2* expression is visualized per condition as cell-type t-SNE, feature plot (per-cell signal, sensitive to dropout/thresholding), dot plot (percent expressing and average among positives), and violin plot (within-cluster distribution) (from left to right). The t-SNE analysis identified 13 distinct cell clusters from a total of 11,395 cells/nuclei in healthy kidneys (**A**). In fibrotic kidneys (14 days after unilateral ureteral obstruction surgery), t-SNE analysis identified 17 distinct cell clusters from 6,147 nuclei (**B**). Abbreviations included Act. (activated), CD-PC (collecting duct-principal cell), CNT (connecting tubule), DCT (distal convoluted tubule), Dediff. (dedifferentiated), DL and tAL (descending limb and thin ascending limb), EC (endothelial cell), Fib. (fibroblast), IC (intercalated cell), IC-A (intercalated cell type A), IC-B (intercalated cell type B), JGA (juxtaglomerular apparatus), LH(AL) (loop of Henle ascending limb), LH(DL) (loop of Henle descending limb), MΦ (macrophage), MC (mesangial cell), PC (principal cell), Pod (podocyte), Prolif. (proliferating), PT (proximal tubule), TAL (thick ascending limb), and UUO (unilateral ureteral obstruction). Data were retrieved from http://humphreyslab.com/SingleCell/ (accessed on October 9, 2024).

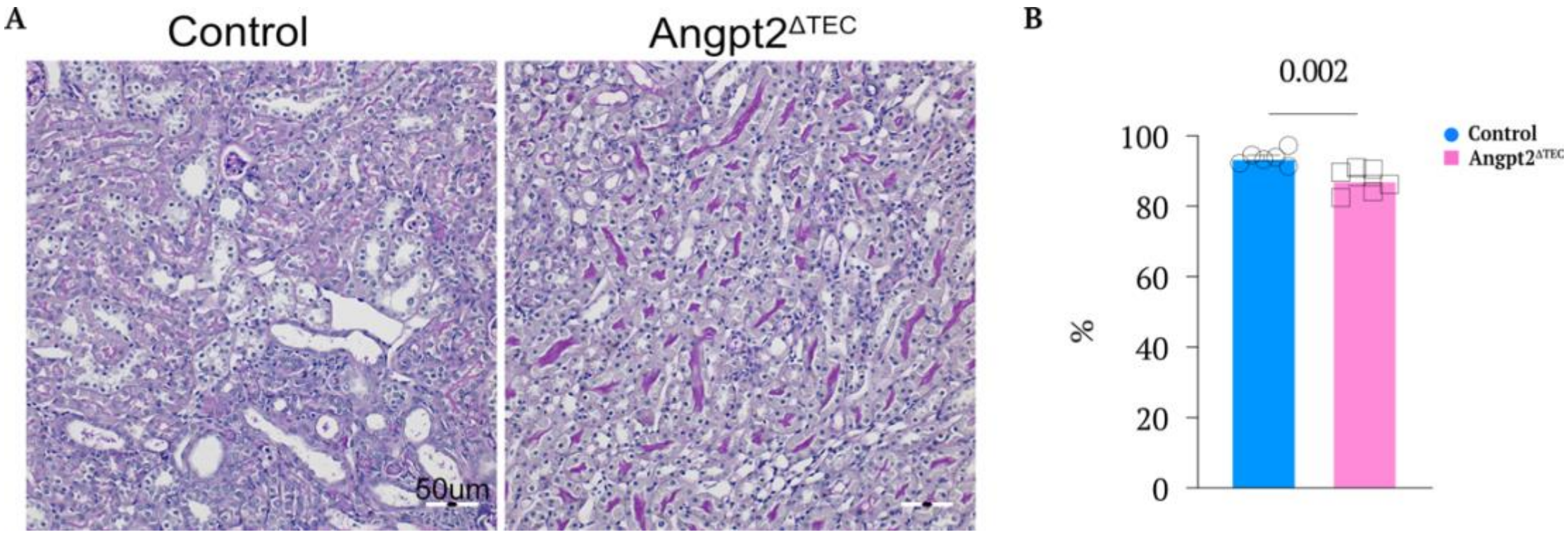

Supplemental Figure 9. Tubular epithelial cell-specific angiopoietin-2 deletion reduces tubulointerstitial injury in advanced adenine-induced CKD. (**A**) Representative images of PAS-stained kidney sections from Angpt2$^{\Delta TEC}$ and Control mice. Angpt2$^{\Delta TEC}$ refers to mice with doxycycline-induced TEC-specific deletion of angiopoietin-2, while Control mice lack Cre expression. Advanced CKD was induced as described in Figure 5A, and kidney sections were analyzed 3 weeks after adenine-induced CKD. Scale bars: 50 μm (original magnification 200×). (**B**) Bar graph quantifying tubulointerstitial injury scores from PAS-stained kidney sections at 200× magnification. Data are expressed as means ± SEM, with each dot representing an individual mouse. Statistical significance was determined using two-tailed Mann-Whitney U test. *n* = 6 mice per group.

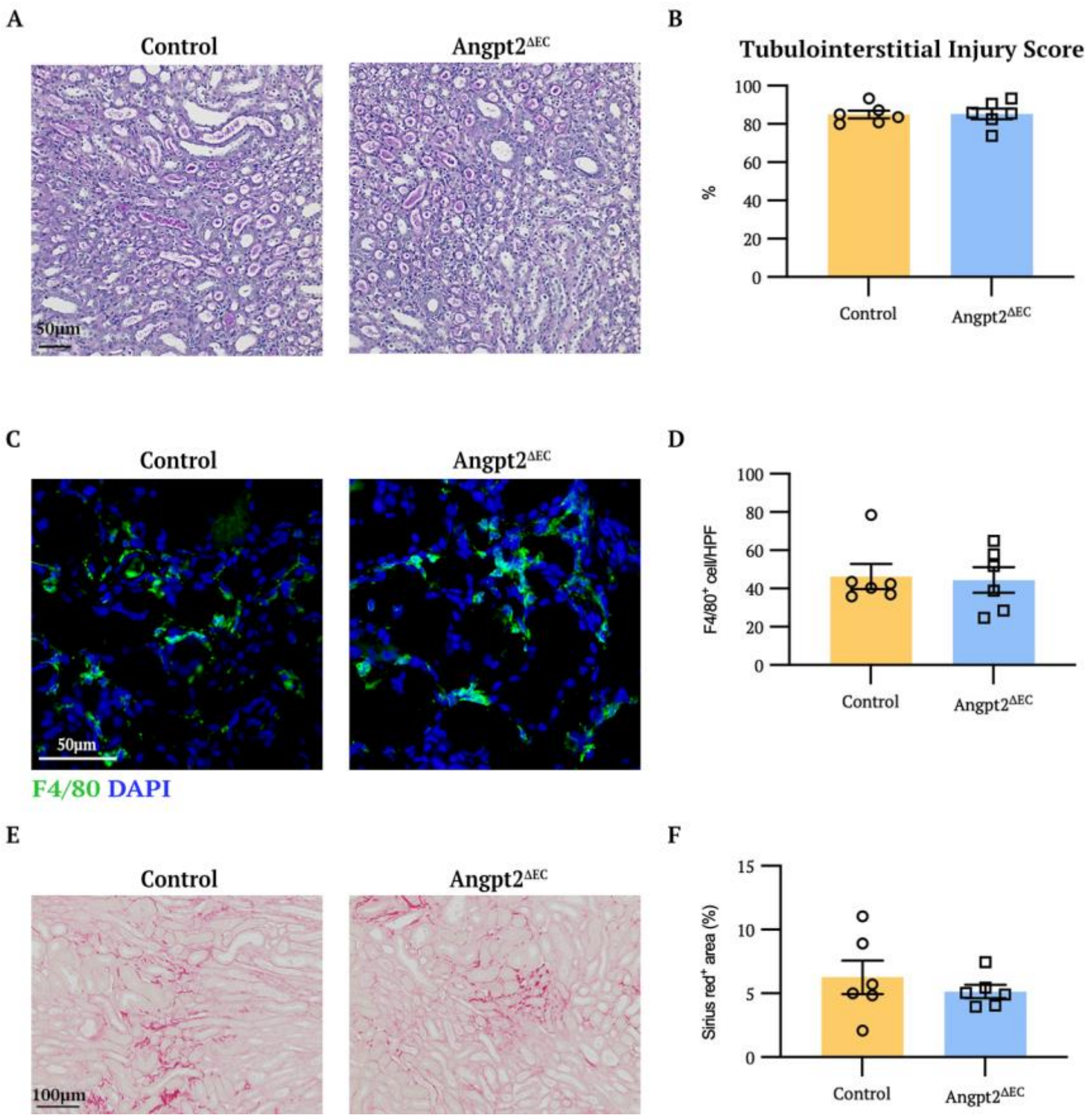

Supplemental Figure 10. Endothelial cell-specific angiopoietin-2 deletion does not attenuate tubulointerstitial injury, macrophage infiltration, or kidney fibrosis in advanced adenine-induced CKD. (**A**) Representative images of PAS-stained kidney sections from Angpt2$^{\Delta EC}$ and Control mice. Angpt2$^{\Delta EC}$ refers to mice with tamoxifen-induced endothelial cell-specific deletion of angiopoietin-2, while Control mice lack Cre expression. Advanced CKD was induced as described in Figure 6B, and kidney sections were analyzed 3 weeks after adenine-induced CKD. Scale bars: 50 μm (original magnification 200×). (**B**) Bar graph showing tubulointerstitial injury scores quantified from PAS-stained kidney sections at 200× magnification. (**C**) Representative images of F4/80^{+} macrophages in kidney sections. Nuclei were counterstained with DAPI. Scale bar: 50 μm (original magnification 400×). (**D**) Bar graph depicting the number of F4/80^{+} macrophages per HPF at 400× magnification.

(**E**) Representative images of picrosirius red-stained fibrillar collagen in kidney sections. Scale bars: 100 µm (original magnification 200×). (**F**) Bar graph showing morphometric quantification of picrosirius red-stained areas in kidney sections at 200× magnification. Data are expressed as means ± SEM, with each dot representing an individual mouse. Statistical significance was determined using two-tailed Mann-Whitney U test. $n = 6$ mice per group.

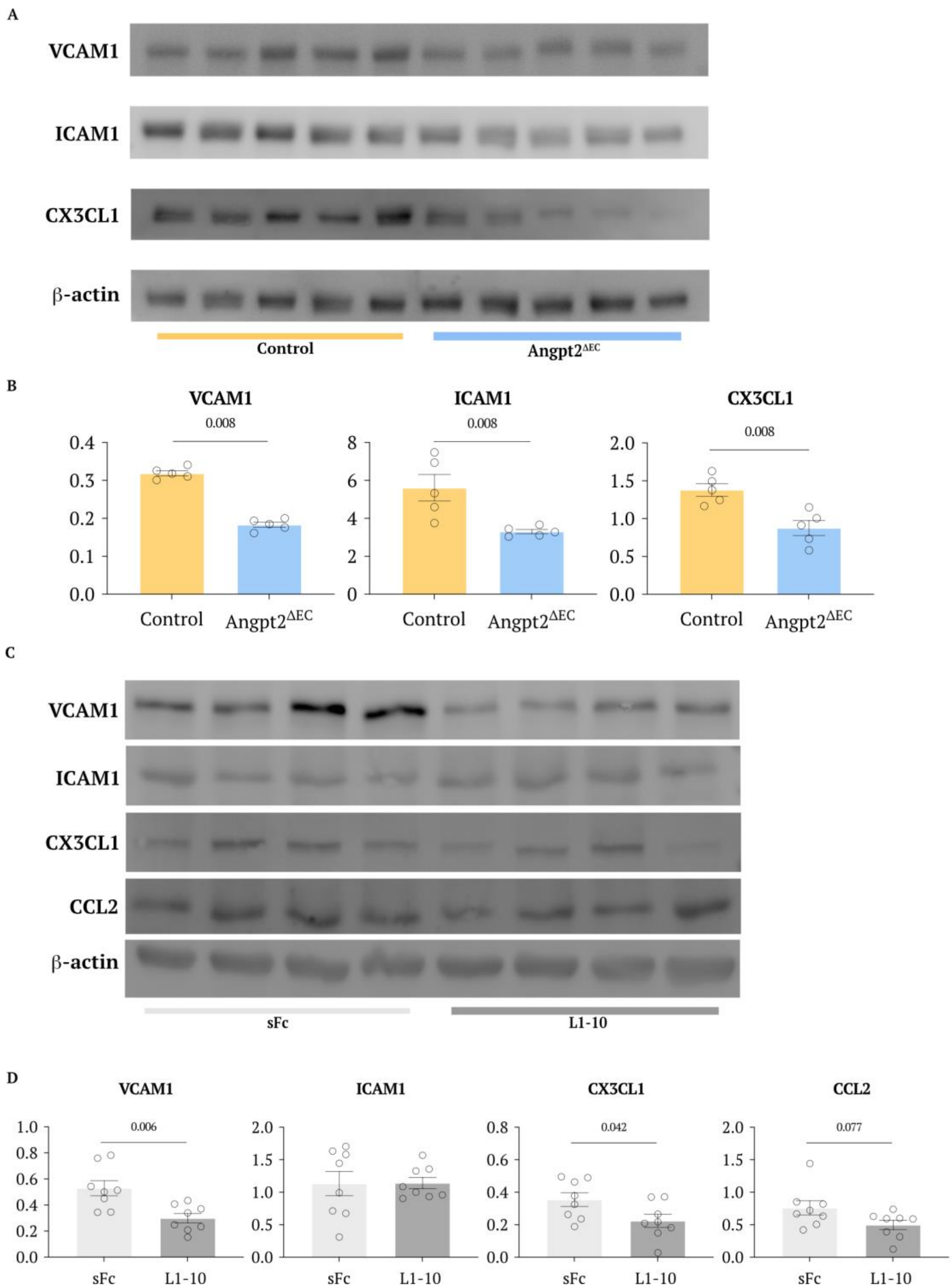

Supplemental Figure 11. Endothelial cell-specific angiopoietin-2 deletion reduces pro-inflammatory and pro-adhesive molecule expression in endothelial cells during early adenine-induced CKD. (**A**, **B**) Western blot analysis of VCAM1, ICAM1, CX3CL1, and β-actin in $CD31^+$ endothelial cells isolated from the kidneys of

Control and Angpt2$^{\Delta EC}$ mice in Figure 6E (**A**). Corresponding bar graphs showing the relative expression levels of VCAM1, ICAM1, and CX3CL1, normalized to β-actin (**B**). (**C**, **D**) Western blot analysis of VCAM1, ICAM1, CX3CL1, CCL2, and β-actin in CD31$^+$ endothelial cells isolated from kidneys of mice treated with sFc and L1-10 for 7 days in Figure 6H (**C**). Corresponding bar graphs showing the relative expression levels of VCAM1, ICAM1, CX3CL1, and CCL2, normalized to β-actin (**D**). Data are expressed as means ± SEM, with each dot representing an individual mouse. Statistical significance was determined using two-tailed Mann-Whitney U test. n = 5 mice per group in **A**, **B** and n = 8 mice per group in **C**, **D**.

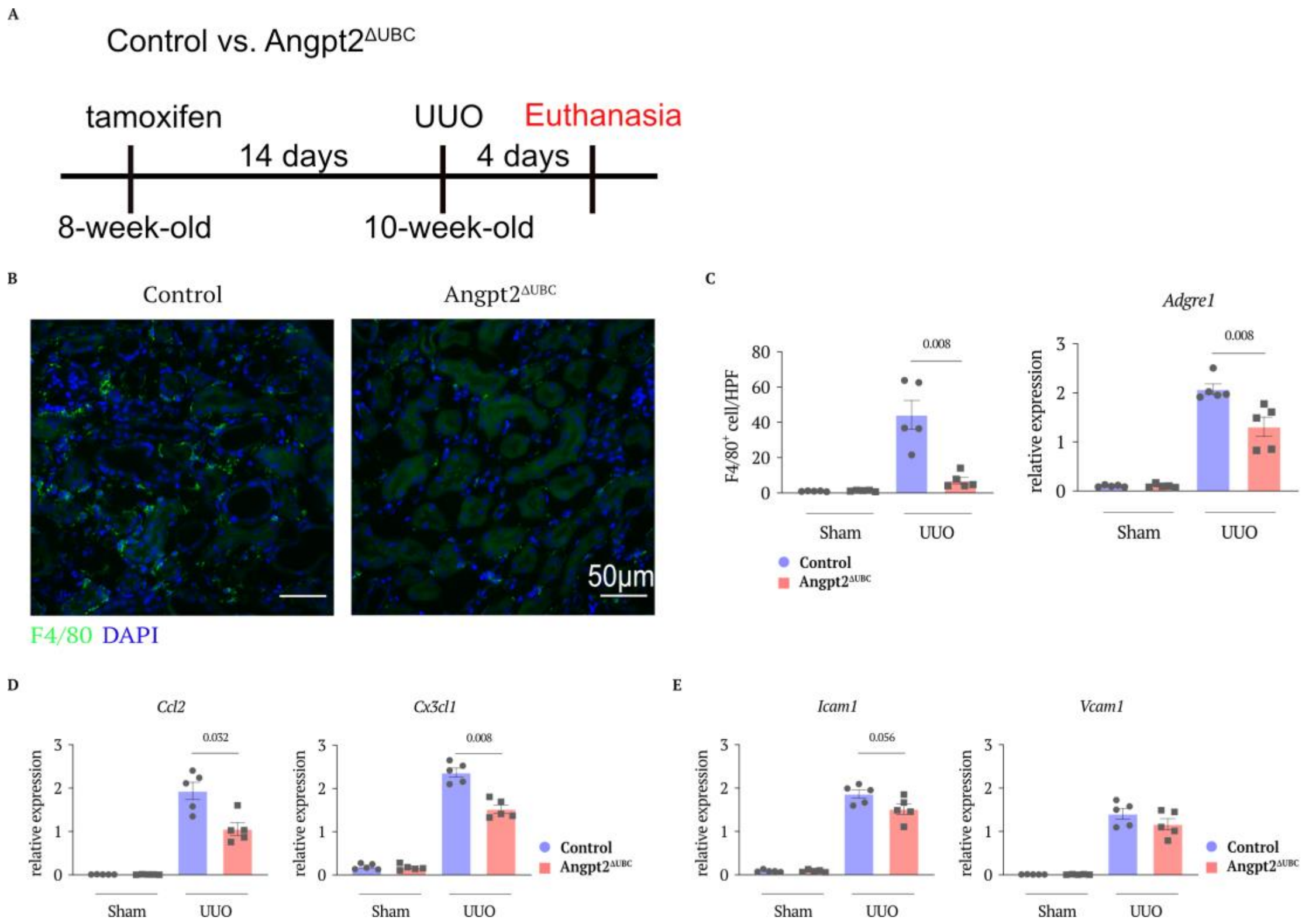

Supplemental Figure 12. Global angiopoietin-2 deletion attenuates inflammatory phenotype in early UUO-induced CKD. (**A**) Schematic diagram illustrating the experimental protocol for tamoxifen administration and UUO-induced kidney fibrosis in control and Angpt2$^{\Delta UBC}$ mice. Littermates lacking Cre serviced as controls (Control), while sham-operated mice served as healthy controls (Sham). Mice were analyzed on day 4 after the surgery. (**B**, **C**) Representative images showing F4/80^{+} macrophages in kidney sections from UUO-operated mice (**B**). Nuclei were counterstained with DAPI. Scale bar: 50 µm, original magnification 400×. Bar graph indicating the number of F4/80^{+} macrophages per HPF at 400× magnification (**C**). (**D**, **E**) Bar graphs showing the relative expression levels of pro-inflammatory genes (**D**) and adhesion molecule-encoding genes (**E**), normalized to *Gapdh*. Data are expressed as means ± SEM, with each dot representing an individual mouse. Statistical significance was determined using two-tailed Mann-Whitney U test. *n* = 5 mice per group.

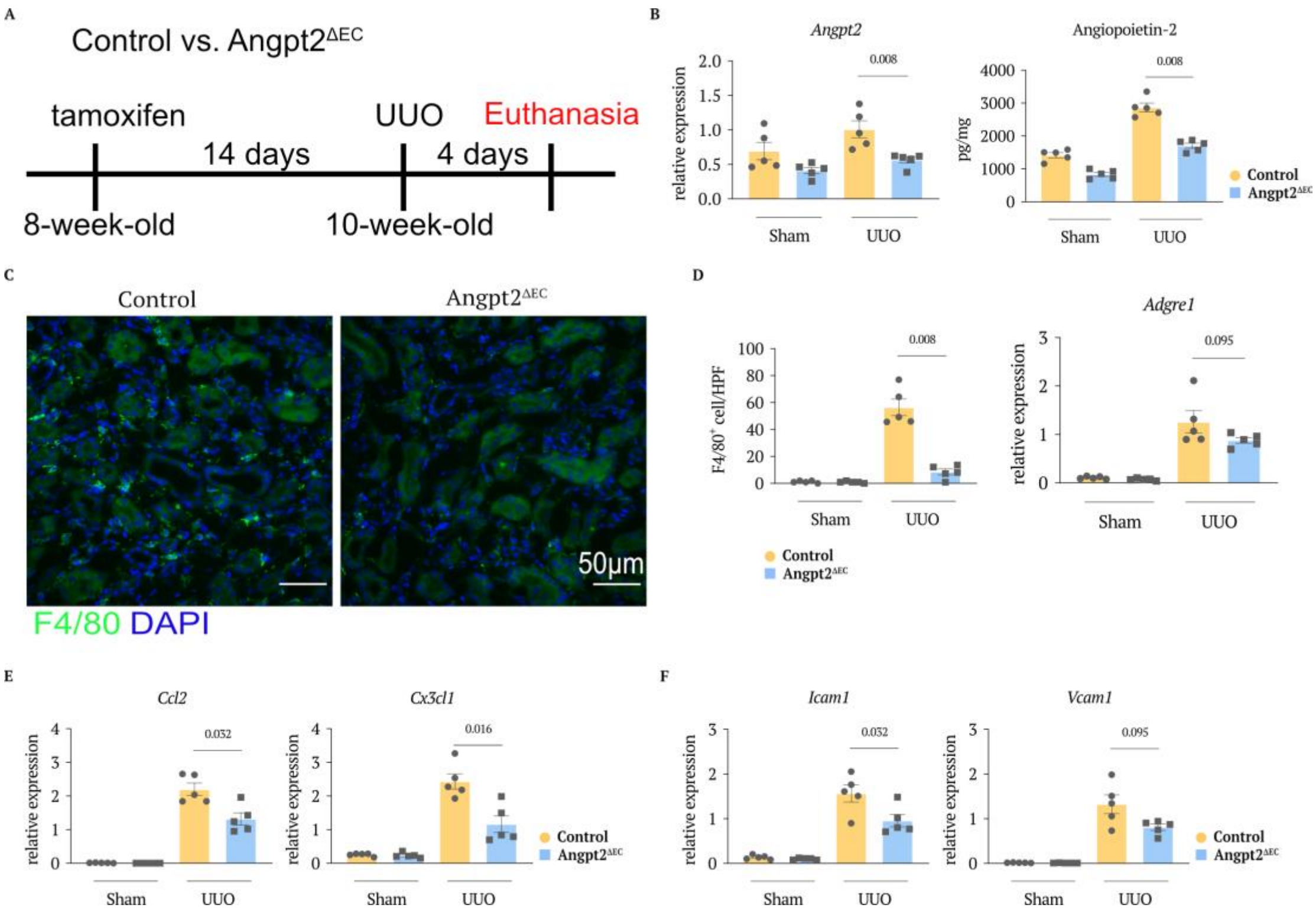

Supplemental Figure 13. Endothelial cell-specific angiopoietin-2 deletion attenuates inflammatory phenotype in early UUO-induced CKD. (**A**) Schematic diagram illustrating the experimental protocol for tamoxifen administration and UUO-induced kidney fibrosis in Control and Angpt2$^{\Delta EC}$ mice. Littermates lacking Cre served as controls (Control), while sham-operated mice served as healthy controls (Sham). Mice were analyzed on day 4 after surgery. (**B**) Bar graphs showing the relative expression levels of the *Angpt2* gene (left panel, normalized to *Gapdh*) and angiopoietin-2 protein (right panel, normalized to kidney weight) in the kidneys. (**C**, **D**) Representative images showing F4/80$^+$ macrophages in kidney sections from UUO-operated mice in **C**. Nuclei were counterstained with DAPI. Scale bar: 50 µm, original magnification 400×. Bar graph indicated the number of F4/80$^+$ macrophages per HPF at 400× magnification in **D**. (**E**, **F**) Bar graphs depicting the relative expression levels of pro-inflammatory genes (**E**) and adhesion molecule-encoding genes (**F**), normalized to *Gapdh*, in the kidneys. Data are expressed as means ± SEM,

with each dot representing an individual mouse. Statistical significance was determined using two-tailed Mann-Whitney U test. $n = 5$ mice per group.

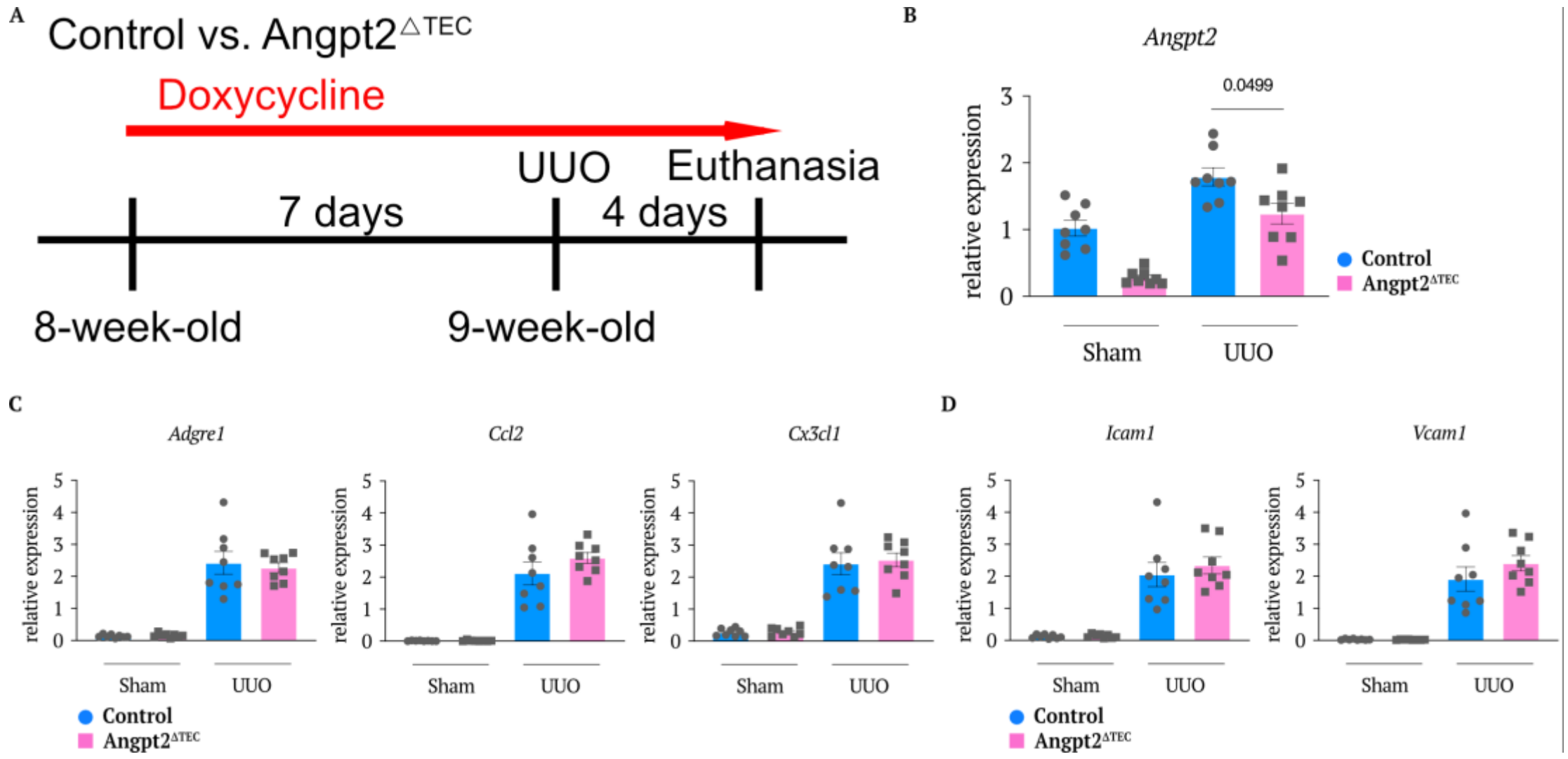

Supplemental Figure 14. Tubular epithelial cell-specific angiopoietin-2 deletion does not affect inflammatory phenotype in early UUO-induced CKD. (**A**) Schematic diagram illustrating the experimental protocol for doxycycline administration and UUO-induced kidney fibrosis in control and Angpt2$^{\Delta TEC}$ mice. Mice that underwent a sham operation served as healthy controls (Sham). Mice were analyzed on day 4 after the surgery. (**B**) Bar graph showing the relative expression levels of *Angpt2* gene, normalized to *Gapdh* in the kidneys. (**C**, **D**) Bar graphs depicting the relative expression levels of pro-inflammatory genes (**C**) and adhesion molecule-encoding genes (**D**), normalized to *Gapdh*, in the kidneys. Data are presented as means ± SEM, with each dot representing an individual mouse. Statistical significance was determined using two-tailed Mann-Whitney U test. $n = 5$ mice per group.

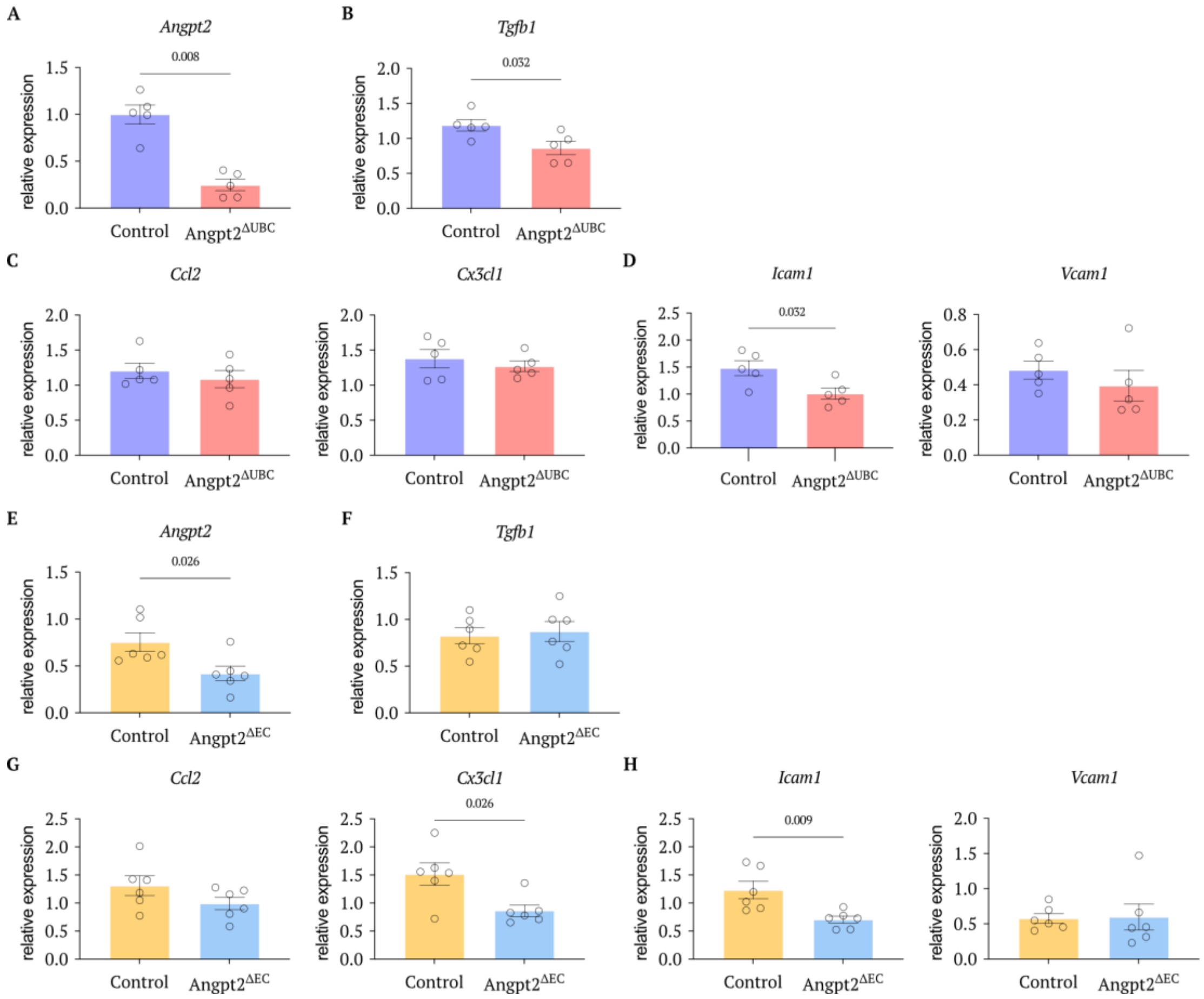

Supplemental Figure 15. Global and endothelial cell-specific angiopoietin-2 deletion attenuates pro-inflammatory and pro-fibrotic phenotypes in endothelial cells during early UUO-induced CKD. (**A-D**) Bar graphs showing the relative expression levels of *Angpt2*, *Tgfb1*, pro-inflammatory genes, and adhesion molecule-encoding genes, normalized to *Gapdh*, in kidney tissues from Control and Angpt2$^{\Delta UBC}$ mice. (**E-H**) Bar graphs showing the relative expression levels of *Angpt2*, *Tgfb1*, pro-inflammatory genes, and adhesion molecule-encoding genes, normalized to *Gapdh*, in kidney tissues from Control and Angpt2$^{\Delta EC}$ mice. Data are expressed as means ± SEM, with each dot representing an individual mouse. Statistical significance was determined using two-tailed Mann-Whitney U test. n = 5 mice per group in **A–D** and n = 6 mice per group in **E–H**.

Supplemental Figure 16. Pathology report of a male *Angpt2$^{-/-}$* mouse.

國立臺灣大學醫學院
National Taiwan University College of Medicine

PATHOLOGY REPORT

NTUCM Laboratory Animal Center

NTU CMLAC 實驗動物中心

Accession number: 249　　Pathological number: 109010　　Pathologist: Tsai

Received date: 109/2/19　　Species: Mice　　Age: 10-week-old　　Sex: Male

Strain: Angpt2 (homozygous)　　Alive/Dead: Alive　　Owner: 林　　Room: 8F 十號房

1. History:

No significant clinical signs were observed.

2. Gross findings:

The mouse presented testicular edema (arrow a, Fig. 1).

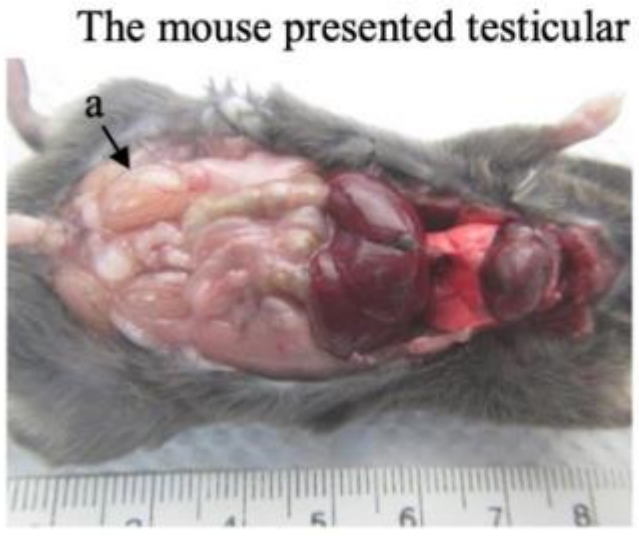

Fig. 1

3. Histopathological examination:

The lymph nodes revealed ectasia (arrow b) of lymphatic sinuses (Fig. 2-3). The testes presented interstitial edema (arrow c) (Fig. 4). No significant histopathological changes were noted in the brain (Fig. 5), heart (Fig. 6), liver (Fig. 7), spleen (Fig. 8), lung (Fig. 9) and kidney (Fig. 10).

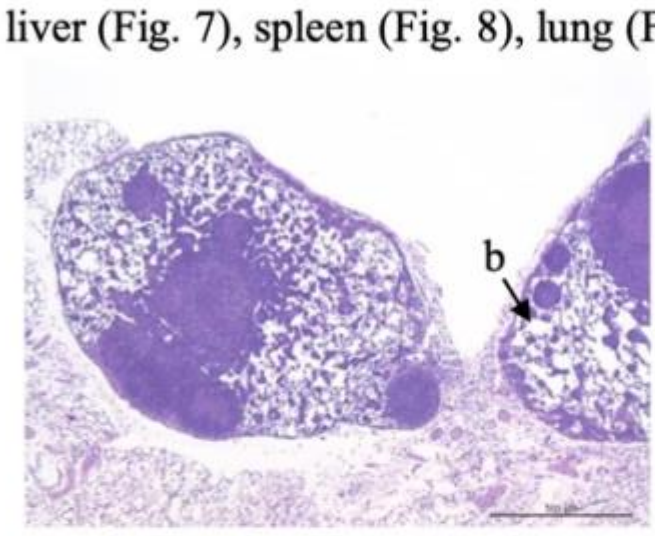

Fig. 2

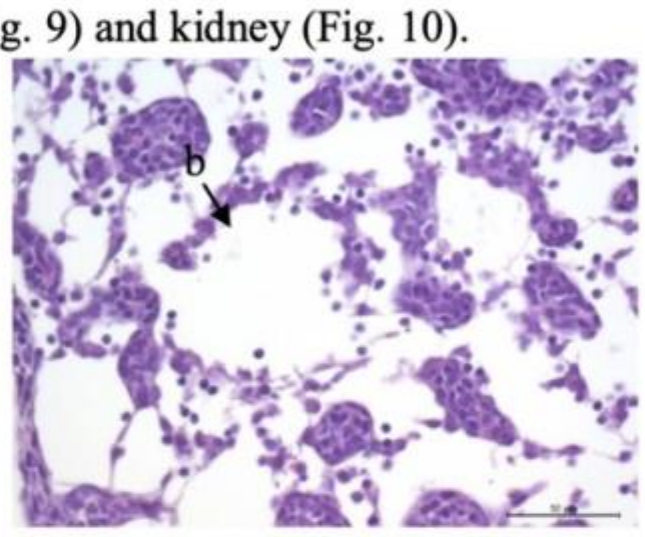

Fig. 3

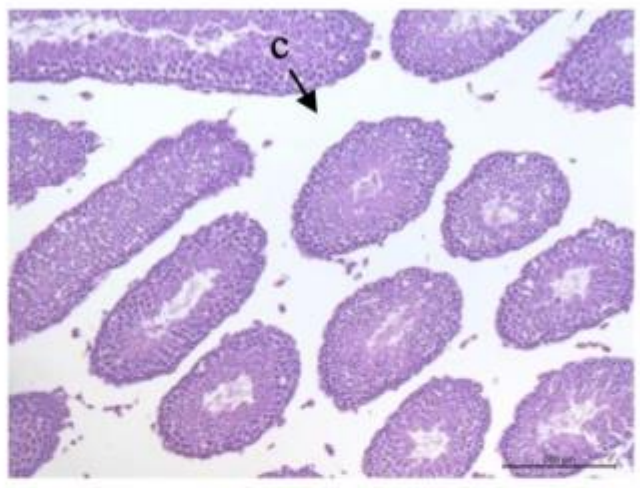

Fig. 4

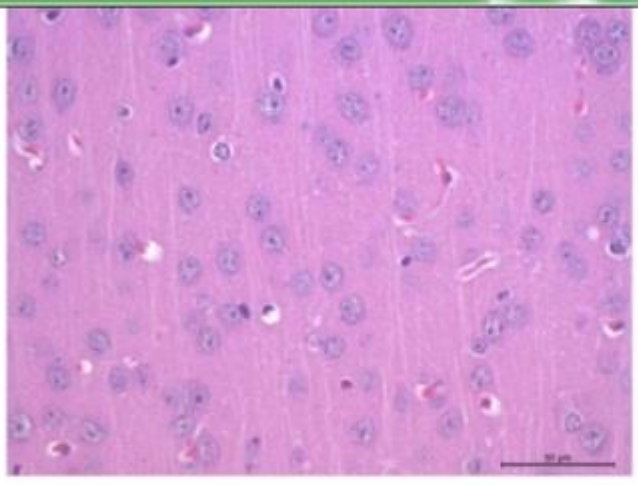

Fig. 5

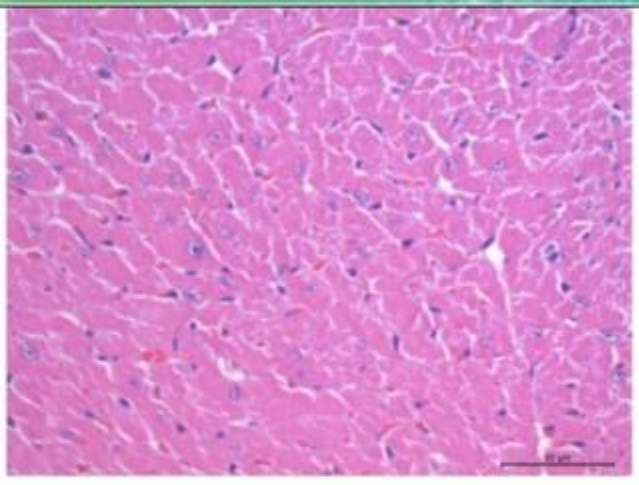

Fig. 6

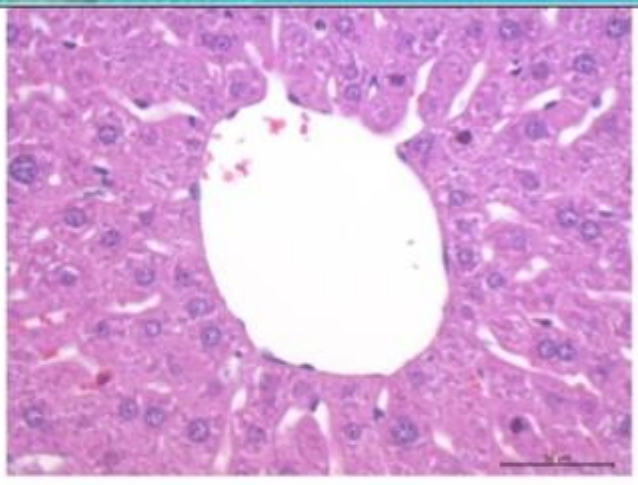

Fig. 7

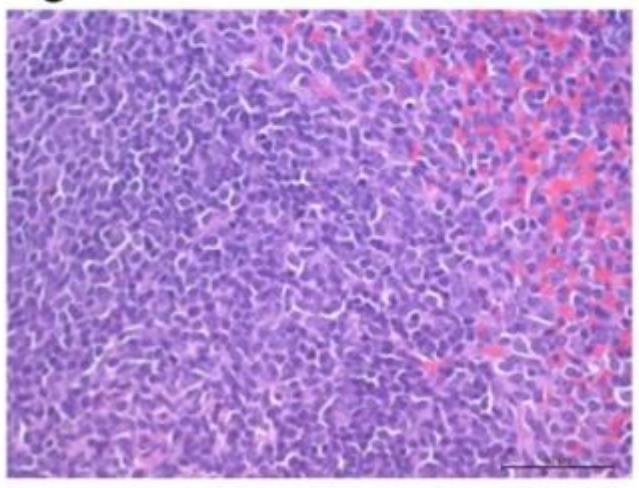

Fig. 8

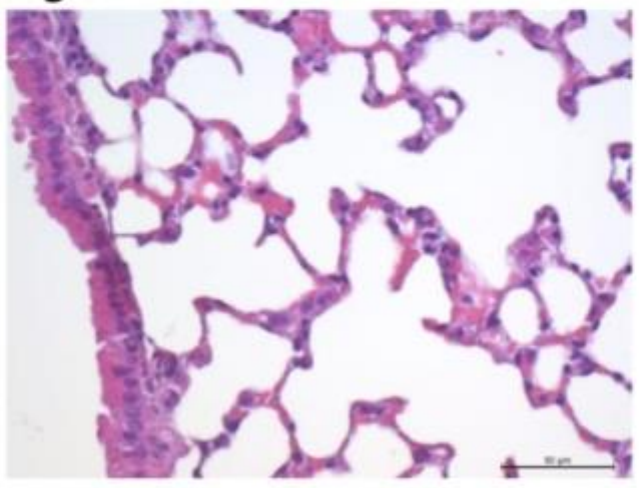

Fig. 9

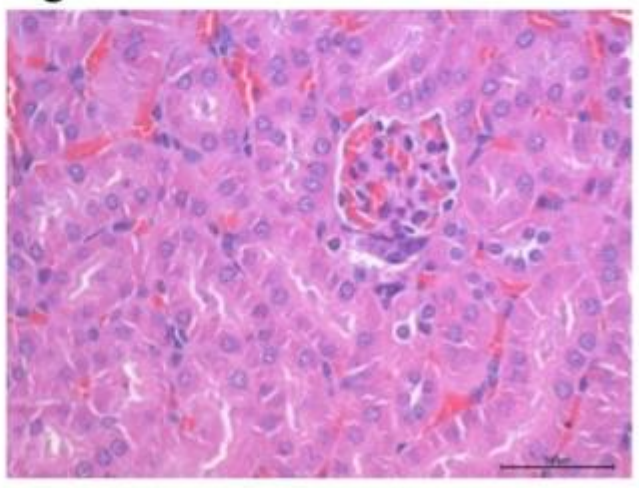

Fig. 10

4. Morphological diagnosis:

A. Lymph nodes

Ectasia, lymphatic sinuses, severe, diffuse, lymph nodes

B. Testes

Edema, interstitial, severe, diffuse, testes

5. Comments:

Molecular mechanisms regulating the remodeling of the lymphatic vasculature from an immature plexus of vessels to a hierarchal network of initial and collecting lymphatics are not well understood. One gene thought to be important for this process is Angiopoietin-2 (Ang-2). Ang2-/- mice have previously been reported to exhibit an abnormal lymphatic phenotype but the precise nature of the lymphatic defects and the underlying mechanisms have yet to be defined [(1)]. In this case, the ectasia of lymphatic sinuses and testicular interstitial edema should result from lymphatic defects, which were related to the genotype.

6. References:

(1) Dellinger M, Hunter R, Bernas M, Gale N, Yancopoulos G, Erickson R, Witte M. Defective remodeling and maturation of the lymphatic vasculature in Angiopoietin-2 deficient mice. Developmental Biology 319: 309-320, 2008.

Supplemental Figure 17. Pathology report of a female *Angpt2*$^{-/-}$ mouse.

國立臺灣大學醫學院
National Taiwan University College of Medicine

PATHOLOGY REPORT

NTUCM Laboratory Animal Center

NTU CMLAC
實驗動物中心

Accession number: 242 Pathological number: 109009 Pathologist: Tsai
Received date: 109/2/19 Species: Mice Age: 11-week-old Sex: Female
Strain: Angpt2 (homozygous) Alive/Dead: Alive Owner: 林 Room: 8F 十號房

1. History:

No significant clinical signs were observed.

2. Gross findings:

No significant gross findings were observed (Fig. 1).

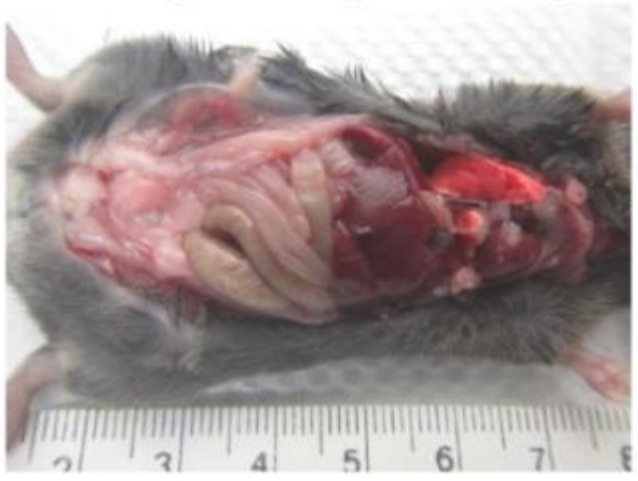

Fig. 1

3. Histopathological examination:

The lymph nodes revealed ectasia (arrow a) of lymphatic sinuses (Fig. 2-3). No significant histopathological changes were noted in the brain (Fig. 4), heart (Fig. 5), liver (Fig. 6), spleen (Fig. 7), lung (Fig. 8) and kidney (Fig. 9).

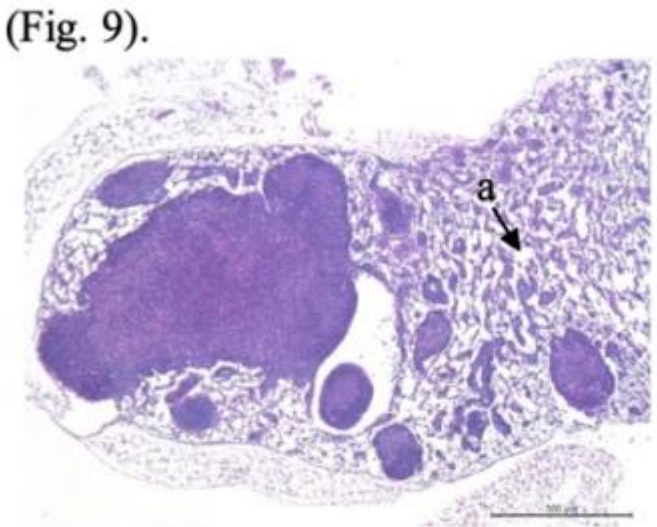

Fig. 2

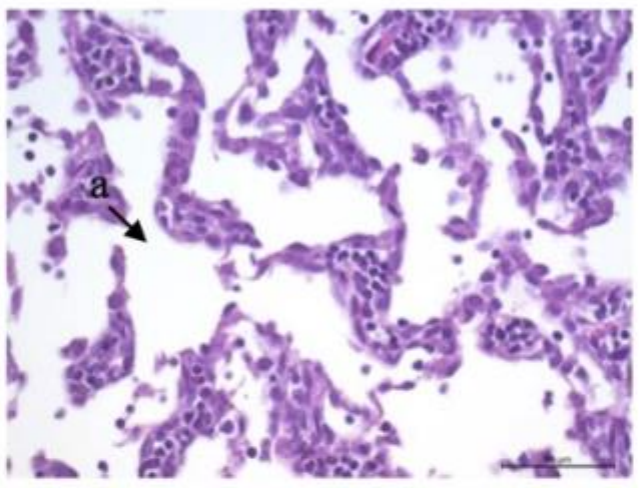

Fig. 3

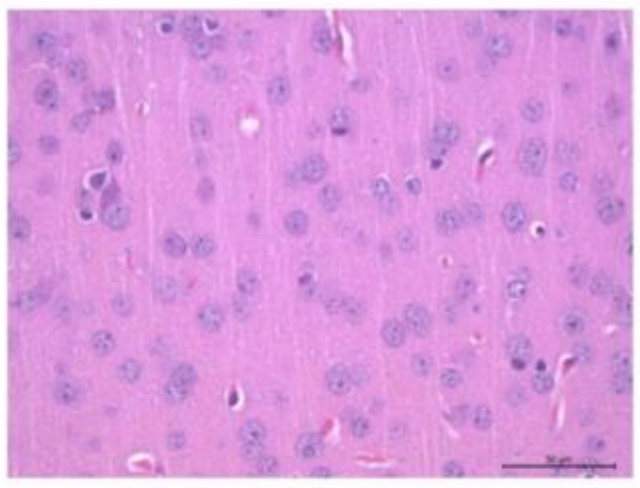

Fig. 4

Supplemental Figure 18. Pathology report of a male *Angpt2*$^{loxP/-}$ mouse.

國立臺灣大學醫學院
National Taiwan University College of Medicine

PATHOLOGY REPORT

NTUCM Laboratory Animal Center

NTU CMLAC

實驗動物中心

Accession number: 248 Pathological number: 109011 Pathologist: Tsai

Received date: 109/2/19 Species: Mice Age: 10-week-old Sex: Male

Strain: Angpt2 (heterogeneous) Alive/Dead: Alive Owner: 林 Room: 8F 十號房

1. History:

No significant clinical signs were observed.

2. Gross findings:

No significant gross findings were observed (Fig. 1).

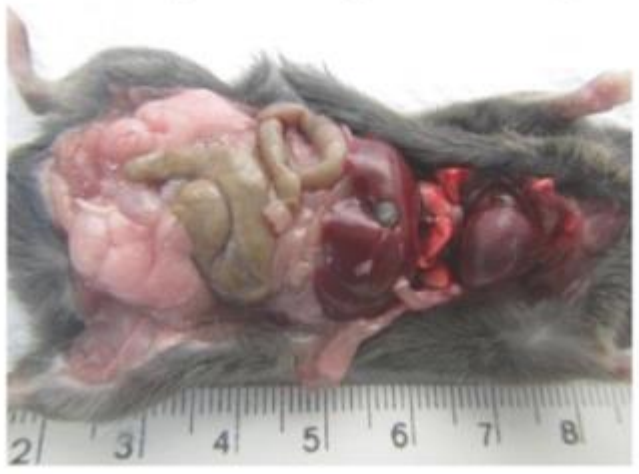

Fig. 1

3. Histopathological examination:

No significant histopathological changes were noted in the lymph nodes (Fig. 2-3), testes (Fig. 4), brain (Fig. 5), heart (Fig. 6), liver (Fig. 7), spleen (Fig. 8), lung (Fig. 9) and kidney (Fig. 10).

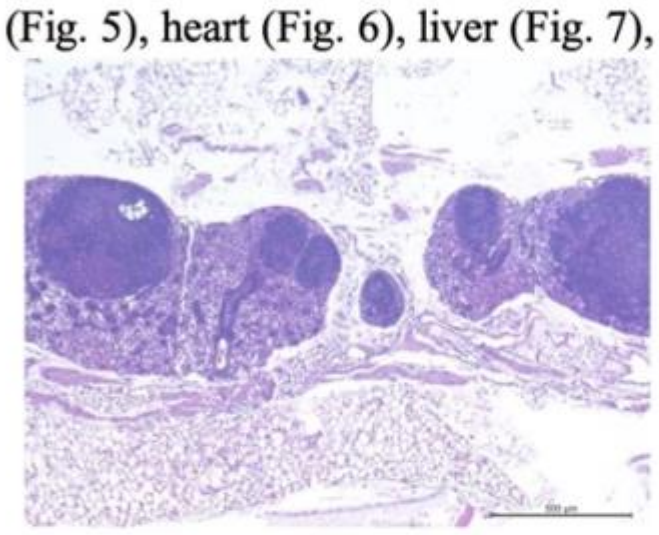

Fig. 2

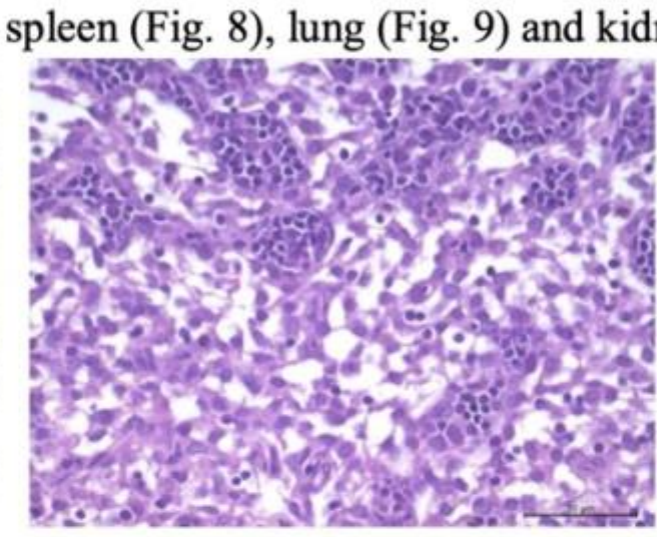

Fig. 3

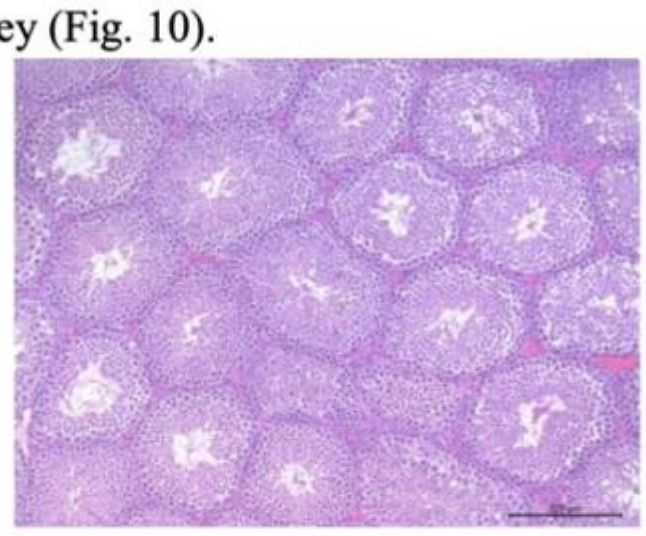

Fig. 4

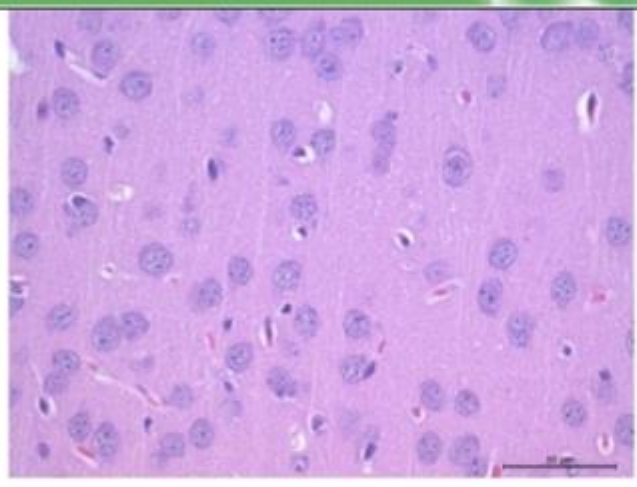
Fig. 5

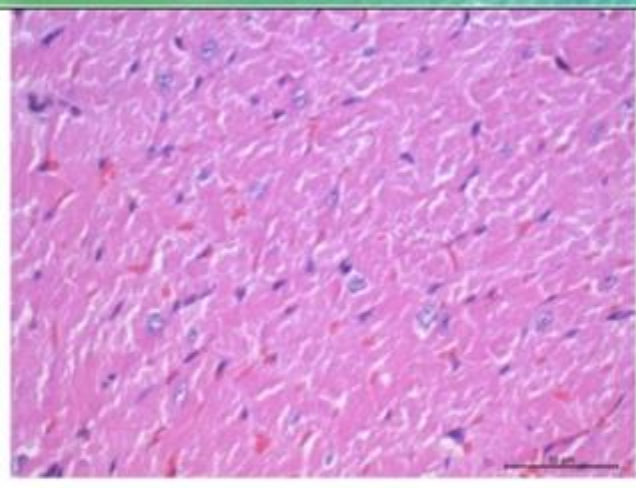
Fig. 6

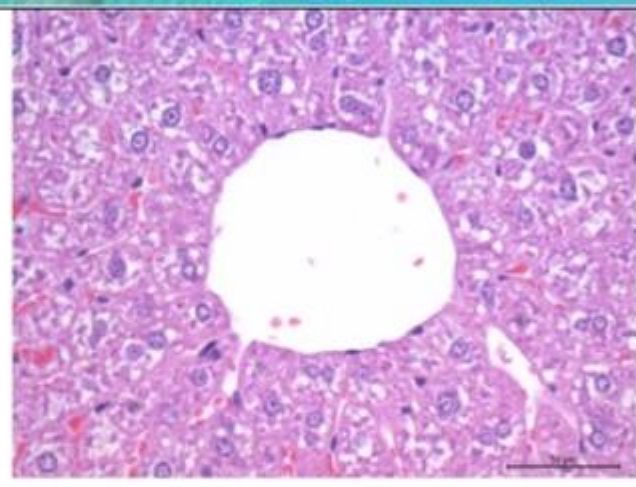
Fig. 7

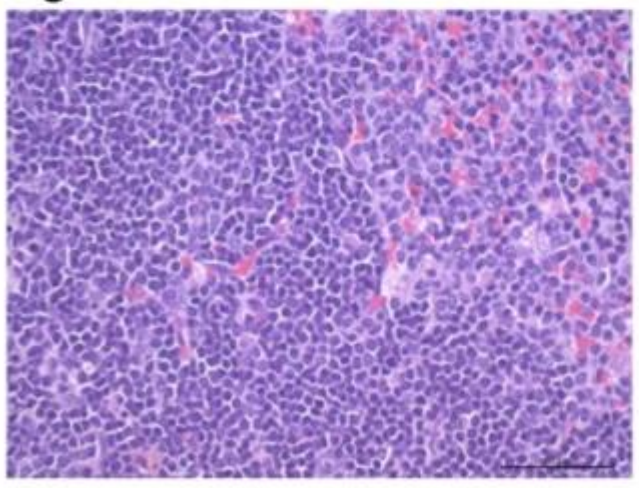
Fig. 8

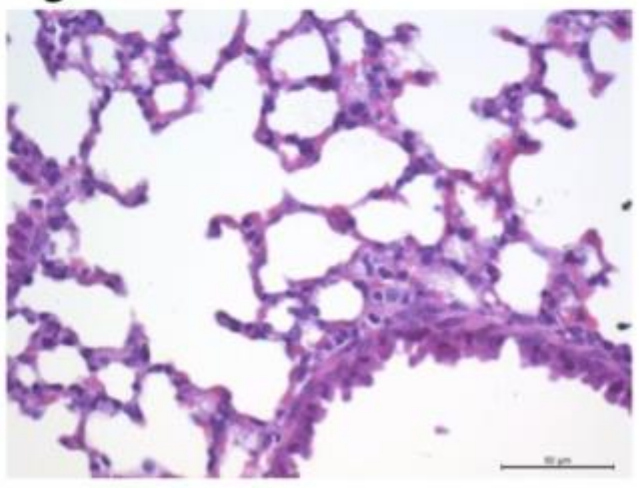
Fig. 9

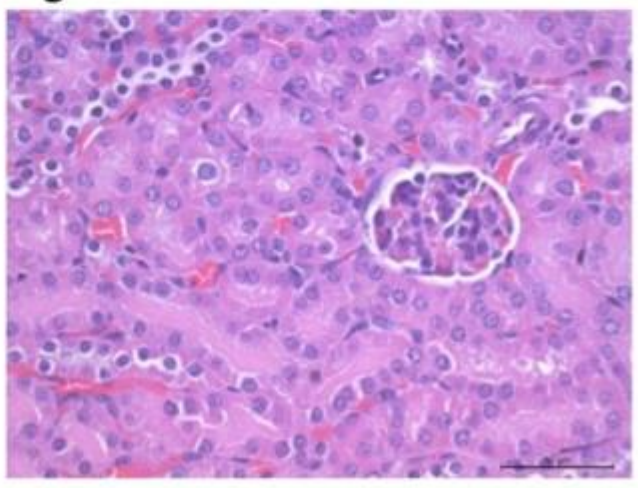
Fig. 10

4. Morphological diagnosis:
   No significant histopathological changes

Pathologist	Chief of Research Division	Attending veterinarian	Director of NTUCM Laboratory Animal Center

Supplemental Figure 19. Pathology report of a male *Angpt2*$^{loxP/loxP}$ mouse.

國立臺灣大學醫學院
National Taiwan University College of Medicine

PATHOLOGY REPORT

NTUCM Laboratory Animal Center

NTU CMLAC 實驗動物中心

Accession number: 244 Pathological number: 109012 Pathologist: Tsai
Received date: 109/2/19 Species: Mice Age: 10-week-old Sex: Male
Strain: Wild type Alive/Dead: Alive Owner: 林 Room: 8F 十號房

1. History:

No significant clinical signs were observed.

2. Gross findings:

No significant gross findings were observed (Fig. 1).

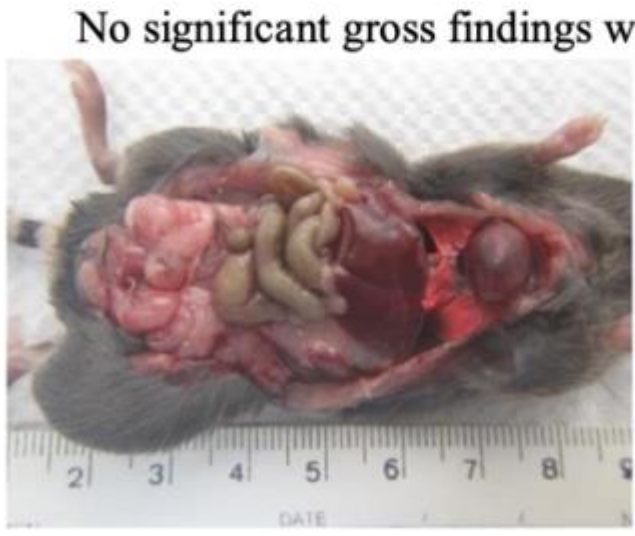

Fig. 1

3. Histopathological examination:

No significant histopathological changes were noted in the lymph nodes (Fig. 2-3), testes (Fig. 4), brain (Fig. 5), heart (Fig. 6), liver (Fig. 7), spleen (Fig. 8), lung (Fig. 9) and kidney (Fig. 10).

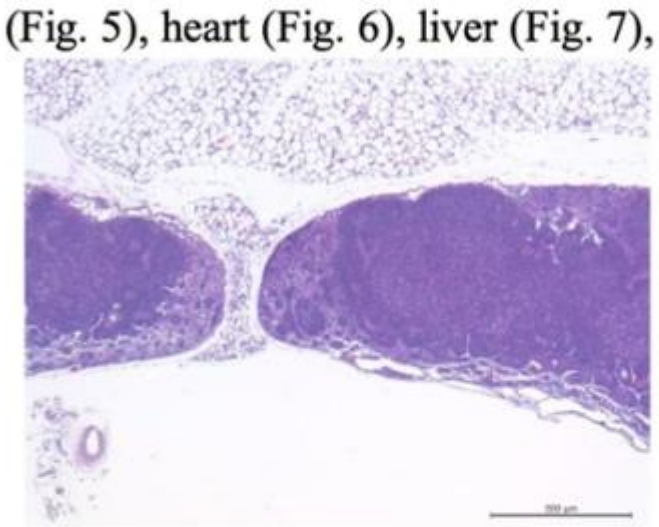

Fig. 2

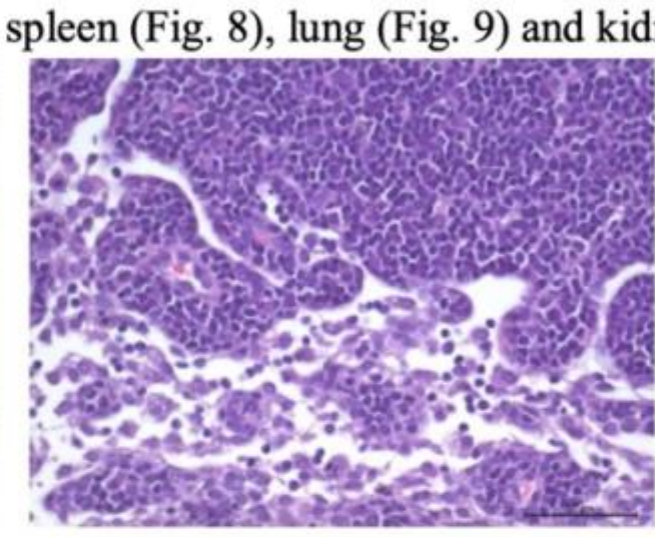

Fig. 3

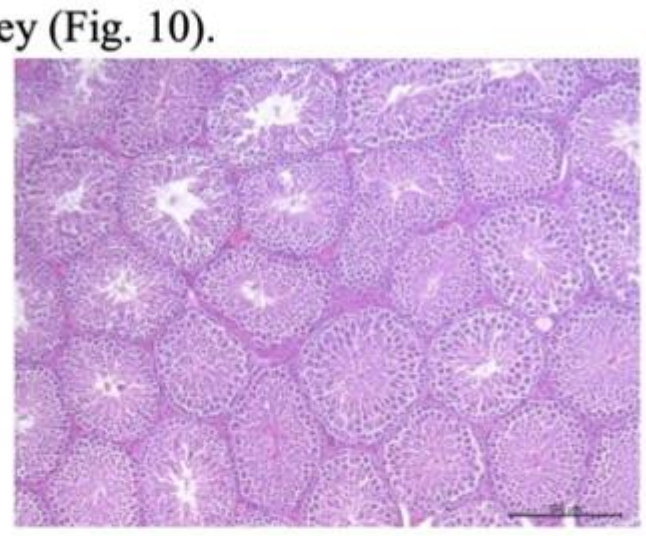

Fig. 4

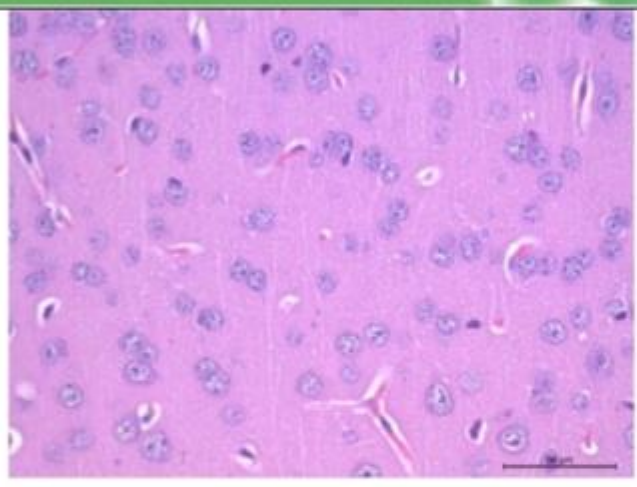
Fig. 5

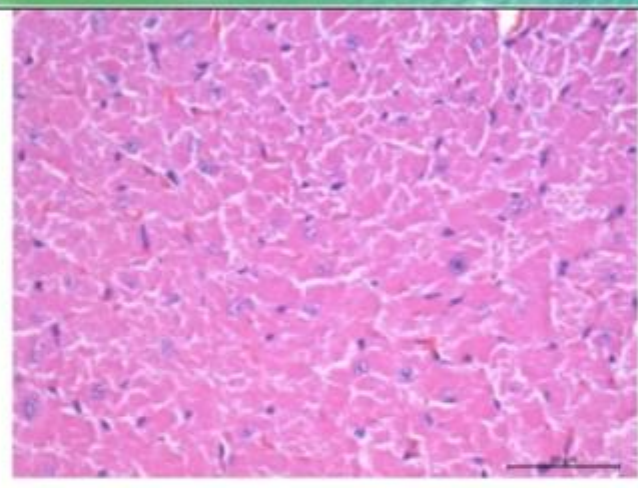
Fig. 6

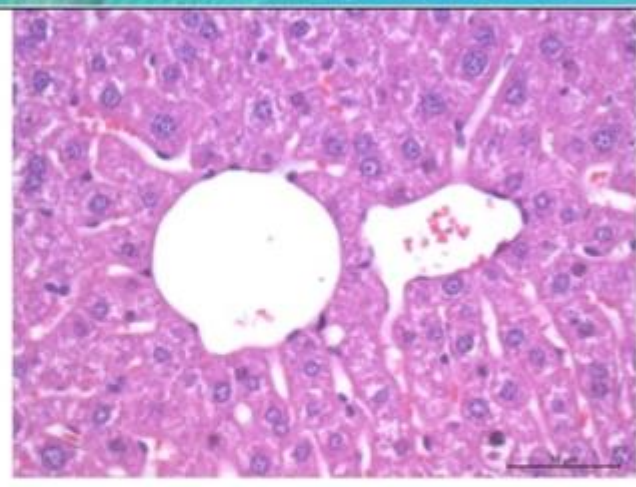
Fig. 7

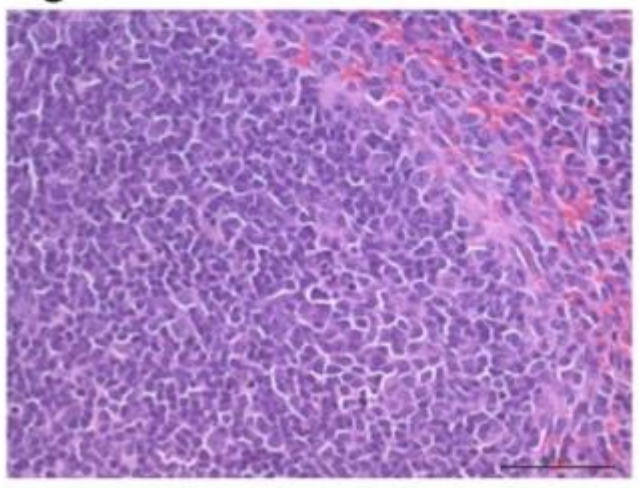
Fig. 8

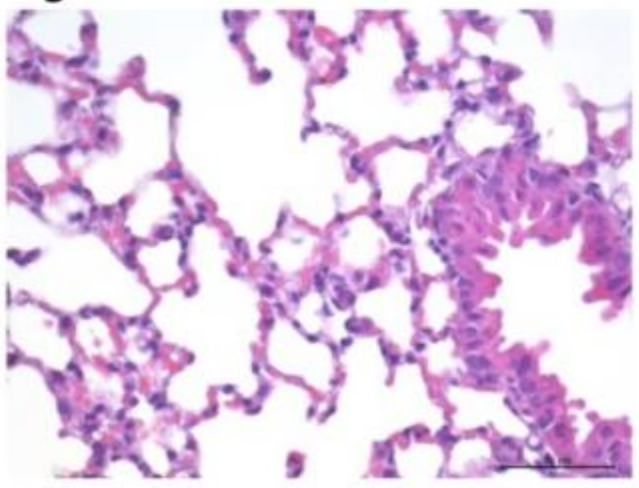
Fig. 9

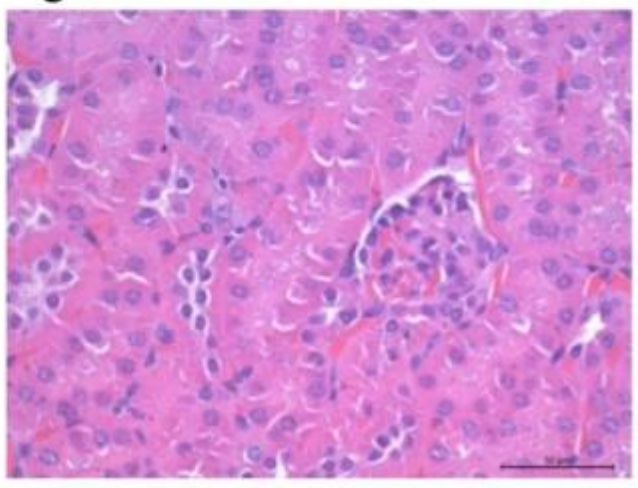
Fig. 10

4. Morphological diagnosis:
   No significant histopathological changes

Pathologist	Chief of Research Division	Attending veterinarian	Director of NTUCM Laboratory Animal Center

Supplemental Tables

Supplemental Table 1. Breeding summary of offspring at 7 days of age from *Angpt2*$^{loxP/–}$ parents.

	Angpt2$^{loxP/loxP}$	*Angpt2*$^{loxP/–}$	*Angpt2*$^{–/–}$	Total
Male	94 (39%)	141 (59%)	6 (2%)	241 (44%)
Female	92 (31%)	192 (64%)	17 (5%)	301 (56%)
Total	186 (34%)	333 (62%)	23 (4%)	542 (100%)

Values are n (%).

Supplemental Table 2. Characteristics of Control and Angpt2$^{\Delta UBC}$ mice two weeks after tamoxifen administration.

Genotype	Control	Angpt2$^{\Delta UBC}$
Body Weight (g)	23.66±2.33	21.60±2.39
Blood Pressure (mmHg)	122.84±11.28	120.14±10.54
BUN (mg/dL)	21.50±1.84	22.61±4.09
Cre (mg/dL)	0.10±0.00	0.10±0.00
Kidney Weight (g)	0.25±0.03	0.26±0.04
Kidney/Body Weight (%)	1.04±0.10	1.25±0.29
UACR (mg/g)	4.04±1.18	3.33±1.31
Glucose (mg/dL)	198.5±10.03	189.85±19.16
Triglyceride (mg/dL)	154.52±16.85	157.63±26.74

Data were expressed as means ± SEM (n = 5/group).

Supplemental Table 3. Characteristics of Control and Angpt2$^{\Delta TEC}$ mice two weeks after doxycycline administration.

Genotype	Control	Angpt2$^{\Delta TEC}$
Body Weight (g)	27.68±4.01	28.70±2.23
Blood Pressure (mmHg)	113.88±21.28	121.56±23.52
BUN (mg/dL)	23.01±6.10	23.29±5.63
Cre (mg/dL)	0.20±0.07	0.26±0.05
Kidney Weight (g)	0.24±0.02	0.24±0.01
Kidney/Body Weight (%)	0.87±0.07	0.85±0.06

Data were expressed as means ± SEM (n = 5/group).

Supplemental Table 4. Characteristics of Control and Angpt2$^{\Delta EC}$ mice two weeks after tamoxifen administration.

Genotype	Control	Angpt2$^{\Delta EC}$
Body Weight (g)	23.74±1.93	23.46±1.34
Blood Pressure (mmHg)	121.19±12.11	114.13±21.84
BUN (mg/dL)	30.26±3.85	32.68±8.01
Cre (mg/dL)	0.14±0.05	0.16±0.05
Kidney Weight (g)	0.21±0.02	0.21±0.02
Kidney/Body Weight (%)	0.89±0.15	0.87±0.07

Data were expressed as means ± SEM (n = 5/group).

Supplemental Table 5. Primer sequence for the construction of targeting vector.

Primers	Sequence (5’ to 3’)
AU4 37685	ATAGCGGCCGCTATCAGGGCCCTTCTATCT
BD5 37921	ATAAAGCTTATACCTAGGCCTGCTCTGTCACTACTTCC
CU2 43938	ATAGCGGCCGCGTGATCTACATTTCCCTCCA

DD2 44138	ATAGAATTCTGACTGTGAACCTTTCCTC
EU2 44139	ATAGGATCCGTTCTCCACTCTTTCATTCATC
FD2 44351	ATAGTCGACGATGCATTACCACTCACAGA
GU 46455	ATAGCGGCCGCCACAAGTTAGGAAGCCAT
HD 46690	CACGAATTCACCCCCCAGGAAGTAAA
IU 46691	ATAGGATCCGGAGGGAAGATGAGATTTTTC
JD 46915	ATAGAATTCCCTCCAGATTCTAATTCTGCCG
YU4 49249	ATAAAGCTTAGTTACACTGCTCTGGGAAC
ZD4 49449	ATAACTAGTAGAACTCAAGGTTTTGAACG

Supplemental Table 6. Primer sequence for quantitative PCR.

Gene	Sequence (5' to 3')
Ccl2	
Forward	AGCACCAGCCAACTCTCACT
Reverse	CGTTAACTGCATCTGGCTGA
Ccr2	
Forward	ATTCTCCACACCCTGTTTCG
Reverse	GATTCCTGGAAGGTGGTCAA
Cx3cl1	
Forward	TCGGACTTTGTTGGTTCCTC
Reverse	CAAAATGGCACAGACATTGG
Cx3cr11	
Forward	GGAGACTGGAGCCAACAGAG
Reverse	CCTGATCCAGGGAATGCTAA
Tgfb1	

Forward GGACTCTCCACCTGCAAGAC

Reverse GACTGGCGAGCCTTAGTTTG

Il1b

Forward CCCAAGCAATACCCAAAGAA

Reverse GCTTGTGCTCTGCTTGTGAG

Icam1

Forward AGCACCTCCCCACCTACTTT

Reverse AGCTTGCACGACCCTTCTAA

Vcam1

Forward TACCAGCTCCCAAAATCCTG

Reverse TCTGCTAATTCCAGCCTCGT

Angpt1

Forward GGAACCGAGCCTACTCACAG

Reverse TTAGATTGGAAGGGCCACAG

Angpt2

Forward CGGTCAACAACTCGCTCCTT

Reverse CGGATAGCAACCGAGCTCTT

Gapdh

Forward ACGGCCGCATCTTCTTGTGCA

Reverse AATGGCAGCCCTGGTGACCA

Ccl17

Forward AGTGGAGTGTTCAGGGATG

Reverse CTGGTCACAGGCCGTTTTAT

Ccl22

Forward TGGCTACCCTGCGTGTCCCA

Reverse	GCCAGGCTTGCGGCAGGATT
Pecam1	
Forward	AGCTCTGGGAACGAGAGCCACA
Reverse	CGGGACATGGACGACCTCCCA
Adgre1	
Forward	GCCACGGGGCTATGGGATGC
Reverse	ACCCACAGTGTCCAGGCAAGG
Acta2	
Forward	CTGACAGAGGCACCACTGAA
Reverse	CATCTCCAGAGTCCAGCACA
Col1a1	
Forward	GAGCGGAGAGTACTGGATCG
Reverse	GTTCGGGCTGATGTACCAGT
Col3a1	
Forward	ACCAAAAGGTGATGCTGGAC
Reverse	GACCTCGTGCTCCAGTTAGC

Supplemental Table 7. Primer sequence for genotyping PCR.

Mouse	Sequence (5’ to 3’)
Tg(Pax8-rtTA)	
Forward	CCATGTCTAGACTGGACAAGA
Reverse	CTCCAGGCCACATATGATTAG
Cre mice: *Tg(UBC-CreERT2)*, *Tg(Cdh5-CreERT2)*, *LC1*	
Forward	ATCCGAAAAGAAAACGTTGA
Reverse	ATCCAGGTAACGGATATAGT

Angpt2$^{loxP/loxP}$	
Forward	GTGATCTACATTTCCCTCCA
Reverse	GATGCATTACCACTCACAGA
Embryonic Angpt2 deletion	
Forward	GTGATCTACATTTCCCTCCA
Reverse	CCTCCAGATTCTAATTCTGCCG
